# Molecular mechanisms underlying hematophagia revealed by comparative analyses of leech genomes

**DOI:** 10.1093/gigascience/giad023

**Published:** 2023-04-11

**Authors:** Jinghui Zheng, Xiaobo Wang, Tong Feng, Saif ur Rehman, Xiuying Yan, Huiquan Shan, Xiaocong Ma, Weiguan Zhou, Wenhua Xu, Liying Lu, Jiasheng Liu, Xier Luo, Kuiqing Cui, Chaobin Qin, Weihua Chen, Jun Yu, Zhipeng Li, Jue Ruan, Qingyou Liu

**Affiliations:** Guangdong Provincial Key Laboratory of Animal Molecular Design and Precise Breeding, School of Life Science and Engineering, Foshan University, Foshan 528225, China; Department of Cardiology, Ruikang Hospital Affiliated to Guangxi University of Chinese Medicine, Nanning 530011, China; State Key Laboratory for Conservation and Utilization of Subtropical Agro-bioresources, Guangxi University, Nanning 530004, China; State Key Laboratory for Conservation and Utilization of Subtropical Agro-bioresources, Guangxi University, Nanning 530004, China; Department of Bioinformatics and Systems Biology, College of Life Science and Technology, Huazhong University of Science and Technology, Wuhan, Hubei 430074, China; State Key Laboratory for Conservation and Utilization of Subtropical Agro-bioresources, Guangxi University, Nanning 530004, China; State Key Laboratory for Conservation and Utilization of Subtropical Agro-bioresources, Guangxi University, Nanning 530004, China; State Key Laboratory for Conservation and Utilization of Subtropical Agro-bioresources, Guangxi University, Nanning 530004, China; Department of Cardiology, Ruikang Hospital Affiliated to Guangxi University of Chinese Medicine, Nanning 530011, China; Biological Institute of Guangxi Academy of Sciences, Nanning 530007, China; Department of Cardiology, Ruikang Hospital Affiliated to Guangxi University of Chinese Medicine, Nanning 530011, China; Department of Cardiology, Ruikang Hospital Affiliated to Guangxi University of Chinese Medicine, Nanning 530011, China; Department of Cardiology, Ruikang Hospital Affiliated to Guangxi University of Chinese Medicine, Nanning 530011, China; Guangdong Provincial Key Laboratory of Animal Molecular Design and Precise Breeding, School of Life Science and Engineering, Foshan University, Foshan 528225, China; Genome Analysis Laboratory of the Ministry of Agriculture, Agricultural Genomics Institute, Chinese Academy of Agricultural Sciences, Shenzhen, Guangdong 518120, China; Guangdong Provincial Key Laboratory of Animal Molecular Design and Precise Breeding, School of Life Science and Engineering, Foshan University, Foshan 528225, China; State Key Laboratory for Conservation and Utilization of Subtropical Agro-bioresources, Guangxi University, Nanning 530004, China; State Key Laboratory for Conservation and Utilization of Subtropical Agro-bioresources, Guangxi University, Nanning 530004, China; Department of Bioinformatics and Systems Biology, College of Life Science and Technology, Huazhong University of Science and Technology, Wuhan, Hubei 430074, China; CAS Key Laboratory of Genome Sciences and Information, Beijing Institute of Genomics, Chinese Academy of Sciences, Beijing 100101, China; State Key Laboratory for Conservation and Utilization of Subtropical Agro-bioresources, Guangxi University, Nanning 530004, China; Genome Analysis Laboratory of the Ministry of Agriculture, Agricultural Genomics Institute, Chinese Academy of Agricultural Sciences, Shenzhen, Guangdong 518120, China; Guangdong Provincial Key Laboratory of Animal Molecular Design and Precise Breeding, School of Life Science and Engineering, Foshan University, Foshan 528225, China; State Key Laboratory for Conservation and Utilization of Subtropical Agro-bioresources, Guangxi University, Nanning 530004, China

## Abstract

**Background:**

Leeches have been used in traditional Chinese medicine since prehistoric times to treat a spectrum of ailments, but very little is known about their physiological, genetic, and evolutionary characteristics.

**Findings:**

We sequenced and assembled chromosome-level genomes of 3 leech species (bloodsucking *Hirudo nipponia* and *Hirudinaria manillensis* and nonbloodsucking *Whitmania pigra*). The dynamic population histories and genome-wide expression patterns of the 2 bloodsucking leech species were found to be similar. A combined analysis of the genomic and transcriptional data revealed that the bloodsucking leeches have a presumably enhanced auditory sense for prey location in relatively deep fresh water. The copy number of genes related to anticoagulation, analgesia, and anti-inflammation increased in the bloodsucking leeches, and their gene expressions responded dynamically to the bloodsucking process. Furthermore, the expanded *FBN1* gene family may help in rapid body swelling of leeches after bloodsucking, and the expanded *GLB3* gene family may be associated with long-term storage of prey blood in a leech's body.

**Conclusions:**

The high-quality reference genomes and comprehensive datasets obtained in this study may facilitate innovations in the artificial culture and strain optimization of leeches.

## Introduction

Leeches are obligate bloodfeeding annelids distributed from tropical to subarctic regions around the globe. Hematophagous species, such as bats, ticks, and mosquitoes, are the most versatile vectors capable of transmitting a wide range of pathogens (such as protozoa, bacteria, nematodes, fungi, and viruses) to humans, livestock, and wildlife [1]; however, leeches have been found to transmit few infectious diseases. Furthermore, leeches have been used in traditional Chinese medicine since prehistoric times to treat a spectrum of ailments. Leeches secrete the most potent natural thrombin inhibitor, hirudin [2], and exhibit a variety of fascinating behavioral and physiological characteristics that are of interest from an evolutionary, biochemical, and pharmaceutical point of view. Leeches have also developed persistent adaptive strategies and characteristics to perceive their environment during long-term evolution. Leeches continuously receive sensory information from their surroundings by either mechanical or visual sensation to locate and target their prey. Additionally, the sanguivorous behavior of leeches is capable of reducing natural host reflexes (blood coagulation, pain, and inflammation) during bloodsucking [3]. To understand prey localization, sanguivorous behavior, and medicinal value of leeches, fundamental knowledge of leech genomes and genetic diversity is necessary, and this would undoubtedly open new avenues for research on leech biology, host interactions, and control strategies at the molecular level. Heretofore, leech research was primarily focused on strain optimization, artificial culture, and identification and development of therapeutic strategies; however, well-annotated genomes or genetic data are still unavailable. Currently, the genomic data of several leech species have been published [3–7] but not to the chromosome level. Here we provide 3 high-quality leech genomes (*Hirudo nipponia* [NCBI:txid42736], *Hirudinaria manillensis* [NCBI:txid1348078], and *Whitmania pigra* [NCBI:txid486152]) and abundant transcriptomes that illustrate the gene expression dynamics of bloodsucking leeches, including anticoagulation, analgesic, and anti-inflammation, which would facilitate understanding at the genetic level and could be crucial for drug candidate prospecting.

## Results

### Genome assemblies

**Figure 1:**
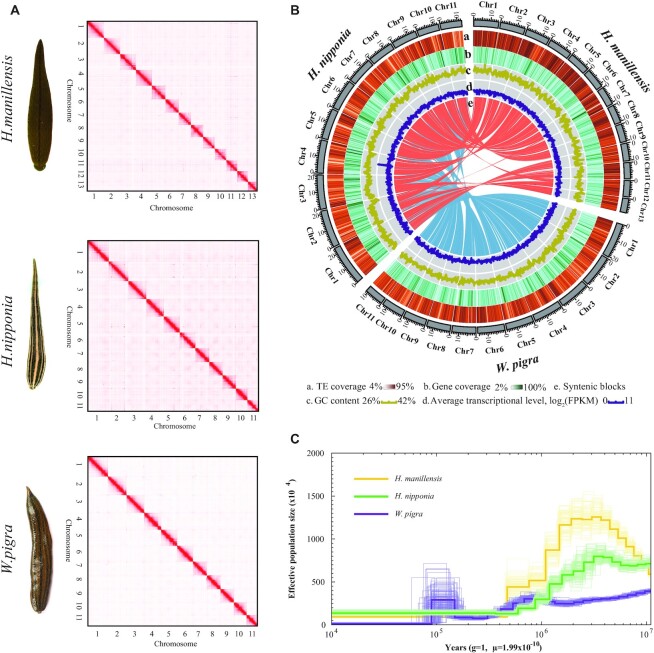
Genome assembly of the 3 leech species. (A) Hi-C interactive heatmap for genome-wide organization of the 3 leech species. (B) Comparative genomic analysis of the 3 leech species. Circos diagram depicts genome characteristics. Tracks from the outer to inner circles indicate the following: chromosomes, TE coverage, gene coverage, GC content, gene expression, and syntenic block (*H. nipponia* as reference). (C) Demographic history inferred using pairwise sequentially Markovian coalescent analysis.

We used the Nanopore platform for sequencing and performed genome assembly for 3 leech species, *H. nipponia, H. manillensis*, and *W. pigra* (Fig. 1A), that are ubiquitously used in the Chinese pharmacopeia. The assemblies for *H. nipponia, H. manillensis*, and *W. pigra* contained 985, 622, and 437 contigs with N50 contig lengths of 1.1, 2.5, and 4.1 Mb, respectively (Table 1). On the basis of the Hi-C data, the contigs of *H. nipponia, H. manillensis*, and *W. pigra* were consolidated into scaffolds with N50 lengths of 18.5, 11.9, and 16.2 Mb, respectively, and they comprised 11, 13, and 11 pseudo-chromosomes, respectively (Table 1 and Fig. 1B). The sizes of the final genome assemblies of *H. nipponia, H. manillensis*, and *W. pigra* were approximately 203.7, 157.5, and 181.4 Mb, respectively; the results were similar to the estimated genome sizes based on *k*-mer (K = 17) analysis (Supplementary Fig. S1). The assemblies had larger scaffold N50 sizes and lower scaffold numbers, indicating higher continuity than previously reported genomes (Supplementary Table S1).

**Table 1:** Summary statistics for the 3 leech genomes.

Genomic features	*H. nipponia*	*H. manillensis*	*W. pigra*
Total genome size (Mb)	203.7	157.5	181.4
Number of scaffolds^a^	11+253	13+243	11+183
Scaffold N50 (Mb)	18.5	11.9	16.2
Number of contigs	985	622	437
Contig N50 (Mb)	1.1	2.5	4.1
Number of genes	20,430	18,106	18,540
Repeat sequences	33.7%	25.3%	30.5%

a Number of chromosome-level scaffolds and unplaced scaffolds.

Furthermore, we aligned the short reads and transcriptome assemblies to the genomes to assess the completeness of our genome assemblies and found that more than 98% of the reads and 95% of transcriptome data were mapped to the assemblies (Supplementary Tables S2 and S3). We also estimated the completeness and accuracy of the final assemblies and found that 91.5%, 90.8%, and 91.7% of the BUSCO orthologs were captured for *H. nipponia, H. manillensis*, and *W. pigra*, respectively (Supplementary Table S4). We used Merqury [8] to obtain quality value scores of 35.8 for *W. pigra*, 33.4 for *H. nipponia*, and 32.1 for *H. manillensis*. We combined the results of the *de novo* and homolog-based approaches and identified about 25–33% of repetitive sequences in the leech genomes (Supplementary Table S5 and Supplementary Fig. S3).

### Population history of the leeches

We used pairwise sequentially Markovian coalescent analysis to infer changes in the effective population size (Ne) of the ancestral leech populations. The population of the nonbloodsucking leech *W. pigra* underwent 2 expansions, whereas the populations of the 2 bloodsucking leeches, *H. nipponia* and *H. manillensis*, experienced only 1 expansion (Fig. 1C). The different fluctuations in Ne may hint at different environmental adaptations of the 2 types of leeches. Interestingly, the Ne of the bloodsucking leeches began to decrease at the onset of the Pleistocene (∼2 million years ago (MYA)), which was characterized by repeated cycles of glaciations. The glaciations probably reduced the contact between leeches and animal hosts, resulting in a decline in the size of the bloodsucking leech populations.

### Gene annotation and gene family construction

Three methods—namely, *de novo*, homology-based, and transcriptome-based gene predictions—were used to identify a total of 20,430, 18,106, and 18,540 protein-coding genes in the *H. nipponia, H. manillensis*, and *W. pigra* genomes, and the total Coding Sequence (CDS) lengths were 32.3, 27.4, and 32.3 Mb and mean CDS lengths were 1,739, 1,647, and 1,801 bp, respectively (Supplementary Tables S6–S8). Further, the gene sets were aligned against UniProt, InterPro, and KEGG databases, and about 88% of the genes were functionally assigned or annotated (Supplementary Table S9). Besides, thousands of noncoding RNA (ncRNA) genes and secreted genes were also identified in each of the 3 leech genomes (Supplementary Tables S10–S13).

**Figure 2:**
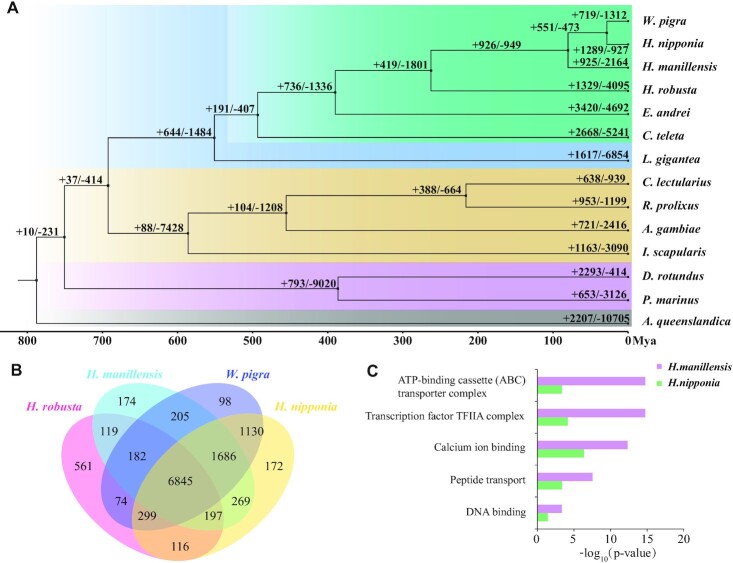
Phylogenetic tree and gene family analysis. (A) Phylogenetic tree generated using single-copy orthologous genes. Numbers on the nodes are the numbers of expanded (+) and contracted (−) gene families. (B) Venn diagram showing the numbers of detected orthologous gene families of 4 leech species. (C) GO analysis of the shared expanded gene families of the 2 bloodsucking leech species.

We constructed gene families and performed a phylogenetic analysis of 14 species. *W. pigra* and *H. nipponia* shared a common ancestor about 50 MYA, whereas *H. manillensis* diverged at an earlier date (Fig. 2A). This implies that the bloodsucking behavior may have existed in the ancestors of leeches, but this behavior was lost in the lineage of *W. pigra*. Four leech species were found to share most of their gene families, which is consistent with evolutionary relationships (Fig. 2B). Additionally, a total of 1,289, 925, and 719 expanded and 927, 2,164, and 1,312 contracted gene families were identified in the *H. nipponia, H. manillensis*, and *W. pigra* genomes, respectively. The Gene Ontology (GO) analysis depicted that expanded gene families such as the ATP-binding cassette transporter complex, transcription factor IIA complex, and calcium ion binding functions were significantly enriched in both bloodsucking leech species (Fig. 2C).

### Transcriptome dynamics

**Figure 3:**
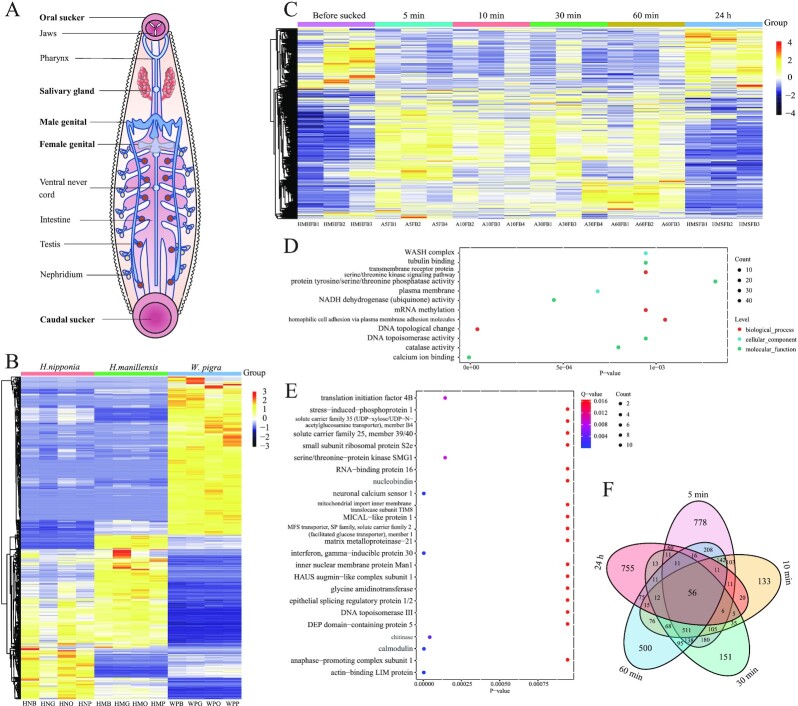
Transcriptome sequencing and analysis of the 3 leech species. (A) Anatomical diagram of a leech. The leech was divided into 4 parts according to the anatomical structure. (B) Heatmap of differentially expressed genes (DEGs) in the 3 leech species. (C) Heatmap of DEGs at different time points before bloodsucking, during bloodsucking (5–60 minutes), and after bloodsucking (24 hours) in *H. manillensis*. (D, E) GO and KEGG analyses of DEGs at different bloodsucking times in *H. manillensis*. (F) Venn diagram showing numbers of DEGs during different bloodsucking times in *H. manillensis*.

To analyze the gene expression patterns of the 3 leeches, we sequenced and analyzed the transcriptomes of 32 samples (3 replicates for each sample) of different developmental stages, tissues, and a series of bloodsucking behaviors at 5 different time points (Supplementary Figs. S5–S9). We used DEseq2 to identify differentially expressed genes (DEGs) between the nonbloodsucking and bloodsucking leeches and found that most of the DEGs of the 2 bloodsucking leeches shared similar expression patterns (Fig. 3B). We further investigated the transcriptomic dynamics during bloodsucking in *H. manillensis*. Mostly, the DEGs responded quickly after bloodsucking and continuously changed in the following 60 min (Fig. 3C, F). After 24 hours, the expression patterns of these DEGs were virtually restored to those of the prebloodsucking group (Fig. 3C). Furthermore, the GO and KEGG pathway analyses showed that most of the DEGs were significantly enriched to calcium, indicating that calcium-related regulation may play an important role in the bloodsucking behavior of leeches (Fig. 3D, E).

### Genetic basis of prey location for leeches

**Figure 4:**
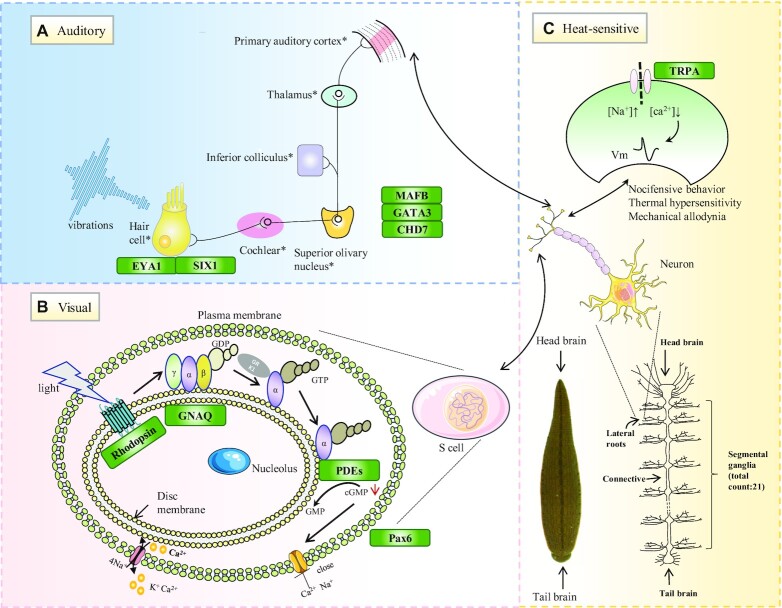
Molecular basis for prey location and tracing by leeches. Schematic diagram for the auditory system (A), visual system (B), and heat-sensitive channel (C) in leeches. On the basis of the genomic and transcriptional data, genes that encode known mechanical or visual receptors were identified in the 3 leech species. Genes identified in the leeches are labeled using green boxes.

Leeches are efficient predators because they can use their mechanical and auditory systems to acquire information and locate their prey. The associated genes were identified by homolog-based functional annotation in leeches (Fig. 4). Among the hearing-related genes of leeches (Fig. 4A), *SIX1* plays a crucial role in audio sensation, and we found a single copy of *SIX1* in the nonbloodsucking leeches and 2 or 4 copies of *SIX1* in the bloodsucking leeches (Supplementary Table S14). The expression pattern of *SIX1* was generally higher in the bloodsucking leeches than in the nonbloodsucking leeches (Supplementary Fig. S10), which clearly indicates that the bloodsucking leeches may possess better auditory perception.

Similarly, detection of visual signals in the eye is attributable to various mechanisms, with the coordinated involvement of genes and their related proteins or enzymes; intriguingly, *PDE6D*, which encodes the delta subunit of rod-specific photoreceptor phosphodiesterase, was detected in the nonbloodsucking leeches and not found in the bloodsucking leeches (Supplementary Table S14). Thus, we speculated that the bloodsucking leeches potentially strengthened audition and weakened vision to hide in relatively deep fresh water.

### Genetic basis of the sanguivorous behavior of bloodsucking leeches

**Figure 5:**
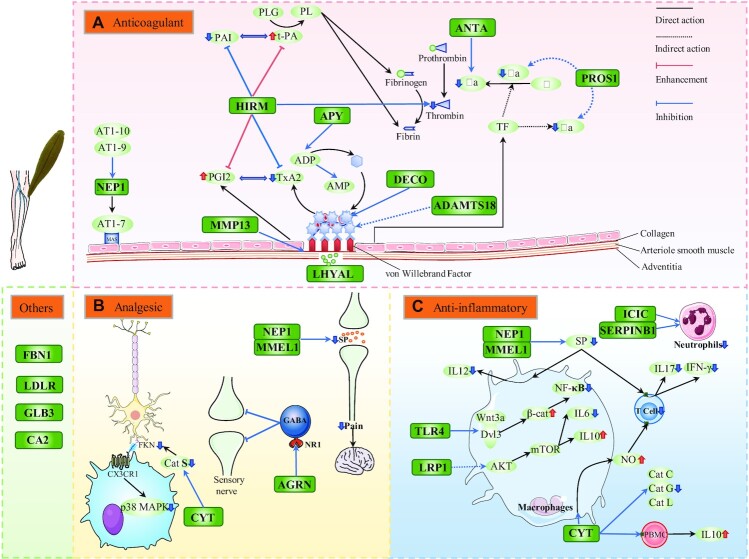
Molecular basis for the sanguivorous behaviors of leeches. Schematic diagram shows anticoagulation (A), analgesic (B), and anti-inflammatory (C) processes in leeches. The solid lines represent direct interaction, and the dotted lines represent indirect interactions. Genes identified in the leeches are labeled with green boxes.

Leeches avoid detection by hosts during the bloodsucking process by executing 3 key operations: inhibition of blood coagulation, suppression of inflammation, and alleviation of pain. We identified the genes related to each process (Fig. 5) and found that the total copy number of these genes was higher in the bloodsucking leeches than in the nonbloodsucking leeches (Supplementary Table S14).

Leeches inhibit hemagglutination mainly via 3 ways: suppressing the thrombin cascade (*HIRM1, ANTA*, and *PROS1*), inhibiting platelet aggregation (*DECO, MMP13, ADAMTS18, APY*, and *HIRM1*), and dilating vessels (*NEP1* and *HIRM1*) (Fig. 5A). The gene structure and copy number of the well-known anticoagulant *HIRM1* (hirudin) were different in the 3 leeches (Supplementary Fig. S11). The gene lengths of HN_hirudin and WP_hirudin were apparently longer than the 3 HM_hirudin copies, and this was mainly attributable to TE insertion into intron regions (Supplementary Fig. S11A). We performed multiple sequence alignment for 5 *HIRM1* genes and found that the cysteine pattern of hirudin [7] is more conserved than the tail but less conserved than the beginning of the alignment (Supplementary Fig. S11B). This indicates that the cysteine pattern may have the potential to be plastic. *HIRM1* was mainly expressed in the oral suckers of the 3 leech species, and the expression of HM_hiridin1 comparatively increased at 30 minutes (Supplementary Fig. S11C), which is consistent with the time of physiological coagulation.

We found that the expressions of 2 hyaluronidase (*LHYAL*) copies were the highest in only 5 minutes during the bloodsucking process (Supplementary Fig. S12). As expected, the gene expressions of 5 antistasin (*ANTA*) copies and 2 *PROS1* copies were relatively higher during the process of bloodsucking (5–60 minutes; Supplementary Fig. S12). Besides, all gene copies of *ADAMTS18* and *NEP1* showed peak expression levels in 10 minutes, suggesting that they may play a potentially pivotal role in the inhibition of hemagglutination.

During the bloodsucking process, leeches also produced agrin (*AGRN*), cystatin (*CYT*), neprilysin 1 (*NEP1*), and membrane metalloendopeptidase like 1 (*MMEL1*) (Fig. 5B), which may reduce pain sensitivity and help the leeches to avoid recognition by the host. Generally, all copies of *AGRN* and *MMEL1* as well as *CYT* showed high expressions during the bloodsucking process (Supplementary Fig. S11). Further analysis showed that the leeches also expressed various anti-inflammatory genes, such as *NEP1, MMEL1, CYT*, eglin (*ICIC*), cystatin (*CYT*), Leuko*CYT*e elastase inhibitor (*SERPINB1*), Toll-like receptor 4 (*TLR4*), and lipoprotein receptor-related protein 1 (*LRP1*) (Fig. 5C). Surprisingly, the expression levels of most *TLR4* and *LRP1* copies decreased quickly at the beginning of the bloodsucking process and increased after bloodsucking (24 hours; Supplementary Fig. S11). This indicates that leeches always maintain anti-inflammatory proteins for swift release into the prey body.

Moreover, we noted that *GLB3* and *FBN1* were the most expanded genes associated with sanguivorous behavior in the bloodsucking leeches. Seven or 8 copies of *GLB3* were tandemly arranged in the 2 bloodsucking leeches, with only 1 copy in *W. pigra* and no copy in *Helobdella robusta* (Supplementary Table S13 and Supplementary Fig. S13). Moreover, the expression levels of the *GLB3* family increased during the bloodsucking process. In particular, 3 *GLB3* copies displayed significant expression level changes after the bloodsucking process (Supplementary Fig. S12). This could presumably explain why leeches can store prey blood in their body for months. Twelve copies of *FBN1* were detected in the 2 bloodsucking species, and only 4 or 0 copies were found in the nonbloodsucking leeches (Supplementary Table S15). Generally, the *FBN1* family showed a continual increase in their expressions during the bloodsucking process (Supplementary Fig. S13), indicating that *FBN1* may be associated with the adaptability of leech body swelling after bloodsucking.

## Discussion

Precise nonredundant reference genomes with verified annotations are critical for functional as well as evolutionary analyses, and indeed, it is still a challenge to produce an accurate chromosome-level assembly, particularly for leech chromosomes. Although leeches have been used to treat diverse ailments since ancient times, most of our information on them is based on psychometrics. A comprehensive catalog of their genomes and gene expression patterns is fundamental to understanding the genetic basis of their behavior and will be crucial for drug candidate prospecting. Although the genome of the medicinal leech has been sequenced in several studies, the assembly results are fragmented [3–7]. However, in this study, we developed 3 chromosome-level genome assemblies for *H. nipponia, H. manillensis*, and *W. pigra* by integrating short-read sequencing, Nanopore sequencing, and Hi-C technology.

Leeches are efficient predators because of their specialized predation adaptation, with acute senses such as hearing, vision, and chemosensation. Leeches trace and locate their prey via mechanical and visual cues from water waves on the basis of S cells [9]. Ethological experiments have shown that leeches can quickly identify and locate the source of sound by analyzing the distribution of water waves [9]. Among hearing-related genes, *SIX1* mediates the relative numbers of sensory hair cells and statoacoustic ganglion neurons [10]. Overexpression of *SIX1* could result in more hair cells [10]. In our study, higher expression of *SIX1* in the bloodsucking leeches rather than the nonbloodsucking leeches clearly indicated that the bloodsucking leeches may possess better auditory perception. Furthermore, genes that encode opsin had very early origins and were recruited repeatedly during eye evolution [11]. The opsin family can be divided into 7 subfamilies, and rhodopsin and Gq-coupled opsin/melanopsin are the most abundant proteins in rod cells [12]. Phototransduction is initiated when rhodopsin absorbs photons and triggers the exchange of GDP for GTP on the G-protein, which leads to an increase in cGMP hydrolysis by the phosphodiesterase (PDE) complex [13]; surprisingly, *PDE6D*, which encodes the delta subunit of rod-specific photoreceptor phosphodiesterase, was present in the nonbloodsucking leeches but not in the bloodsucking leeches. We demonstrated that bloodsucking leeches possibly prefer to enhance audition and hide in relatively deep fresh water for prey.

In many species of invertebrates [14] or vertebrates [15], the choice of low-risk feeding seems to be evaluated as a cost-benefit analysis influenced by hunger cues that face immediate risks, including nociception, that may lead to identification by the host. To prevent detection by hosts throughout the bloodsucking process, leeches performed 3 crucial operations: inhibition of blood coagulation, suppression of inflammation, and pain relief. Hyaluronidase (*LHYAL*) boosts the diffusion and penetration of bioactive substances into tissues, and it can be used to ameliorate various complications associated with hyaluronic acid [16]. Hirudin (*HIRM1*) not only prevents fibrinogen clotting but also hinders other thrombin-catalyzed hemostatic reactions and activation of thrombin-induced platelets [17]. Additionally, hirudin can dissolve clots that have been already formed by promoting the release of tissue-plasminogen activator (T-PA) [18], so it may help in thrombus clearance. Antistasin (*ANTA*) can inhibit the function of coagulation factor Xa [19], and protein S (*PROS1*) blocks anticoagulant protease coenzyme C and factor VIII [20]. Moreover, throughout the bloodsucking process, leeches can activate anti-inflammatory proteins that may lower pain sensitivity and avoid host detection. Similarly, genes related to sanguivorous behavior such as *GLB3* and *FBN1* are essential for bloodsucking leeches. *GLB3*, which is associated with oxygen binding and carrier, heme, and iron ion binding, is involved in the formation of the hemoglobin complex [21], whereas *FBN1*, which is a major structural component of microfibrils, has been found to be the largest influential factor for height-associated variation in a human population [22]. In this study, we found that the copy number of genes related to sanguivorous behaviors was higher in the bloodsucking leeches than in the nonbloodsucking leeches. Furthermore, the expressions of these genes responded dynamically to the bloodsucking process.

Overall, we have provided 3 leech genomes with optimal assemblies and raised some profoundly interesting questions on the environmental perception and sanguivorous behaviors of leeches. The chromosome-level reference genomes and underlying genetic mechanisms may provide insights into the genetic basis of the bloodsucking lifestyle of leeches. The comprehensive genomic and transcriptomic datasets may serve as a powerful platform to facilitate innovations in the artificial culture and strain optimization of leeches, identification of novel bioactive compounds, and candidate drug prospecting.

## Methods

### DNA isolation, Nanopore library preparation, and sequencing

Three leech species—namely, *H. nipponia, H. manillensis*, and *W. pigra—*were obtained from the bank of Changjiang River, and their intestinal tracts were removed and washed with saline solution. The genomic DNA was collected using the DNeasy Blood & Tissue Kit (Qiagen, Wroclaw, Poland). The DNA quality was assessed, a long-read library was constructed (insert size, 20 kb), and a Nanopore PromethION platform was used to perform long-read sequencing. Hi-C was performed using the following protocol: the leech tissues were fixed in 1% formaldehyde solution. Nuclear chromatin was obtained from the fixed tissue and digested using *Hind*III (New England Biolabs [NEB], Ipswich, MA, USA). The overhangs were blunted with bio-14-dCTP (Invitrogen, Carlsbad, CA, USA) and Klenow enzyme (NEB). After dilution and religation using T4 DNA ligase (NEB), the genomic DNA was extracted and sheared to 350–500 bp with a Bioruptor (Diagenode, Seraing, Belgium). Then, the biotin-labeled DNA fragments were enriched with streptavidin beads (Invitrogen).

### Genome size estimation

The genome size was estimated using 17-mer analysis. The short reads were mapped to the genomes of bacteria and leeches by using Minimap2 v2.17-r941 (RRID:SCR_018550) [23]. The reads that aligned best to the bacterial genomes were filtered. Fastp v0.20.0 (RRID:SCR_016962) [24] was used to filter the low-quality reads. Jellyfish v2.3.0 (RRID:SCR_005491) [25] was used to divide the short reads into 17-mers and calculate 17-mer frequency. The 17-mer distributions of the 3 leeches generated using GenomeScope (RRID:SCR_017014) [26] followed Poisson distribution. The genome sizes were estimated by dividing the total number of 17-mers by the peak of the distribution and found to be 206 Mb, 155 Mb, and 172 Mb for *H. nipponia, H. manillensis*, and *W. pigra*, respectively.

### Genome assembly and assessment

Nanopore long reads (∼43 Gb for *H. nipponia*, ∼48 Gb for *H. manillensis*, and ∼47 Gb for *W. pigra*) were used to establish *de novo* genome assemblies by using Flye v2.6 (RRID:SCR_017016) [27]. Three rounds of correction were conducted using Racon v1.4.7 (RRID:SCR_017642) [28] with the default parameters based on alignments of long reads by using Minimap2 v2.17-r941 (RRID:SCR_018550) [23]. The resulting assemblies were further polished using 2 rounds of Pilon v1.23 (RRID:SCR_014731) [29]. Contigs that covered more than 50% of the bacterial genome sequences were filtered. Finally, 3-dimensional DNA (RRID:SCR_017227) [30] was used to hierarchically cluster the contigs and obtain pseudo-chromosome assemblies. The completeness and accuracy of the final assemblies were estimated using BUSCO v5.3.2 (RRID:SCR_015008) [31], Merqury (v1.3) [8], and short-read alignment.

### Repeat annotation

Both *de novo* and homology approaches were used to identify repetitive sequences in the leech genomes. RepeatModeler v1.0.11 (RRID:SCR_015027) [32] was used to construct the *de novo* libraries. Then, RepeatMasker (RRID:SCR_012954) [32] was run for the 3 leech genomes by using the *de novo* libraries and a known repeat library (Repbase-20,181,026, RRID:SCR_021169). A total of 25–33% repeat content was obtained by combining the annotation results of the 2 approaches.

### Gene and functional annotation

Three gene prediction methods based on *de novo* prediction, homologous genes, and transcriptomes were used to annotate protein-coding genes in the 3 leech genomes. Two *de novo* programs, Augustus v3.0.3 (RRID:SCR_008417) [33] and SNAP v2006-07-28 (RRID:SCR_007936) [34], were used to predict genes in the repeat-masked genome sequences. Transcriptome assemblies processed with PASA r20140417 (RRID:SCR_014656) [35] were used to train gene model parameters for the 2 *de novo*programs. For homology-based prediction, protein sequences from *Capitella teleta, H. robusta*, and *Eisenia andrei* were aligned over the leech genomes by using tblastn (e-value <10^–5^). GenblastA (RRID:SCR_020951) [36] was used to cluster adjacent high-scoring pairs from the same protein alignments, and GeneWise v2.4.1 (RRID:SCR_015054) [37] was used to identify accurate gene structures. After quality control and filtering, reads from all RNA libraries were mapped to the leech genomes by using hisat2 v2.1.0 (RRID:SCR_015530) [38], and StringTie v2.0.6 (RRID:SCR_016323) [39] was subsequently used to predict the gene models. All predicted genes from the 3 approaches were combined with EVM r2012-06-25 (RRID:SCR_014659) [40] to generate high-confidence gene sets.

To obtain gene function annotations, SwissProt and TrEMBL [41] protein databases were searched using blastp (RRID:SCR_001010) (e-value <1e-05). The best blastp hits were used to assign homology-based gene functions. KOBAS v3.0.3 (RRID:SCR_006350) [42] was used to search the KEGG [43] database for KO assignments. The functional classification of GO categories and InterPro entries was performed using InterProScan v5.39–77.0 (RRID:SCR_005829) [44].

### Annotation of ncRNAs

RNAmmer v1.2 (RRID:SCR_017075) [45] was used to identify the ribosomal RNA genes. tRNAscan-SE v.2.0.5 (RRID:SCR_010835) [46] was used to annotate the transfer RNA (tRNA) genes, and tRNAs decoding 20 standard amino acids were reserved. Other noncoding RNAs, including microRNAs and small nuclear RNAs, were detected using Infernal v1.1.2 (RRID:SCR_011809) [47]. All programs were run with default parameters.

### Prediction of secreted proteins

For the secreted protein analysis, 3 methods—SignalP 5.0 (RRID:SCR_015644) [48], Phobius (RRID:SCR_015643) [49], and SPOCTOPUS [50]—were used. SignalP 5.0 (RRID:SCR_015644) focuses on the prediction of signal peptides (SPs), and the other 2 algorithms can predict both transmembrane regions and SPs. The protein with at least 1 SP predicted using at least 2 of the 3 methods was identified as a secreted protein.

### Gene family construction

The following 14 species were compared to construct the gene families: *Amphimedon queenslandica, Anopheles gambiae, C. teleta, Cimex lectularius, Desmodus rotundus, H. robusta, Ixodes scapularis, Lottia gigantea, Petromyzon marinus, Rhodnius prolixus, E. andrei, H. manillensis, W. pigra*, and *H. nipponia*. The longest transcript for each gene was selected, and OrthoFinder v2.3.3 (RRID:SCR_017118) [51] software was used to cluster the gene families on the basis of the all-versus-all blastp alignments. Expansion and contraction of the gene families were detected using CAFÉ v4.2.1 (RRID:SCR_018924) [52].

### Phylogenetic tree and divergence time

To perform phylogenetic analyses, peptide alignments for each single-copy family were obtained using MUSCLE (RRID:SCR_011812) [53] and concatenated to a supergene for each species. RAxML v8.2.9 (RRID:SCR_006086) [54] with PROTGAMMAAUTO model and 100 bootstraps was used to construct the phylogenetic tree. The peptide alignments were converted to CDS sequences, which were analyzed using mcmctree in the PAML v4.9 (RRID:SCR_014932) [55] package to estimate divergence time.

### Syntenic analysis

MCScanX (RRID:SCR_022067) [56] with default parameters was used to detect syntenic genome regions among the 3 leeches, and jcvi (RRID:SCR_021641) was used to plot Fig. 1B and show their syntenic relationships.

### Transcriptome analysis

The total RNA was extracted from different leech parts at different developmental stages and before/after bloodsucking (each sample included 3 biological replicates) by using TRIzol reagent (Invitrogen). RNA purification was performed using the RNeasy Mini Kit (Qiagen, Chatsworth, CA, USA). Sequencing libraries were generated using the NEBNext Ultra RNA Library Prep Kit for Illumina (San Diego, CA, USA), according to the manufacturer's instructions. The libraries were sequenced on an Illumina HiSeq 4000 platform, and 150-bp paired-end reads were generated. Each sample was trimmed using Trimmomatic v.0.39 (RRID:SCR_011848) [57] with the options “ILLUMINACLIP: TruSeq2-PE.fa:2:30:10 SLIDINGWINDOW:15:30 MINLEN:110 TRAILING:30 AVGQUAL:30.” After quality control, HISAT2 v 2.1.0 (RRID:SCR_015530) [37] was used to map the reads of each sample to the reference genome, and SAMtools v.1.9 (RRID:SCR_002105) [58] was used to sort and convert the SAM files to BAM. Then, StringTie v2.0.6 (RRID:SCR_016323) [38] was used to assemble and merge the transcripts of each sample. Gffcompare (v0.11.5) [59] was used to compare the merged transcripts with the reference annotation file in GTF, and StringTie v2.0.6 (RRID:SCR_016323) was used to estimate transcript abundances with the options “-e -B -p 20.” The abundance results were folders that ended with “.balltown,” and prepDE.py was used to compare the folders. DESeq2 (RRID:SCR_015687) [60] with default parameters was used for the analysis of DEGs. To perform differential expression analysis using a genome model, the complementary DNA reads were mapped against the genome assembly by using HISAT2 (RRID:SCR_015530). HTSeq (RRID:SCR_005514) [61] was used to count the number of reads mapped against the annotated genes.

## Additional Files

**Supplementary Fig. S1**. The 17-mer distributions of 3 leech genomes.

**Supplementary Fig. S2**. Interspecific synteny analysis of 3 leech genomes.

**Supplementary Fig. S3**. TE sequence divergences of 4 leech genomes.

**Supplementary Fig. S4**. The number of endogenous viral elements (EVEs) in the genomes of humans and several hematophagous species.

**Supplementary Fig. S5**. Gene expression in different body parts of *H. manillensis*.

**Supplementary Fig. S6**. Gene expression in different body parts of *H. nipponia*.

**Supplementary Fig. S7**. Gene expression in different body parts of *W. pigra*.

**Supplementary Fig. S8**. Gene expression in different developmental stages of *H. manillensis*.

**Supplementary Fig. S9**. Gene expression in different developmental stages of *H. nipponia*.

**Supplementary Fig. S10**. Expression of genes related to prey location in 3 leech species.

**Supplementary Fig. S11**. Analysis of hirudin genes in 3 leech species.

**Supplementary Fig. S12**. Expression of bloodsucking-related genes in *H. manillensis*.

**Supplementary Fig. S13**. *GLB3* copies in 4 leech genomes.

**Supplementary Table S1**. Comparison of the assembled genomes with other published leech genomes.

**Supplementary Table S2**. Assessment of genome completeness and base accuracy on the basis of Illumina reads.

**Supplementary Table S3**. Mapping ratio of 2 random transcriptome assemblies for each of the 3 leech species.

**Supplementary Table S4**. BUSCO evaluation of the draft assemblies by using the metazoa_odb9 (2016–02-13) database.

**Supplementary Table S5**. Summary of the repeat contents in 3 leech genomes.

**Supplementary Table S6**. Statistics of predicted protein-coding genes in *H. nipponia* genome.

**Supplementary Table S7**. Statistics of predicted protein-coding genes in *H. manillensis* genome.

**Supplementary Table S8**. Statistics of predicted protein-coding genes in *W. pigra* genome.

**Supplementary Table S9**. Statistics of gene functional annotation of leech genomes.

**Supplementary Table S10**. Statistics of ncRNA annotation of *H. nipponia* genome.

**Supplementary Table S11**. Statistics of ncRNA annotation of *H. manillensis* genome.

**Supplementary Table S12**. Statistics of ncRNA annotation of *W. pigra* genome.

**Supplementary Table S13**. Statistics of secreted protein in leech genomes.

**Supplementary Table S14**. Copy number of genes related to prey tracking and location.

**Supplementary Table S15**. Copy number of genes related to bloodsucking characteristics.

giad023_GIGA-D-22-00200_Original_Submission

giad023_GIGA-D-22-00200_Revision_1

giad023_Response_to_Reviewer_Comments_Original_Submission

giad023_Reviewer_1_Report_Original_Submission Chao Bian -- 8/25/2022 Reviewed

giad023_Reviewer_1_Report_Revision_1 Chao Bian -- 12/15/2022 Reviewed

giad023_Reviewer_2_Report_Original_Submission zichao liu -- 9/19/2022 Reviewed

giad023_Supplemental_File

## Abbreviations

bp: base pair; BUSCO: Benchmarking Universal Single-Copy Orthologs; DEG: differentially expressed gene; GO: Gene Ontology; KEGG: Kyoto Encyclopedia of Genes and Genomes; Mb: megabase pairs; NCBI: The National Center for Biotechnology Information; ncRNA: noncoding RNA; PDE: phosphodiesterase; SP: signal peptide; tRNA: transfer RNA.

## Authors’ Contributions

J.Z. and Q.L. developed the concept of this study; Q.L., J.R., and Z.L. designed the research; X.W. performed genome assembly, gene annotation, and evolutional analysis; T.F. analyzed the transcriptome data; H.S. analyzed the bloodsucking characteristic; X.Y. analyzed the mechanical and visual characteristics; W.Z., C.Q., X.M. J.L., L.L., and K.C. helped with the sample collection; Q.L., Z.L., J.Y., W.C., and J.Y. discussed the results and implications; X.M., L.L., W.X., and J.L. helped with the medicinal applications; X.L. and H.L. performed the statistical analysis; Z.L. drafted the manuscript; S.R. revised the manuscript. All authors read and approved the final manuscript.

## Competing Interests

The authors declare no competing interests.

## Data Availability

The genomic and transcriptomic Illumina data, Nanopore sequencing, and HiC data were uploaded at NCBI with BioProject (number: PRJNA762643). The genomes and gene annotations of the 3 leeches are also shared via Figshare [62]. Supporting data are deposited in the *GigaScience* database GigaDB [63] split into the 3 species: *Whitmania pigra* [64], *Hirudo nipponia* [65], and *Hirudinaria manillensis* [66].
