# Correction to: molecular mechanisms underlying hematophagia revealed by comparative analyses of leech genomes

**DOI:** 10.1093/gigascience/giad040

**Published:** 2023-05-22

This is a correction to: Jinghui Zheng, Xiaobo Wang, Tong Feng, Saif ur Rehman, Xiuying Yan, Huiquan Shan, Xiaocong Ma, Weiguan Zhou, Wenhua Xu, Liying Lu, Jiasheng Liu, Xier Luo, Kuiqing Cui, Chaobin Qin, Weihua Chen, Jun Yu, Zhipeng Li, Jue Ruan, Qingyou Liu, Molecular mechanisms underlying hematophagia revealed by comparative analyses of leech genomes, *GigaScience*, Volume 12, 2023, giad023, https://doi.org/10.1093/gigascience/giad023.

In the originally published version of this manuscript, there were errors in Fig. 3.

Extraneous “Count” legends from sections D and E are removed and the Figure should read:

**Figure:**
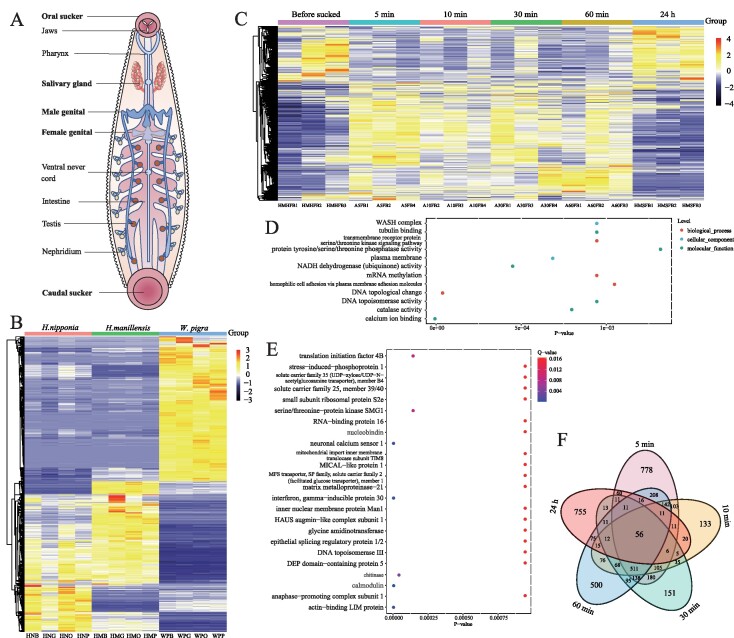


instead of:

**Figure:**
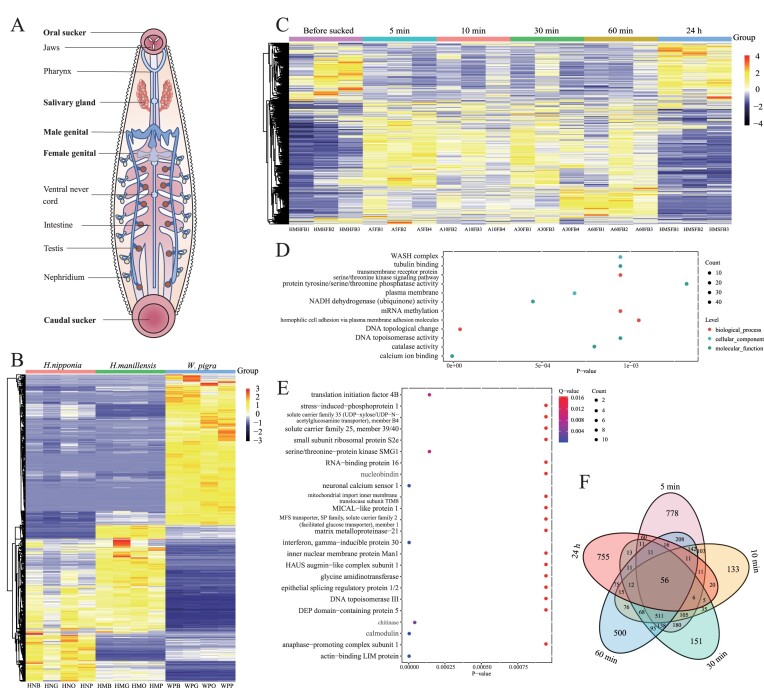


There were also emendations needed in the affiliations list and enumeration in authorlist. These should read:

"Jinghui Zheng^1,2,†^, Xiaobo Wang^3,†^, Tong Feng^3,5,†^, Saif ur Rehman^3^, Xiuying Yan^3^, Huiquan Shan^3^, Xiaocong Ma^2^, Weiguan Zhou^6^, Wenhua Xu^2^, Liying Lu^2^, Jiasheng Liu^2^, Xier Luo^1,4^, Kuiqing Cui^1,3^, Chaobin Qin^3^,Weihua Chen^5^, Jun Yu^6^, Zhipeng Li^3^, Jue Ruan^4,*^ and Qingyou Liu^1,3,*^

^1^Guangdong Provincial Key Laboratory of Animal Molecular Design and Precise Breeding, School of Life Science and Engineering, Foshan University, Foshan 528,225, China

^2^Department of Cardiology, Ruikang Hospital Affiliated to Guangxi University of Chinese Medicine, Nanning 530,011, China

^3^State Key Laboratory for Conservation and Utilization of Subtropical Agro-bioresources, Guangxi University, Nanning 530,004, China

^4^Genome Analysis Laboratory of the Ministry of Agriculture, Agricultural Genomics Institute, Chinese Academy of Agricultural Sciences, Shenzhen, Guangdong 518,120, China

^5^Department of Bioinformatics and Systems Biology, College of Life Science and Technology, Huazhong University of Science and Technology,Wuhan, Hubei 430,074, China

^6^Biological Institute of Guangxi Academy of Sciences, Nanning 530,007, China

^7^CAS Key Laboratory of Genome Sciences and Information, Beijing Institute of Genomics, Chinese Academy of Sciences, Beijing 100,101, China"

instead of:

"Jinghui Zheng^1,†^, Xiaobo Wang^2,3,†^, Tong Feng^2,3,†^, Saif ur Rehman^2^, Xiuying Yan^2^, Huiquan Shan^2^, Xiaocong Ma^1^, Weiguan Zhou^4^, Wenhua Xu^1^, Liying Lu^1^, Jiasheng Liu^1^, Xier Luo^2,3^, Kuiqing Cui^2^, Chaobin Qin^2^, Weihua Chen^5^, Jun Yu^6^, Zhipeng Li^2^, Jue Ruan^3,*^ and Qingyou Liu^7,*^

^1^Department of Cardiology, Ruikang Hospital Affiliated to Guangxi University of Chinese Medicine, Nanning 530,011, China

^2^State Key Laboratory for Conservation and Utilization of Subtropical Agro-bioresources, Guangxi University, Nanning 530,004, China

^3^Genome Analysis Laboratory of the Ministry of Agriculture, Agricultural Genomics Institute, Chinese Academy of Agricultural Sciences, Shenzhen, Guangdong 518,120, China

^4^Biological Institute of Guangxi Academy of Sciences, Nanning 530,007, China

^5^Department of Bioinformatics and Systems Biology, College of Life Science and Technology, Huazhong University of Science and Technology, Wuhan, Hubei 430,074, China

^6^CAS Key Laboratory of Genome Sciences and Information, Beijing Institute of Genomics, Chinese Academy of Sciences, Beijing 100,101, China

^7^Guangdong Provincial Key Laboratory of Animal Molecular Design and Precise Breeding, School of Life Science and Engineering, Foshan University, Foshan 528,225, China".

These changes have been made in the article.

