# Supplementary material for: Molecular mechanisms underlying hematophagia revealed by comparative analyses of leech genomes
Source: Gigascience. 2023 Apr 11;12:giad023. doi: 10.1093/gigascience/giad023 (PMC10087013; doi:10.1093/gigascience/giad023)
Supplement: giad023_GIGA-D-22-00200_Original_Submission [file giad023_giga-d-22-00200_original_submission.pdf]

GigaScience

Molecular mechanisms underlying hematophagia revealed by comparative analyses of leech genomes

--Manuscript Draft--

**Manuscript Number:**	GIGA-D-22-00200
**Full Title:**	Molecular mechanisms underlying hematophagia revealed by comparative analyses of leech genomes
**Article Type:**	Research
**Funding Information:**	National Natural Science Foundation of China (U20A2051) — Not applicable<br>National Natural Science Foundation of China (31760648) — Prof Qingyou Liu<br>National Natural Science Foundation of China (31860638) — Prof Qingyou Liu<br>Guangxi Natural Science Foundation (AB18221120) — Prof Qingyou Liu<br>Guangxi Distinguished scholars Program (201835) — Prof Qingyou Liu<br>Qihuang High-level Talent Team Training Projects of Guangxi University of Chinese Medicine: Application of Systems Biology in Chinese Medicine Research (2021005) — Prof Qingyou Liu
**Abstract:**	Leeches have been used in traditional Chinese medicine since pre-historic times to treat a spectrum of ailments but very little is known about their physiological, genetic, and evolutionary standpoint. Here we sequenced and assembled chromosome-level genome assemblies of three leech species (bloodsucking Hirudo nipponia and Hirudinaria manillensis , and non-bloodsucking Whitmania pigra) and both bloodsucking leeches have similar dynamic population history and genome-wide expression patterns compared to non-bloodsucking leech. Combined analysis of the genomic and transcriptional data revealed that bloodsucking leeches presumably enhanced auditory other than visual sense for prey location in relatively deep fresh water. As expected, the copy number of genes related to anticoagulation, analgesia, and anti-inflammation obviously increased in the bloodsucking leeches, and their dynamic gene expressions respond to the bloodsucking process. We also found that the expanded FBN1 gene family might help for leech body rapid swelling after bloodsucking, and the expanded GLB3 gene family was potentially used to store prey blood for a long time in the leech's body.
**Corresponding Author:**	Qingyou Liu<br>Foshan University<br>Foshan, GaungDong CHINA
**Corresponding Author Secondary Information:**	
**Corresponding Author's Institution:**	Foshan University
**Corresponding Author's Secondary Institution:**	
**First Author:**	Jinghui Zheng
**First Author Secondary Information:**	
**Order of Authors:**	Jinghui Zheng
	Xiaobo Wang
	Tong Feng

Powered by Editorial Manager® and ProduXion Manager® from Aries Systems Corporation

	Saif ur Rehman
	Xiuying Yan
	Huiquan Shan
	Xiaocong Ma
	Weiguan Zhou
	Wenhua Xu
	Liying Lu
	Jiasheng Liu
	Xier Luo
	Kuiqing Cui
	Chaobin Qin
	Weihua Chen
	Jun Yu
	Zhipeng Li
	Jue Ruan
	Qingyou Liu
**Order of Authors Secondary Information:**	
**Additional Information:**	
**Question**	**Response**
Are you submitting this manuscript to a special series or article collection?	No
**Experimental design and statistics**<br><br>Full details of the experimental design and statistical methods used should be given in the Methods section, as detailed in our Minimum Standards Reporting Checklist. Information essential to interpreting the data presented should be made available in the figure legends.<br><br>Have you included all the information requested in your manuscript?	Yes
**Resources**<br><br>A description of all resources used, including antibodies, cell lines, animals and software tools, with enough information to allow them to be uniquely identified, should be included in the Methods section. Authors are strongly	Yes

Powered by Editorial Manager® and ProduXion Manager® from Aries Systems Corporation

encouraged to cite Research Resource Identifiers (RRIDs) for antibodies, model organisms and tools, where possible.<br><br>Have you included the information requested as detailed in our Minimum Standards Reporting Checklist?	
**Availability of data and materials**<br><br>All datasets and code on which the conclusions of the paper rely must be either included in your submission or deposited in publicly available repositories (where available and ethically appropriate), referencing such data using a unique identifier in the references and in the “Availability of Data and Materials” section of your manuscript.<br><br>Have you have met the above requirement as detailed in our Minimum Standards Reporting Checklist?	Yes

Powered by Editorial Manager® and ProduXion Manager® from Aries Systems Corporation

Click here to access/download;Manuscript;Manusript.docx

Molecular mechanisms underlying hematophagia revealed by comparative analyses of leech genomes

Jinghui Zheng*[2], Xiaobo Wang*[3,4], Tong Feng*[3,4], Saif ur Rehman[3], Xiuying Yan[3], Huiquan Shan[3], Xiaocong Ma[2], Weiguan Zhou[6], Wenhua Xu[2], Liying Lu[2], Jiasheng Liu[2], Xier Luo[3,4], Kuiqing Cui[3], Chaobin Qin[3], Weihua Chen[5], Jun Yu[7], Zhipeng Li[3], Jue Ruan[†4], Qingyou Liu[†1]

1. Guangdong Provincial Key Laboratory of Animal Molecular Design and Precise Breeding, School of Life Science and Engineering, Foshan University, 528225, Foshan, China
2. Department of Cardiology, Ruikang Hospital Affiliated to Guangxi University of Chinese Medicine, Nanning 530011, China.
3. State Key Laboratory for Conservation and Utilization of Subtropical Agro- bioresources, Guangxi University, Nanning 530004, China
4. Genome Analysis Laboratory of the Ministry of Agriculture, Agricultural Genomics Institute, Chinese Academy of Agricultural Sciences, Shenzhen, Guangdong, China.
5. Department of Bioinformatics and Systems Biology, College of Life Science and Technology, Huazhong University of Science and Technology, Wuhan, Hubei, China.
6. Biological Institute of Guangxi Academy of Sciences, Nanning 530007, China.
7. CAS Key Laboratory of Genome Sciences and Information, Beijing Institute of Genomics, Chinese Academy of Sciences, Beijing 100101, China.

*These authors contributed equally: Jinghui Zheng, Xiaobo Wang and Tong Feng

†Corresponding author E-mail: qyliu-gene@gxu.edu.cn; ruanjue@caas.cn

Abstract

Leeches have been used in traditional Chinese medicine since pre-historic times to treat a spectrum of ailments but very little is known about their physiological, genetic, and evolutionary standpoint. Here we sequenced and assembled chromosome-level genome assemblies of three leech species (bloodsucking *Hirudo nipponia* and *Hirudinaria manillensis*, and non-bloodsucking *Whitmania pigra*) and both bloodsucking leeches have similar dynamic population history and genome-wide expression patterns compared to non-bloodsucking leech. Combined analysis of the genomic and transcriptional data revealed that bloodsucking leeches presumably enhanced auditory other than visual sense for prey location in relatively deep fresh water. As expected, the copy number of genes related to anticoagulation, analgesia, and anti-inflammation obviously increased in the bloodsucking leeches, and their dynamic gene expressions respond to the bloodsucking process. We also found that the expanded *FBN1* gene family might help for leech body rapid swelling after bloodsucking, and the expanded *GLB3* gene family was potentially used to store prey blood for a long time in the leech's body.

Introduction

Leeches are obligate blood-feeding arthropods distributed from tropical to subarctic regions around the globe. Hematophagous species, such as bats, ticks, and mosquitos are the most versatile vectors capable of transmitting a wide range of pathogens to humans, livestock, and wildlife, including protozoa, bacteria, nematodes, fungi, and viruses [1], whereas leeches have been found to transmit a few infectious diseases. Furthermore, leeches have been used in traditional Chinese medicine since pre-historic times to treat a spectrum of ailments. Most obviously, leeches secrete the most potent natural thrombin inhibitor hirudin [2] and exhibited a variety of fascinating behavioral and physiological characteristics that are of interest to evolutionary, biochemical, and pharmaceutical studies. Leeches have also developed persistent adaptive strategies and characteristics to perceive their environment during long-term evolution. Leeches continuously receive sensory information from their surroundings either by mechanical or visual sensation to locate and target their prey. Additionally, the sanguivorous behaviour of leeches is capable to reduce natural host reflexes (blood coagulation, pain and inflammation) during bloodsucking [3]. Meanwhile, the prey localization, sanguivorous behaviour and medicinal value of leeches, therefore, necessitate to explore the fundamental knowledge of leech genomes and genetic diversity which would undoubtedly open new avenues for research on leech biology, host interactions, and control strategies at the molecular level which have not yet been elucidated. Heretofore, leech research was primarily focused on strain optimization, artificial culturing, and the identification and development of therapeutic strategies however, well annotated genome or genetic data is still unavailable. Currently, a few leech species genomic data including one non-blood sucking leech (*Helobdella robusta*) and low coverage genome sequence data of two lineages *Amynthas cortices* have been published [3-6], but none of them reach to chromosome level. Here we provided three high-quality leech genomes and abundant transcriptomes which illustrated the gene expression dynamics of bloodsucking leeches including anticoagulation, analgesic, and anti-inflammation that would facilitate the understanding at the genetic level and could be crucial for drug candidate prospecting.

Results

Genome assemblies

We sequenced with the Nanopore platform and performed genome assembly for three leech species, including *Hirudo nipponia*, *Hirudinaria manillensis*, and *Whitmania pigra* (Fig. 1A), which are ubiquitously used in the Chinese pharmacopeia. The assemblies contained 985, 622, and 437 Mb contigs with N50 contig lengths of 1.1,

2.5, and 4.1 Mb for *H. nipponia*, *H. manillensis*, and *W. pigra*, respectively (Table 1). Based on HiC data, the contigs were further consolidated into scaffolds having N50 lengths of 18.5, 11.9, and 16.2 Mb, respectively, comprising 11, 13, and 11 pseudo-chromosomes, for *H. nipponia*, *H. manillensis* and *W. pigra* (Table 1 and Fig. 1B). The size of the final assembled genome assemblies corresponding to *H. nipponia*, *H. manillensis* and *W. pigra* were approximately 203.7, 157.5, and 181.4 Mb, that were similar to the estimated genome sizes based on k-mer (K = 17) analysis (Supplementary Fig. 1). The assemblies presented larger scaffold N50 sizes and lower scaffold numbers, indicating higher continuity than previously reported genomes (Supplementary Table 1).

Furthermore, we aligned the short reads and transcriptome assemblies to the genome to assess the completeness of our genome assemblies and found that more than 98% of the reads and 95% of transcriptome data were mapped to the assemblies (Supplementary Table 2 and 3). We also estimated the completeness and accuracy of the final assemblies and found that 91.5%, 90.8%, and 91.7% of the BUSCO orthologs were captured, respectively (Supplementary Table 4). Using Merqury [7], we obtained the QV scores of 35.8 (for *W. pigra*), 33.4 (for *H. nipponia*) and 32.1 (for *H. manillensis*). By combining the results of the de novo and homolog-based approaches, about 25 – 33% of repetitive sequences in the leech genomes were identified (Supplementary Table 5 and Supplementary Fig. 3).

Population history of leeches

We used the pairwise sequentially Markovian coalescent (PSMC) analysis to infer changes in the effective population size (Ne) of the ancestral populations of leeches. The population of the non-bloodsucking leech *W. pigra* underwent two expansions while the populations of the two bloodsucking leeches including *H. nipponia* and *H. manillensis* experienced only one expansion (Fig. 1C). The different fluctuations of Ne might hint at different enviromental adaptations of the two type of leeches. Interestingly, the Ne of the bloodsucking leeches begins to decrease at the onset of Pleistocene (~2 Mya), which is characterized by repeated cycles of glaciations. The glaciations likely have reduced the contact between leeches and animal hosts, resulting in a decline in the size of bloodsucking leech populations.

Gene annotation and gene family construction

Three methods including de novo, homology-based and transcriptome-based gene predictions were used and we identified a total of 20430, 18106, and 18540 protein-coding genes in the *H. nipponia*, *H. manillensis* and *W. pigra* genomes, where the total CDS lengths were 32.34, 27.41 and 32.29 Mb, and mean CDS lengths were 1739, 1647 and 1801 bp, respectively (Supplementary Tables 6 – 8). Further, the gene sets were aligned against the Uniprot, Interpro and KEGG database, and about 88% of the genes were functionally assigned or annotated (Supplementary Table 9). Besides,

thousands of ncRNA genes and secreted genes were also identified in each of the three leech genomes (Supplementary Tables 10 – 13).

Meanwhile, we constructed the gene family and performed a phylogenetic analysis for 14 species. *W. pigra* and *H. nipponia* shared a common ancestor about 50 million years ago, whereas *H. manillensis* diverged at an earlier date (Fig. 2A). It implied that the blood-sucking behavior might have existed in the ancestors of leeches, but this behavior was lost in the lineage of *W. pigra*. Consistent with evolutionary relationships, the four leech species were found to share most of their gene families (Fig. 2B). Additionally, a total of 1,289, 925 and 719 expanded, and 927, 2,164 and 1,312 contracted gene families have also been identified for each of *H. nipponia*, *H. manillensis* and *W. pigra* genomes. The GO analysis depicted that the expanded gene families including the ATP-binding cassette transporter complex, transcription factor IIA complex and calcium ion binding functions were significantly enriched in both bloodsucking leech species (Fig. 2C).

Transcriptome dynamics

To explore the gene expression patterns of the three leeches, we sequenced and analyzed the transcriptome of 32 samples (three replicates for each sample) including different developmental stages, tissues, and a series of bloodsucking behavior at 5 different time points (Supplementary Fig. 5-9). We used DEseq2 to identify differentially expressed genes (DEGs) between bloodsucking leeches and non-bloodsucking leech, and found that the majority of DEGs of two bloodsucking leeches shared similar expression patterns (Fig. 3B). We further investigated the transcriptomic dynamics during the process of bloodsucking in *H. manillensis*. Mostly the DEGs respond quickly after bloodsucking and continuously changed in the following 60 minutes (Fig. 3C, F). After 24 hours, the expression patterns of these DEGs had virtually restored to that of the pre-bloodsucking group (Fig. 3C). Furthermore, the GO and KEGG pathway analyses identified that most of the DEGs were significantly enriched to calcium, indicating that calcium-related regulation might play an important part in bloodsucking behavior of leeches (Fig. 3D, E).

Genetic basis of the prey location for leeches

Leeches are efficient predators because they can utilize their mechanical and auditory systems to acquire information and locate their prey. The genetic diversity of specific phenotypic traits could impersonate a better prototype of biologically substantial diverse genes especially having a crucial role in the development of the auditory and visual systems in mammals and in leeches these were identified by homolog-based functional annotation (Fig. 4). In leeches, among hearing-related genes (Fig. 4A), *SIX1* plays a crucial role for audio sensation and we found that there is a single copy of *SIX1* gene in the non-bloodsucking leech, while two or four copies of *SIX1* gene are present in the bloodsucking leech (Supplementary Table 14) and the expression pattern of *SIX1* gene was generally higher in bloodsucking than non-bloodsucking

leech (Supplementary Fig. 10) which clearly indicated that bloodsucking leech might possess better auditory perception.

Similarly, various mechanisms go on in the eye for the detection of visual signals, with the coordinated involvement of genes and their related proteins or enzymes, and intriguingly, the gene *PDE6D*, encoding the delta subunit of rod-specific photoreceptor phosphodiesterase, existed in non-bloodsucking leech while lost in the bloodsucking leech (Supplementary Table 14). Thus, we speculated that bloodsucking leech potentially strengthened audition and weakened vision to hide in the relatively deep fresh water.

Genetic basis of the sanguivorous behavior of bloodsucking leeches

To avoid being detected by hosts during the bloodsucking process, leeches executed three key operations including inhibition of blood coagulation, suppression of inflammation and alleviating pain. We identified the related genes for each process (Fig. 5) and anticipatedly found that the total copy number of these genes in bloodsucking leech was higher than non-bloodsucking leech (Supplementary Table 14).

We found that two hyaluronidase (*LHYAL*) copies reached their highest expressions in only 5 minutes during the bloodsucking process (Supplementary Fig. 11). Leeches inhibited hemagglutination mainly via three ways: supressing the thrombin cascade (*HIRM1*, *ANTA* and *PROS1*), inhibiting platelet aggregation (*DECO*, *MMP13*, *ADAMTS18*, *APY* and *HIRM1*) and dilating vessels (*NEP1* and *HIRM1*) (Fig. 5A). In this study, *HIRM1* mainly express in the oral suckers of the three leech species and the expression of one *HIRM1* copy was comparatively increased at the time point of 30 minutes (Supplementary Fig 11), which was consistent with the time of physiological coagulation. As expected, the gene expressions of five antistasin (*ANTA*) copies and two *PROS1* copies retained relatively higher during the process of bloodsucking (5-60 minutes) (Supplementary Fig. 11). Besides, all gene copies of *ADAMTS18* and *NEP1* acquired their peak expression levels in 10 minutes, suggesting that they potentially played a pivotal role in the process of inhibiting hemagglutination.

Additionally, during the bloodsucking process leeches also produced agrin (*AGRN*), cystatin (*CYT*), neprilysin-1 (*NEP1*) and membrane metalloendopeptidase like 1 (*MMEL1*) (Fig. 5B), which may reduce pain sensitivity and help them to avoid being recognized by the host. All copies of *AGRN* and *MMEL1* as well as *CYT* generally kept high expressions in the bloodsucking process (Supplementary Fig. 11). Further analysis showed that the leeches also expressed various anti-inflammatory genes, including *NEP1*, *MMEL1*, *CYT*, eglin (*ICIC*), cystatin (*CYT*), Leuko*CYT*e elastase inhibitor (*SERPINB1*), Toll-like receptor 4 (*TLR4*) and lipoprotein receptor-related protein 1 (*LRP1*) (Fig. 5C). Surprisingly, the expression levels of most of *TLR4* and *LRP1* copies reduced quickly at the beginning of the bloodsucking process and increased after bloodsucking (24 hours) process (Supplementary Fig. 11). It indicated

that leeches always kept anti-inflammatory proteins on hand to swiftly release into the body of prey.

Moreover, we noticed that *GLB3* and *FBN1* were the most expanded genes associated with sanguivorous behaviour in the bloodsucking leech. Seven or eight copies of the *GLB3* were tandemly arranged in the two bloodsucking leeches, while only one copy in *W. pigra* and no copy in H. robusta (Supplementary Table 13 and Supplementary Fig. 12). Moreover, the expression levels of the *GLB3* family increased in the bloodsucking process. Noticeably, three *GLB3* copies displayed significant expression level changes after the bloodsucking process (Supplementary Fig. 12). Presumably that could explain why leeches can store prey blood in their body for months. Twelve copies of *FBN1* were detected in the two bloodsucking species, and only four or zero copies were found in non-bloodsucking leeches (Supplementary Table 15). In general, The *FBN1* family continually increased their expressions during the bloodsucking process (Supplementary Fig. 12), indicating that *FBN1* might be associated with the adaptability of leech body swelling after bloodsucking.

Discussion

Precise non-redundant reference genomes with vindicated annotations are critical for functional as well as evolutionary analyses and indeed, it was remained a challenge to produce a highly accurate chromosome-level assembly, particularly for leeches' chromosomes. Although leeches have been used to treat diverse ailments since ancient times, most of our information about them is based on psychometrics. A comprehensive catalog of their genome and gene expression pattern is fundamental to understanding the genetic basis of the behavior and will be crucial for drug candidate prospecting. Though the genome of medicinal leech has been sequenced in several other studies, the assembling results rest on fragmental level [3-6]. But, in this study, we developed three chromosome-level genome assemblies of the *H. nipponia*, *H. manillensis* and *W. pigra* by integrating short-read sequencing, Nanopore sequencing and Hi-C technology.

Leeches are efficient predators owning to their specialized predation adaptation, with acute senses such as hearing vision and chemosensation. Leeches trace and locate their prey via mechanical and visual cues from water waves on the basis of S cells [8]. Ethological experiments have shown that leeches can quickly identify and locate the source of sound by analyzing the distribution of water waves [8]. Among hearing-related genes, *SIX1* mediated the relative numbers of sensory hair cells and statoacoustic ganglion neurons [9]. Overexpression of the *SIX1* gene could result in more hair cells [9]. In our study higher expression of the *SIX1* gene in bloodsucking leech than non-bloodsucking leech clearly indicated that bloodsucking leech might possess better auditory perception. Furthermore, genes that encode opsin had very early origins and were recruited repeatedly during eye evolution [10]. The opsin family can be divided into seven subfamilies, and rhodopsin and Gq-coupled

opsin/melanopsin are the most abundant proteins in rod cells [11]. Phototransduction is initiated when rhodopsin absorbs photons and triggers the exchange of GDP for GTP on the G-protein, which leads to an increase in cGMP hydrolysis by the phosphodiesterase (PDE) complex [12] and surprisingly, the gene PDE6D, which encodes the delta subunit of rod-specific photoreceptor phosphodiesterase, was present in non-bloodsucking leeches but not in bloodsucking leeches. We demonstrated that bloodsucking leech possibly prefers enhancing audition to hide in the relatively deep fresh water for prey.

In many species of invertebrates [13] or vertebrates [14], the choice of feed with low risk seems to be evaluated as a cost-benefit analysis influenced by hunger cues which face immediate risks including nociception that led to be identified by the host. To prevent detection by hosts throughout the bloodsucking process, leeches performed three crucial operations: inhibition of blood coagulation, suppression of inflammation, and pain relief. Hyaluronidase (*LHYAL*) boosts the diffusion and penetration of bioactive substances into tissues, and it can be used to ameliorate various complications associated with hyaluronic acid [15]. Hirudin (*HIRM1*) not only prevents fibrinogen clotting but also hinders other thrombin-catalyzed haemostatic reactions and activation of thrombin-induced platelets [16]. Additionally, hirudin can dissolve clots that have already formed by promoting the release of T-PA [17], so it may help in the clearance of thrombus. Apart from hirudin, antistasin (*ANTA*) can inhibit the function of coagulation factor Xa [18], and protein S (*PROS1*) blocks anticoagulant protease coenzyme C and factor VIII [19]. Moreover, throughout the bloodsucking process, leeches could also activate the anti-inflammatory proteins that might lower pain sensitivity and avoid being detected by the host. Similarly, for bloodsucking leeches the genes related to sanguivorous behaviour such as *GLB3* and *FBN1* are crucial such as *GLB3* which is associated with oxygen binding and carrier, haeme and iron ion binding, is involved in the formation of the haemoglobin complex [20] whereas *FBN1* that is a major structural component of microfibrils, and was found to be the largest influential factor for a height-associated variation in a human population [21]. In this study, we found that the copy number of genes related to sanguivorous behaviours in bloodsucking leech is higher than in non-bloodsucking leech. Furthermore, the dynamic gene expression of these genes responded to the bloodsucking process.

Overall, we have provided three leech genomes with optimal assemblies and raised some profoundly interesting questions on the environmental perception and sanguivorous behaviours of leeches. The chromosome-level reference genomes and underlying genetic mechanisms possibly provide insights into the genetic basis of leeches to the bloodsucking lifestyle. The comprehensive genomic and transcriptomic datasets may serve as a powerful platform to facilitate innovations in the artificial culture and strain optimization of leeches, identification of novel bioactive compounds, and candidate drug prospecting.

Methods

DNA isolation, Nanopore library preparation and sequencing

Three leech species, namely, *Hirudo nipponia, Hirudinaria manillensis* and *Whitmania pigra*, were obtained from the bank of Changjiang river, and their intestinal tracts were removed and washed with saline solution. The genomic DNA was collected using the DNeasy Blood & Tissue Kit (Qiagen, Wroclaw, Poland). The DNA quality was assessed, a long-read library was constructed (insert size, 20 kb), and Nanopore platform was used to perform long-read sequencing. Hi-C was performed using the following protocol: The leech tissues were fixed in 1% formaldehyde solution. Nuclear chromatin was obtained from the fixed tissue and digested using HindIII (New England Biolabs, NEB, USA). The overhangs were blunted with bio-14-dCTP (Invitrogen, California, USA) and Klenow enzyme (NEB). After dilution and re-ligation using T4 DNA ligase (NEB), the genomic DNA was extracted and sheared to 350－500 bp with a Bioruptor (Diagenode, Belgium). Then, the biotin-labeled DNA fragments were enriched with streptavidin beads (Invitrogen).

Genome size estimation

The genome size was estimated using 17-mer analysis. First, short reads were mapped on the genomes of bacteria and leeches by using minimap2 (v2.17-r941) [22]. The reads that aligned best on the bacterial genomes were filtered. Fastp (v0.20.0) [23] was used to filter the low-quality reads. Jellyfish (v2.3.0) [24] was used to divide the short reads into 17-mers and calculate 17-mer frequency. The 17-mer distributions of the three leeches generated using GenomeScope [25] followed Poisson distribution. The genome sizes were estimated by dividing the total number of 17-mers by the peak of the distribution and found to be 206 Mb, 155 Mb and 172 Mb for *H. nipponia*, *H. manillensis* and *W. pigra*, respectively.

Genome assembly and assessment

Nanopore long reads (~43 Gb for *H. nipponia*, ~48 Gb for *H. manillensis* and ~47 Gb for *W. pigra*) were used to establish de novo genome assemblies by using Flye (v2.6) [26]. Three rounds of correction were conducted using Racon (v1.4.7) [27] with the default parameters based on alignments of long reads by using minimap2 (v2.17-r941) [22]. The resulting assemblies were further polished using two rounds of Pilon (v1.23) [28]. Contigs that covered more than 50% of the bacterial genome sequences were filtered. Finally, LACHESIS [29] was used to hierarchically cluster the contigs and obtained the pseudo-chromosome assemblies. The completeness and accuracy of the final assemblies were estimated using both BUSCO (v5.4.2) [30], Merqury (v1.3) [7] and short read alignment.

Repeat annotation

Both de novo and homology approaches were used to identify repetitive sequences in the leech genomes. RepeatModeler (v1.0.11) (http://www.repeatmasker.org/RepeatModeler/) was used to construct the de novo libraries. Then, RepeatMasker (http://www.repeatmasker.org/) was run for the three leech genomes by using the de novo libraries and a known repeat library (Repbase-20181026). A total of 25 – 33% repeat content was obtained by combining the annotation results of the two approaches.

Gene and functional annotation

Three gene prediction methods based on de novo prediction, homologous genes, and transcriptomes were used to annotate protein-coding genes in the three leech genomes. Two de novo programs, Augustus (v3.0.3) [31] and SNAP (v2006-07-28) [32], were used to predict genes in the repeat-masked genome sequences. Transcriptome assemblies processed with PASA (r20140417) [33] were used to train gene model parameters for the two de novo programs. For homology-based prediction, protein sequences from C. teleta, H. robusta and E. andrei were aligned over the leech genomes by using tblastn (e-value < 10-5). GenblastA [34] was used to cluster adjacent high-scoring pairs from the same protein alignments, and GeneWise (version 2.4.1) [35] was used to identify accurate gene structures. After quality control and filtering, reads from all RNA libraries were mapped to the leech genomes by using hisat2 (v2.1.0) [36], and StringTie (v2.0.6) [37] was subsequently used to predict the gene models. All predicted genes from the three approaches were combined with EVM (r2012-06-25) [38] to generate high-confidence gene sets.

To obtain gene function annotations, SwissProt and TrEMBL [39] protein databases were searched using blastp (e-value<1e-05). The best blastp hits were used to assign homology-based gene functions. KOBAS (v3.0.3) [40] was used to search the KEGG [41] database for KO assignments. The functional classification of GO categories and InterPro entries was performed using InterProScan (version 5.39-77.0) [42].

Annotation of ncRNAs

RNAmmer (v1.2) [43] was used to identify the rRNA genes. tRNAscan-SE (v.2.0.5) [44] was used to annotate the tRNA genes, and tRNAs decoding 20 standard amino acids were reserved. Other non-coding RNAs, including miRNAs and snRNAs, were detected using Infernal (v1.1.2) [45]. All programs were run with default parameters.

Prediction of secreted proteins

For the secreted protein analysis, three methods, SignalP5.0 [46], Phobius [47] and SPOCTOPUS [48], were used. SignalP5.0 focuses on the prediction of signal peptides (SPs), and the other two algorithms can predict both transmembrane regions and SPs. The protein with at least one SP predicted using at least two out of the three methods was identified as a secreted protein.

Gene family construction

The following 14 species were compared to construct the gene families: Amphimedon queenslandica, Anopheles gambiae, Capitella teleta, Cimex lectularius, Desmodus rotundus, Helobdella robusta, Ixodes scapularis, Lottia gigantea, Petromyzon marinus, Rhodnius prolixus, Eisenia andrei, *H. manillensis*, *W. pigra* and *H. nipponia*. The longest transcript for each gene was selected, and OrthoFinder (v2.3.3) [49] software was used to cluster the gene families based on the all-versus-all blastp alignments. Expansion and contraction of the gene families were detected using CAFÉ (v4.2.1) [50].

Phylogenetic tree and divergence time

To perform phylogenetic analyses, peptide alignments for each single-copy family were obtained using MUSCLE [51] and concatenated to a supergene for each species. RAxML (v8.2.9) [52] with PROTGAMMAAUTO model and 100 bootstraps was used to construct the phylogenetic tree. The peptide alignments were converted to CDS sequences, which were subjected to mcmc tree in PAML (v4.9) [53] package to estimate divergence time.

Syntenic analysis

Mcscanx [54] with default parameters was used to detect syntenic genome regions among the three leeches, and jcvi was used to plot Figure 1B and show their syntenic relationships.

Transcriptome analysis

The total RNA was extracted from different leech parts at different developmental stages and before/after bloodsucking (Note that each sample included three biological replicates) by using TRIzol reagent (Invitrogen Corp., Carlsbad, CA). RNA purification was performed using the RNeasy Mini Kit (Qiagen, Chatsworth, CA). Sequencing libraries were generated using the NEBNext Ultra RNA Library Prep Kit for Illumina (NEB, USA), according to the manufacturer's recommendations. The libraries were sequenced on an Illumina HiSeq 4000 platform, and 150 bp paired-end reads were generated. Each sample was trimmed using Trimmomatic (v.0.39) [55] with the options 'ILLUMINACLIP: TruSeq2-PE.fa:2:30:10 SLIDINGWINDOW:15:30 MINLEN:110 TRAILING:30 AVGQUAL:30'. After quality control, HISAT2 (v 2.1.0) [36] was used to map the reads of each sample to the reference genome, and Samtools (v.1.9) [56] was used to sort and convert the SAM files to BAM. StringTie (v 2.0.6) [37] was then used to assemble and merge the transcripts of each sample. Gffcompare (v0.11.5) [57] was used to compare the merged transcripts with the reference annotation file in GTF, and StringTie (v 2.0.6) was used to estimate transcript abundances with the options '-e -B -p 20'. The abundance results were folders that ended with '.balltown', and prepDE.py was used to compare the folders. DESeq2 [58] was used for differentially expressed genes (DEGs)

analysis with default parameters. To perform differential expression analysis using a genome model, the cDNA reads were mapped against the genome assembly by using HISAT2. HTSeq [59] was used to count the number of reads mapped against the annotated genes.

Funding

This work was granted and supported by the National Natural Science Fund (U20A2051, 31760648 and 31860638), and Guangxi Natural Science Foundation (AB18221120), and Guangxi Distinguished scholars Program (201835), and Qihuang High-level Talent Team Training Projects of Guangxi University of Chinese Medicine: Application of Systems Biology in Chinese Medicine Research (2021005).

Author contributions

J.Z. and Q.L. developed the concept of this study; Q.L., J.R. and Z.L. designed the research; X.W. performed genome assembly, gene annotation and evolutional analysis; T.F. analysed the transcriptome data; H.S. analysed the bloodsucking characteristic; X.Y. analysed the mechanical and visual characteristics; W.Z., C.Q., X.M. J.L., L.L. and K.C. helped during sample collection; Q.L., Z.L., J.Y., W.C., and J.Y. discussed the results and implications; X.M., L.L., W.X. and J.L. helped with the medicinal applications; X.L. and H.L. performed the statistical analysis; Z.L. drafted the manuscript.S.R. revised the manuscript. All authors read and approved the final manuscript.

Competing interests

The authors declare no competing interests.

Data Availability

The genomic and transcriptomic Illumina data, Nanopore sequencing, and HiC data were uploaded at NCBI with BioProject number: PRJNA762643. The genomes and gene annotations of the three leeches were under figshare with the doi: 10.6084/m9.figshare.20400729.

Abbreviations

BUSCO: Benchmarking Universal Single-Copy Orthologs; Mb: megabase pairs; KEGG: Kyoto Encyclopedia of Genes and Genomes; GO: gene ontology; NCBI: The National Center for Biotechnology Information; QV: quality value.

**Genomic features**	***H. nipponia***	***H. manillensis***	***W. pigra***
Total genome size (Mb)	203.7	157.5	181.4
Number of scaffolds[a]	11+253	13+243	11+183
Scaffold N50 (Mb)	18.5	11.9	16.2
Number of contigs	985	622	437
Contig N50 (Mb)	1.1	2.5	4.1
Number of genes	20,430	18,106	18,540
Repeat sequences	33.7%	25.3%	30.5%

[a] Number of chromosome-level scaffolds and unplaced scaffolds.

Table 1. Summary statistics for the three leech genomes.

Figures

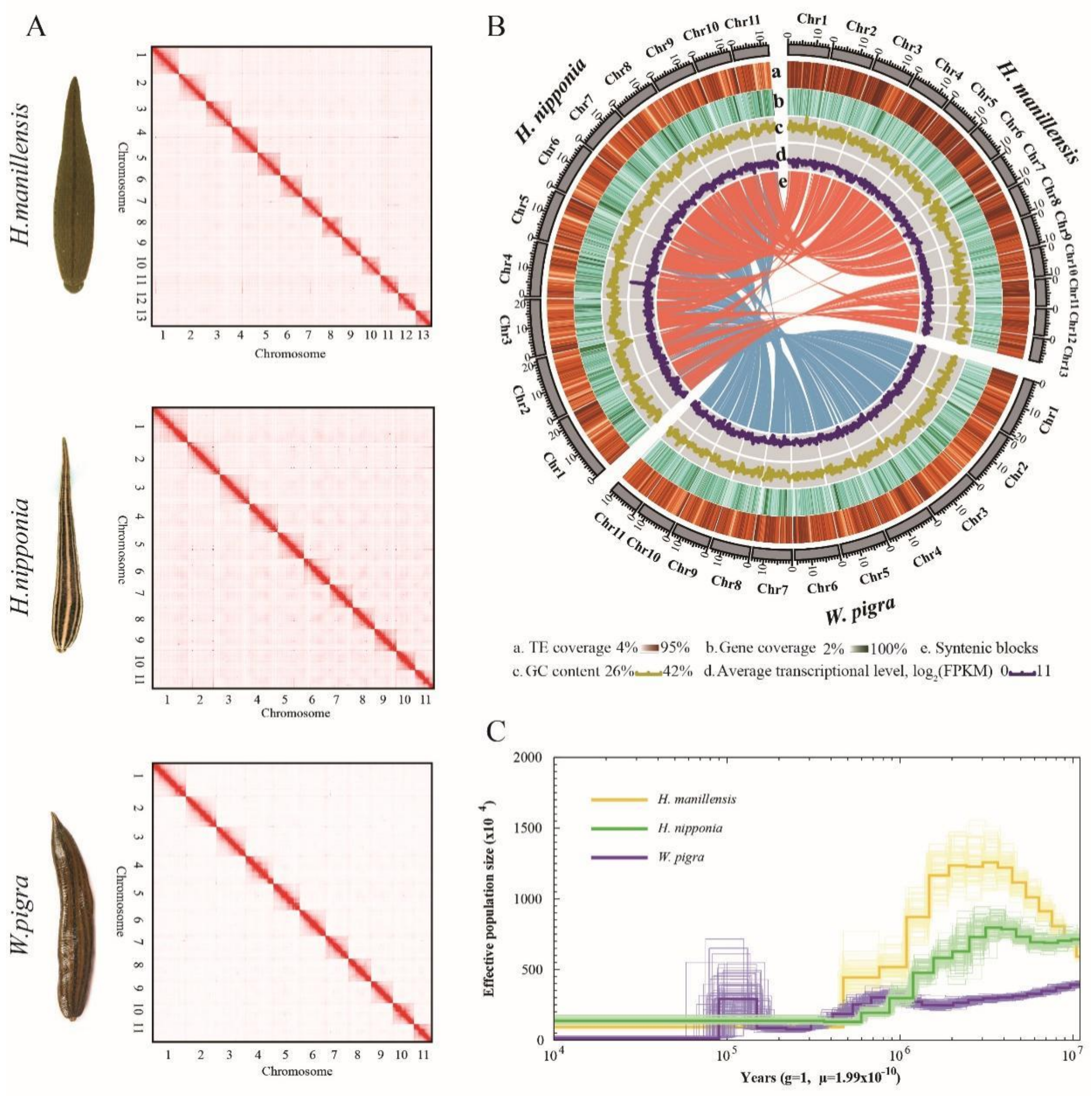

Figure 1 Genome assembly of the three leech species. A: Hi-C interactive heatmap for genome-wide organization of the three leech species. B: Comparative genomic analysis of the three leech species. Circos diagram depicts the genome characteristics. Tracks from the outer to inner circles indicate the following: chromosomes, TE coverage, gene coverage, GC content, gene expression and syntenic block (*H. nipponia* as reference). C: Demographic history inferred using PSMC.

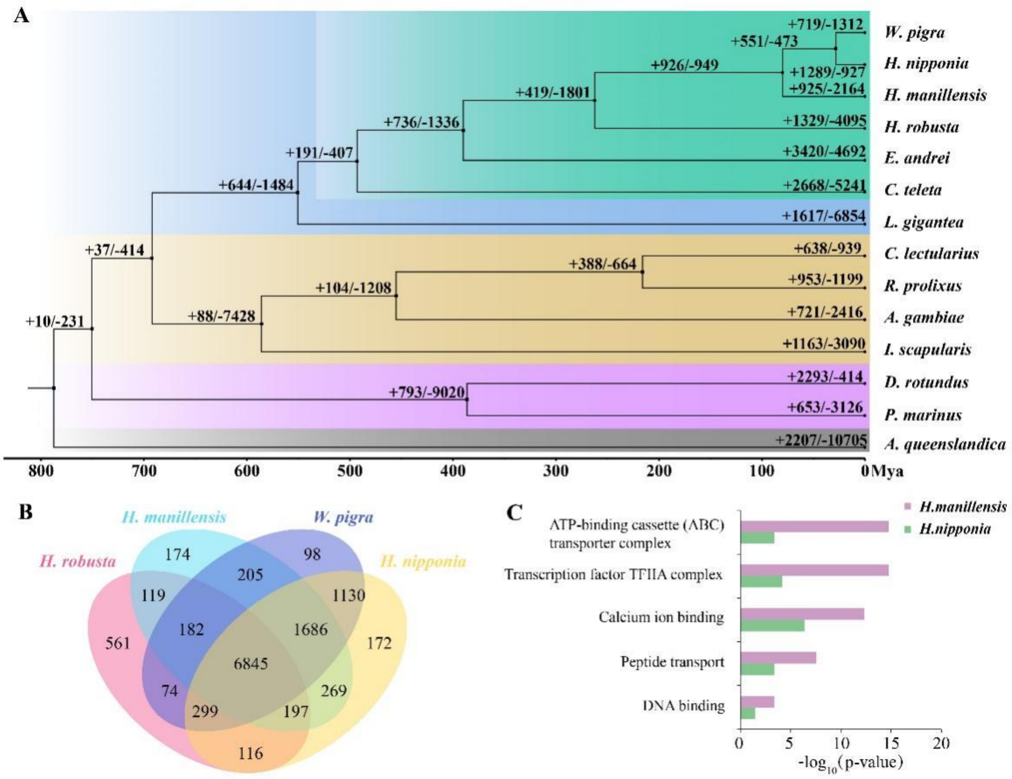

Figure 2 Phylogenetic tree and gene family analysis. A: Phylogenetic tree generated using single-copy orthologous genes. Numbers on the nodes are the numbers of expanded (+) and contracted (-) gene families. B: Venn diagram showing the numbers of detected orthologous gene families of four leech species. C: GO analysis of the sharing expanded gene families of the two bloodsucking leech species.

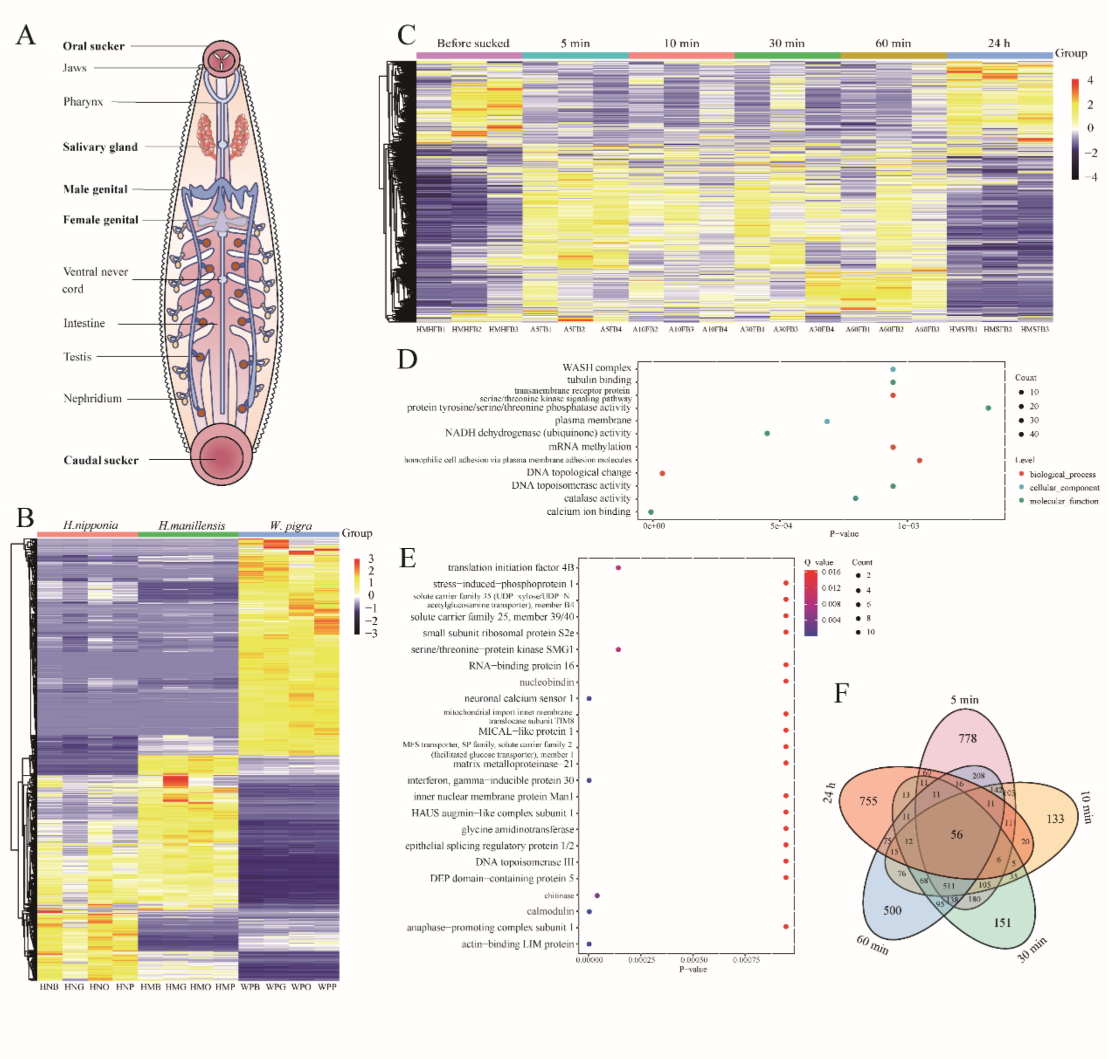

Figure 3 Transcriptome sequencing and analysis of the three leech species. A: Anatomy diagram for leech. The leech was divided into four parts according to the anatomical structure. B: Heat map of the differentially expressed genes (DEGs) in the three leech species. C: Heat map of the DEGs at different time points before bloodsucking, during bloodsucking (5-60 minutes) and after bloodsucking (24 hours) processes in *H. manillensis*. D & E: GO and KEGG analyses of the DEGs at different bloodsucking times in *H. manillensis*. F: Venn diagram showing numbers of DEGs during different bloodsucking times in *H. manillensis*.

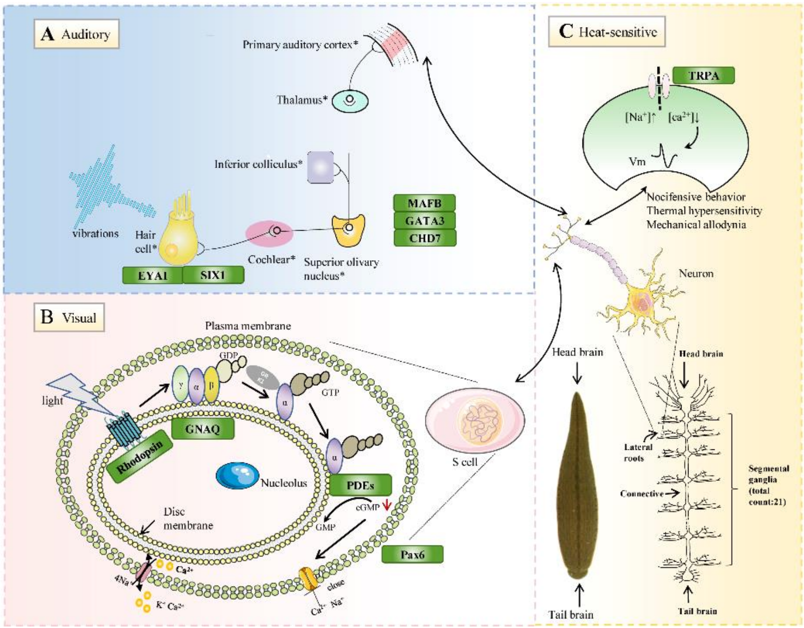

Figure 4 Molecular basis for prey location and tracing by leeches. Schematic diagram for the auditory system (A), visual system (B) and heat-sensitive channel (C) in leeches. On the basis of the genomic and transcriptional data, genes that encode known mechanical or visual receptors were identified in the three leech species. Genes identified in the leeches are labeled in the green box.

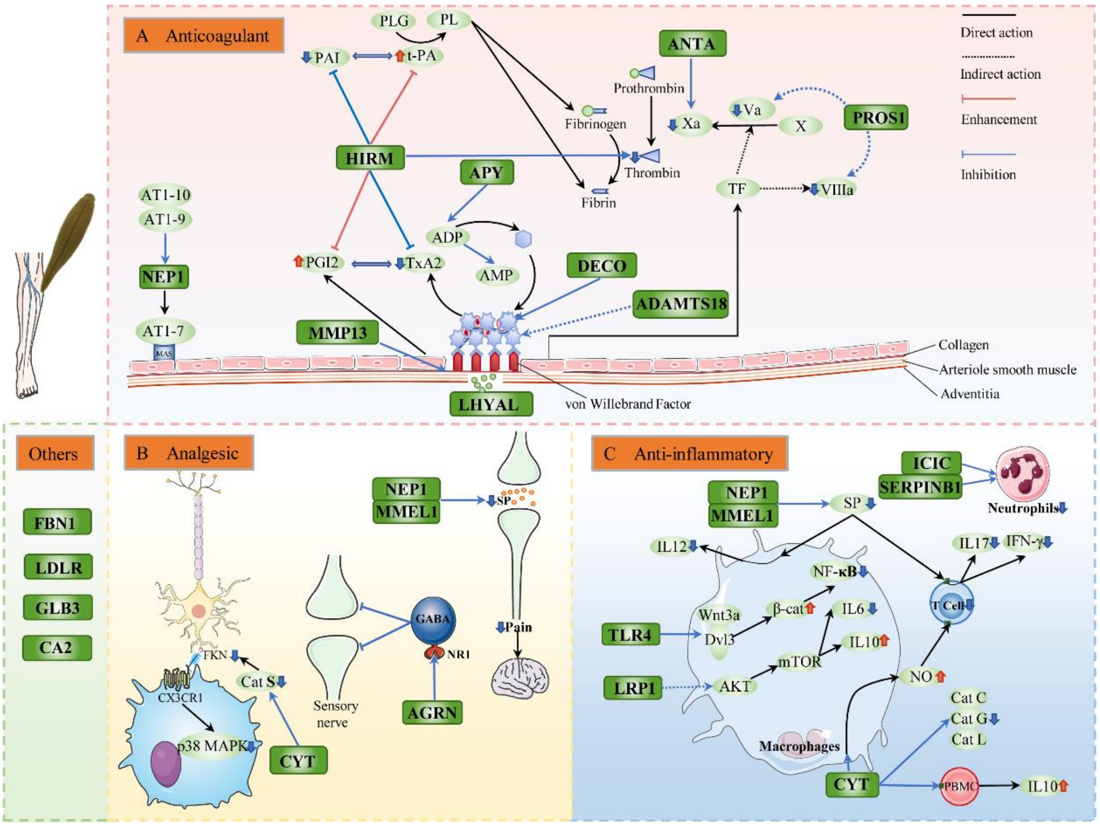

Figure 5 Molecular basis for the sanguivorous behaviours of leeches. Schematic diagram shows the anticoagulation (A), analgesic (B) and anti-inflammatory (C) processes in leeches. The solid lines represent direct interaction, and the dotted lines represent indirect interactions. Genes identified in the leeches are labeled with green boxes.

Reference

1. Jia N, Wang J, Shi W, Du L, Sun Y, Zhan W, et al. Large-Scale Comparative Analyses of Tick Genomes Elucidate Their Genetic Diversity and Vector Capacities. *Cell.* 2020;182(5):1328-40 e13. doi:10.1016/j.cell.2020.07.023.

2. Markwardt and Fritz. Hirudin As Alternative Anticoagulant- A Historical Review. *Seminars in Thrombosis & Hemostasis.* 2002;28(5):405-14.

3. Babenko VV, Podgorny OV, Manuvera VA, Kasianov AS, Manolov AI, Grafskaia EN, et al. Draft genome sequences of Hirudo medicinalis and salivary transcriptome of three closely related medicinal leeches. *BMC Genomics.* 2020;21:1-16.

4. Simakov O, Marletaz F, Cho SJ, Edsinger-Gonzales E, Havlak P, Hellsten U, et al. Insights into bilaterian evolution from three spiralian genomes. *Nature.* 2013;493(7433):526-31. doi:10.1038/nature11696.

5. Guan D-L, Yang J, Liu Y-K, Li Y, Mi D, Ma L-B, et al. Draft genome of the Asian buffalo leech Hirudinaria manillensis. *Front Genet.* 2020;10:1321.

6. Kvist S, Manzano-Marín A, Carle DD, Trontelj P and Siddall ME. Draft genome of the European medicinal leech Hirudo medicinalis (Annelida, Clitellata, Hirudiniformes) with emphasis on anticoagulants. *Sci Rep.* 2020;10(1):9885.

7. Rhie A, Walenz BP, Koren S and Phillippy AM. Merqury: reference-free quality, completeness, and phasing assessment for genome assemblies. *Genome biol.* 2020;21(1):1-27.

8. Lehmkuhl AM, Muthusamy A and Wagenaar DA. Responses to mechanically and visually cued water waves in the nervous system of the medicinal leech. *J Exp Biol.* 2018;221 4.

9. Bricaud O and Collazo A. The transcription factor six1 inhibits neuronal and promotes hair cell fate in the developing zebrafish (Danio rerio) inner ear. *J Neurosci.* 2006;26(41):10438-51. doi:10.1523/JNEUROSCI.1025-06.2006.

10. Fernald RD. Casting a genetic light on the evolution of eyes. *Science.* 2006;313(5795):1914-8.

11. Terakita A. The opsins. *Genome biol.* 2005;6(3):1-9.

12. Lenahan C, Sanghavi R, Huang L and Zhang JH. Rhodopsin: A Potential Biomarker for Neurodegenerative Diseases. *Front Neurosci.* 2020;14:326.

13. Gillette R, Huang R-C, Hatcher N and Moroz LL. Cost-benefit analysis potential in feeding behavior of a predatory snail by integration of hunger, taste, and pain. *Proc Natl Acad Sci U S A.* 2000;97(7):3585-90.

14. Pitcher T, Lang S and Turner J. A risk-balancing trade off between foraging rewards and predation hazard in a shoaling fish. *Behav Ecol Sociobiol.* 1988;22(3):225-8.

15. Buhren BA, Schrumpf H, Hoff N-P, Bölke E, Hilton S and Gerber PA. Hyaluronidase: from clinical applications to molecular and cellular mechanisms. *Eur J Med Res.* 2016;21(1):1-7.

16. Syed AA and Mehta A. Target specific anticoagulant peptides: a review. *Int J Pept Res Ther.* 2018;24(1):1-12.

17. Muñoz MC, Montes R, Hermida J, Orbe J, Paramo JA and Rocha E. Effect of the administration of recombinant hirudin and/or tissue‐plasminogen activator (t‐PA) on endotoxin‐induced disseminated intravascular coagulation model in rabbits. *Br J Haematol.* 1999;105(1):117-21.

18. Dunwiddie C, Thornberry N, Bull H, Sardana M, Friedman P, Jacobs J, et al. Antistasin, a leech-derived inhibitor of factor Xa: kinetic analysis of enzyme inhibition and identification of the reactive site. *J Biol Chem.* 1989;264(28):16694-9.

19. O'Brien LM, Mastri M and Fay PJ. Regulation of factor VIIIa by human activated protein C and protein S: inactivation of cofactor in the intrinsic factor Xase. *Blood.* 2000;95(5):1714-20.

20. Strand K, Knapp JE, Bhyravbhatla B and Royer Jr WE. Crystal structure of the hemoglobin dodecamer from Lumbricus erythrocruorin: allosteric core of giant annelid respiratory complexes. *J Mol Biol.* 2004;344(1):119-34.

21. Asgari S, Luo Y, Akbari A, Belbin GM, Li X, Harris DN, et al. A positively selected FBN1 missense variant reduces height in Peruvian individuals. *Nature.* 2020;582(7811):234-9. doi:10.1038/s41586-020-2302-0.

22. Li H. Minimap2: pairwise alignment for nucleotide sequences. *Bioinformatics.* 2018;34(18):3094-100. doi:10.1093/bioinformatics/bty191.

23. Chen S, Zhou Y, Chen Y and Gu J. fastp: an ultra-fast all-in-one FASTQ preprocessor. *Bioinformatics.* 2018;34(17):i884-i90. doi:10.1093/bioinformatics/bty560.

24. Marcais G and Kingsford C. A fast, lock-free approach for efficient parallel counting of occurrences of k-mers. *Bioinformatics.* 2011;27(6):764-70. doi:10.1093/bioinformatics/btr011.

25. Vurture GW, Sedlazeck FJ, Nattestad M, Underwood CJ, Fang H, Gurtowski J, et al. GenomeScope: fast reference-free genome profiling from short reads. *Bioinformatics.* 2017;33(14):2202-4.

26. Kolmogorov M, Yuan J, Lin Y and Pevzner PA. Assembly of long, error-prone reads using repeat graphs. *Nat Biotechnol.* 2019;37(5):540-6.

27. Vaser R, Sović I, Nagarajan N and Šikić MJGr. Fast and accurate de novo genome assembly from long uncorrected reads. *Genome Res.* 2017;27(5):737-46.

28. Walker BJ, Abeel T, Shea T, Priest M, Abouelliel A, Sakthikumar S, et al. Pilon: an integrated tool for comprehensive microbial variant detection and genome assembly improvement. *PloS One.* 2014;9(11):e112963.

29. Burton JN, Adey A, Patwardhan RP, Qiu R, Kitzman JO and Shendure J. Chromosome-scale scaffolding of de novo genome assemblies based on chromatin interactions. *Nat Biotechnol.* 2013;31(12):1119-25. doi:10.1038/nbt.2727.

30. Simao FA, Waterhouse RM, Ioannidis P, Kriventseva EV and Zdobnov EM. BUSCO: assessing genome assembly and annotation completeness with single-copy orthologs. *Bioinformatics.* 2015;31(19):3210-2. doi:10.1093/bioinformatics/btv351.

31. Stanke M, Diekhans M, Baertsch R and Haussler D. Using native and syntenically mapped cDNA alignments to improve de novo gene finding. *Bioinformatics.* 2008;24(5):637-44. doi:10.1093/bioinformatics/btn013.

32. Korf I. Gene finding in novel genomes. *BMC Bioinformatics.* 2004;5(1):59. doi:10.1186/1471-2105-5-59.

33. Haas BJ, Delcher AL, Mount SM, Wortman JR, Smith RK, Jr., Hannick LI, et al. Improving the Arabidopsis genome annotation using maximal transcript alignment assemblies. *Nucleic Acids Res.* 2003;31(19):5654-66. doi:10.1093/nar/gkg770.

34. She R, Chu JS, Wang K, Pei J and Chen N. GenBlastA: enabling BLAST to identify homologous gene sequences. *Genome Res.* 2009;19(1):143-9. doi:10.1101/gr.082081.108.

35. Birney E, Clamp M and Durbin R. GeneWise and Genomewise. *Genome Res.* 2004;14(5):988-95. doi:10.1101/gr.1865504.

36. Kim D, Paggi JM, Park C, Bennett C and Salzberg SL. Graph-based genome alignment and genotyping with HISAT2 and HISAT-genotype. *Nat Biotechnol.* 2019;37(8):907-15.

37. Pertea M, Pertea GM, Antonescu CM, Chang T-C, Mendell JT and Salzberg SL. StringTie enables improved reconstruction of a transcriptome from RNA-seq reads. *Nat Biotechnol.* 2015;33(3):290-5.

38. Haas BJ, Salzberg SL, Zhu W, Pertea M, Allen JE, Orvis J, et al. Automated eukaryotic gene structure annotation using EVidenceModeler and the Program to Assemble Spliced Alignments. *Genome Biol.* 2008;9(1):R7. doi:10.1186/gb-2008-9-1-r7.

39. Bairoch A, Apweiler R, Wu CH, Barker WC, Boeckmann B, Ferro S, et al. The universal protein resource (UniProt). *Nucleic Acids Res.* 2005;33 suppl_1:D154-D9.

40. Mao X, Cai T, Olyarchuk JG and Wei L. Automated genome annotation and pathway identification using the KEGG Orthology (KO) as a controlled vocabulary. *Bioinformatics.* 2005;21(19):3787-93. doi:10.1093/bioinformatics/bti430.

41. Ogata H, Goto S, Sato K, Fujibuchi W, Bono H and Kanehisa M. KEGG: Kyoto Encyclopedia of Genes and Genomes. *Nucleic Acids Res.* 1999;27(1):29-34. doi:10.1093/nar/27.1.29 %J Nucleic Acids Research.

42. Quevillon E, Silventoinen V, Pillai S, Harte N, Mulder N, Apweiler R, et al. InterProScan: protein domains identifier. *Nucleic Acids Res.* 2005;33 Web Server issue:W116-20. doi:10.1093/nar/gki442.

43. Lagesen K, Hallin P, Rodland EA, Staerfeldt HH, Rognes T and Ussery DW. RNAmmer: consistent and rapid annotation of ribosomal RNA genes. *Nucleic Acids Res.* 2007;35(9):3100-8. doi:10.1093/nar/gkm160.

44. Lowe TM and Eddy SR. tRNAscan-SE: A Program for Improved Detection of Transfer RNA Genes in Genomic Sequence. *Nucleic Acids Res.* 1997;25(5):955-64. doi:10.1093/nar/25.5.955 %J Nucleic Acids Research.

45. Nawrocki EP and Eddy SR. Infernal 1.1: 100-fold faster RNA homology searches. *Bioinformatics.* 2013;29(22):2933-5. doi:10.1093/bioinformatics/btt509.

46. Almagro Armenteros JJ, Tsirigos KD, Sonderby CK, Petersen TN, Winther O, Brunak S, et al. SignalP 5.0 improves signal peptide predictions using deep neural networks. *Nat Biotechnol.* 2019;37(4):420-3. doi:10.1038/s41587-019-0036-z.

47. Kall L, Krogh A and Sonnhammer EL. Advantages of combined transmembrane topology and signal peptide prediction--the Phobius web server. *Nucleic Acids Res.* 2007;35 Web Server issue:W429-32. doi:10.1093/nar/gkm256.

48. Viklund H, Bernsel A, Skwark M and Elofsson A. SPOCTOPUS: a combined predictor of signal peptides and membrane protein topology. *Bioinformatics.* 2008;24(24):2928-9. doi:10.1093/bioinformatics/btn550.

49. Emms DM and Kelly S. OrthoFinder: phylogenetic orthology inference for comparative genomics. *Genome Biol.* 2019;20(1):238. doi:10.1186/s13059-019-1832-y.

50. De Bie T, Cristianini N, Demuth JP and Hahn MW. CAFE: a computational tool for the study of gene family evolution. *Bioinformatics.* 2006;22(10):1269-71. doi:10.1093/bioinformatics/btl097.

51. Edgar RC. MUSCLE: a multiple sequence alignment method with reduced time and space complexity. *BMC Bioinformatics*. 2004;5:113. doi:10.1186/1471-2105-5-113.

52. Stamatakis A. RAxML version 8: a tool for phylogenetic analysis and post-analysis of large phylogenies. *Bioinformatics*. 2014;30(9):1312-3. doi:10.1093/bioinformatics/btu033.

53. Yang Z. PAML 4: phylogenetic analysis by maximum likelihood. *Mol Biol Evol*. 2007;24(8):1586-91. doi:10.1093/molbev/msm088.

54. Wang Y, Tang H, Debarry JD, Tan X, Li J, Wang X, et al. MCScanX: a toolkit for detection and evolutionary analysis of gene synteny and collinearity. *Nucleic Acids Res*. 2012;40(7):e49. doi:10.1093/nar/gkr1293.

55. Bolger AM, Marc L and Bjoern U. Trimmomatic: a flexible trimmer for Illumina sequence data. Bioinformatics. 2014; 15:2114-20.

56. Li H, Handsaker B, Wysoker A, Fennell T, Ruan J, Homer N, et al. The Sequence Alignment/Map format and SAMtools. *Bioinformatics*. 2009;25(16):2078-9.

57. Pertea G and Pertea M. GFF utilities: GffRead and GffCompare. *F1000Research*. 2020;9.

58. Love MI, Huber W and Anders S. Moderated estimation of fold change and dispersion for RNA-seq data with DESeq2. *Genome Biol*. 2014;15(12):550.

59. Simon A, Theodor PP and Wolfgang H. HTSeq—a Python framework to work with high-throughput sequencing data. *Bioinformatics*. 2015; 2:166-9.

Click here to access/download;Figure;Figure 1.pdf

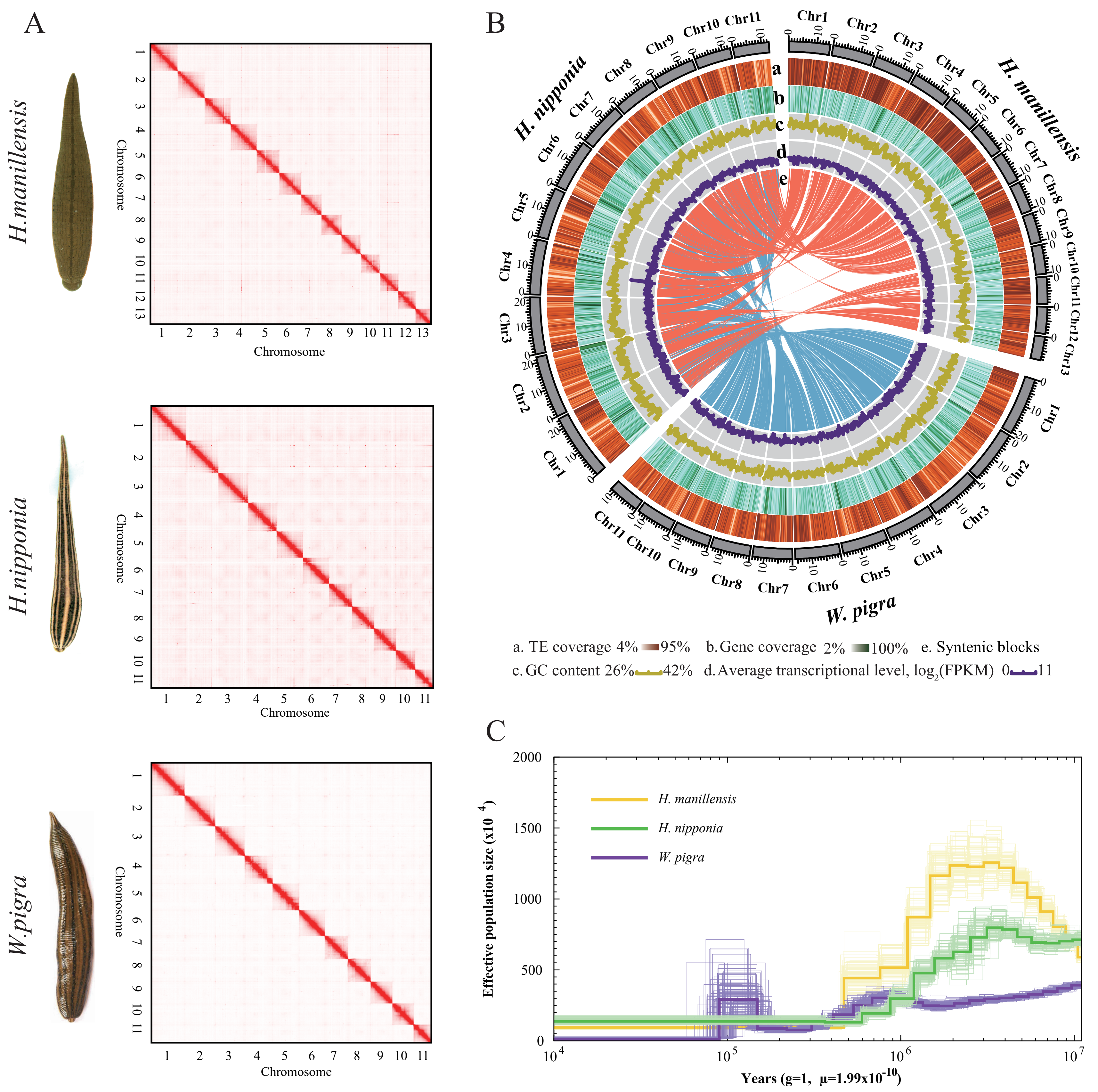

Click here to access/download;Figure;Figure 2.pdf

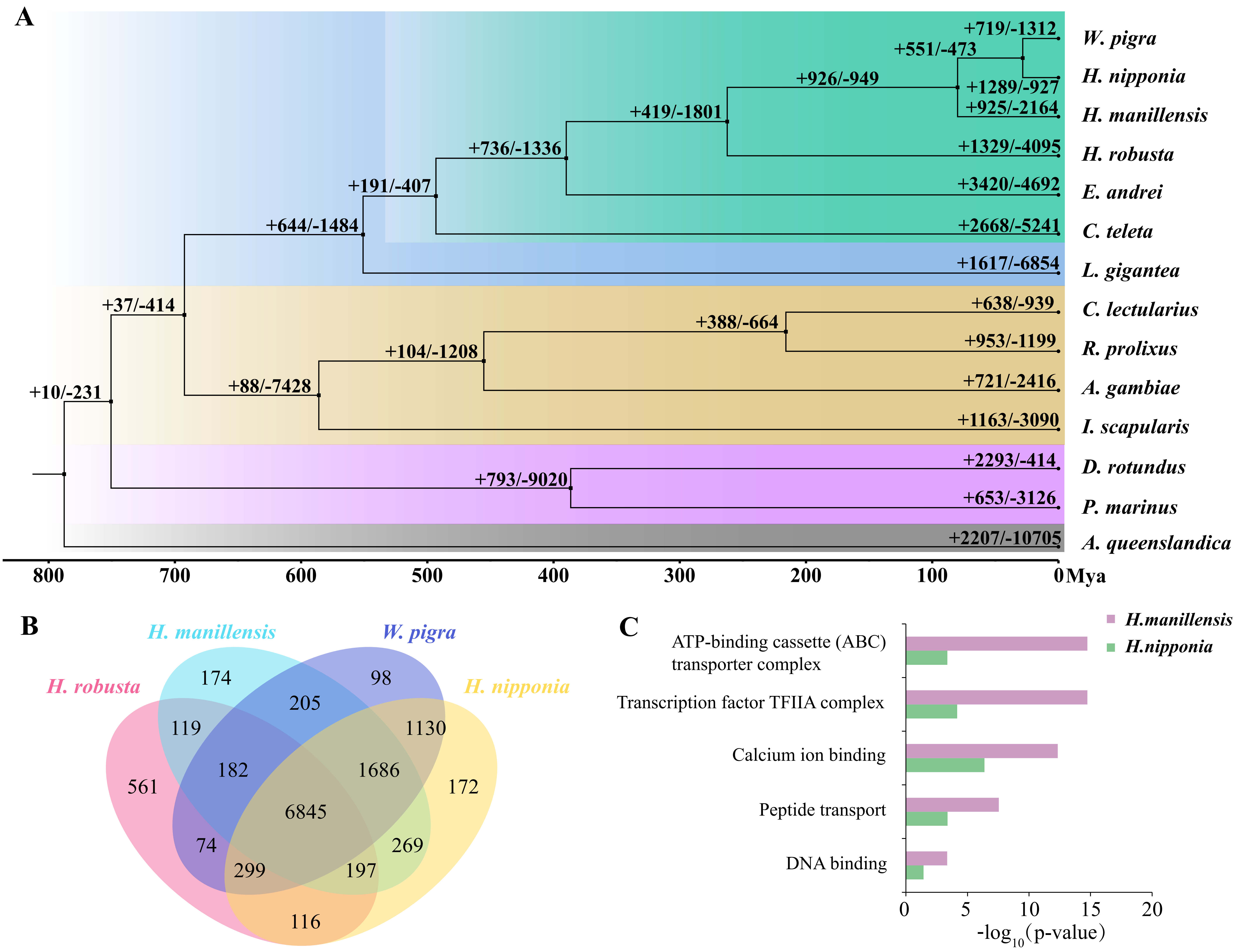

Click here to access/download;Figure;Figure 3.pdf

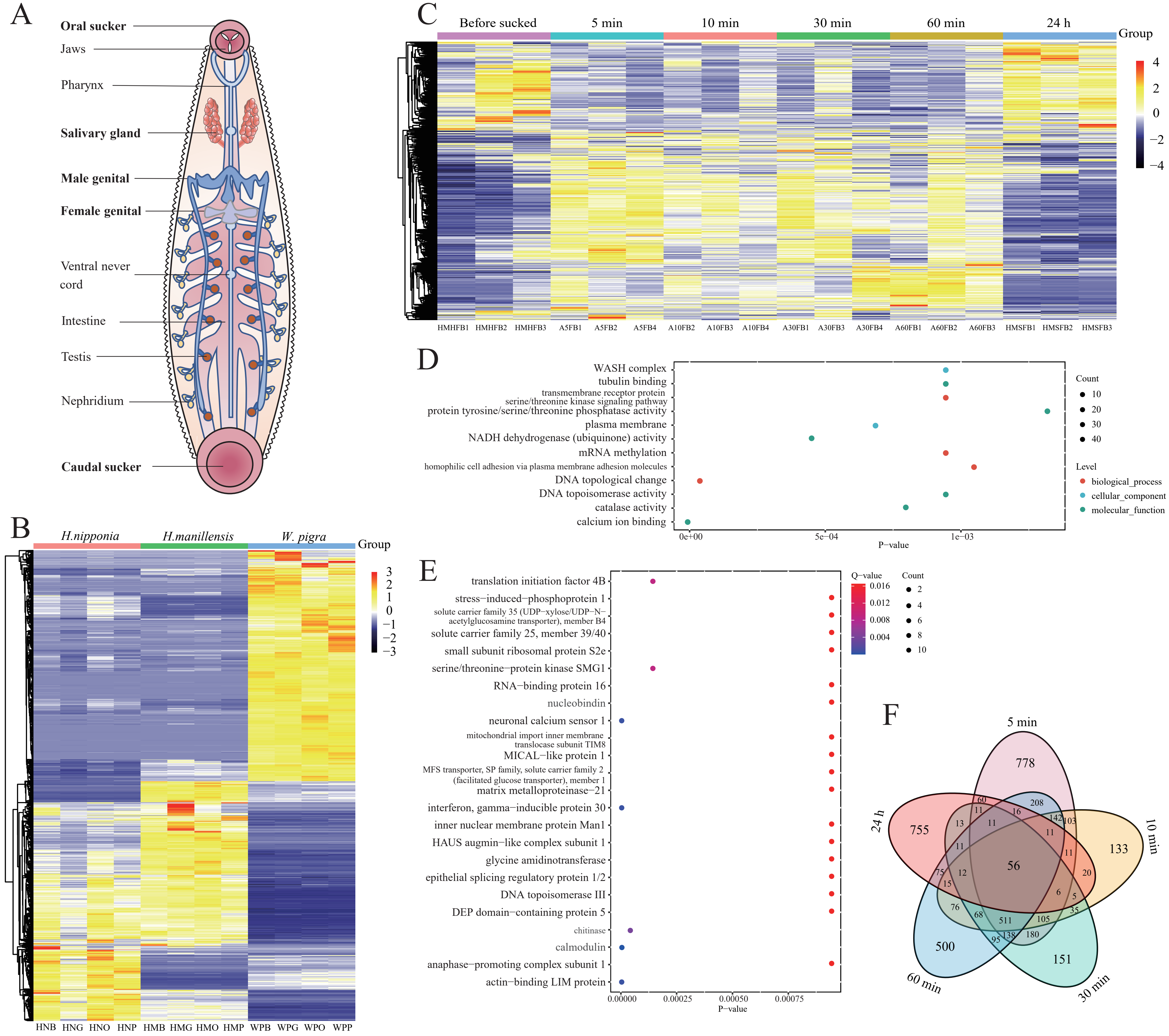

Click here to access/download;Figure;Figure 4.pdf

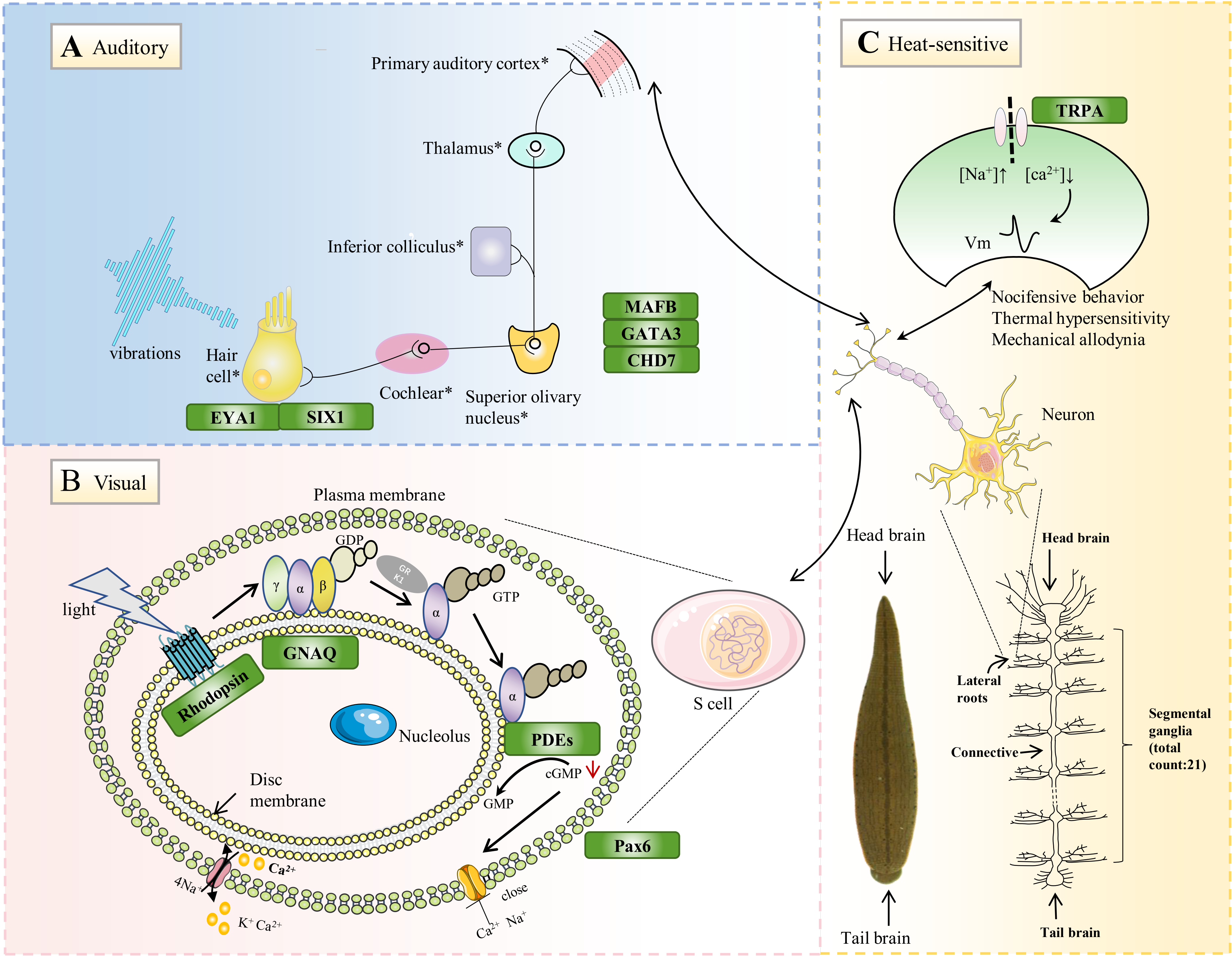

Click here to access/download;Figure;Figure 5.pdf

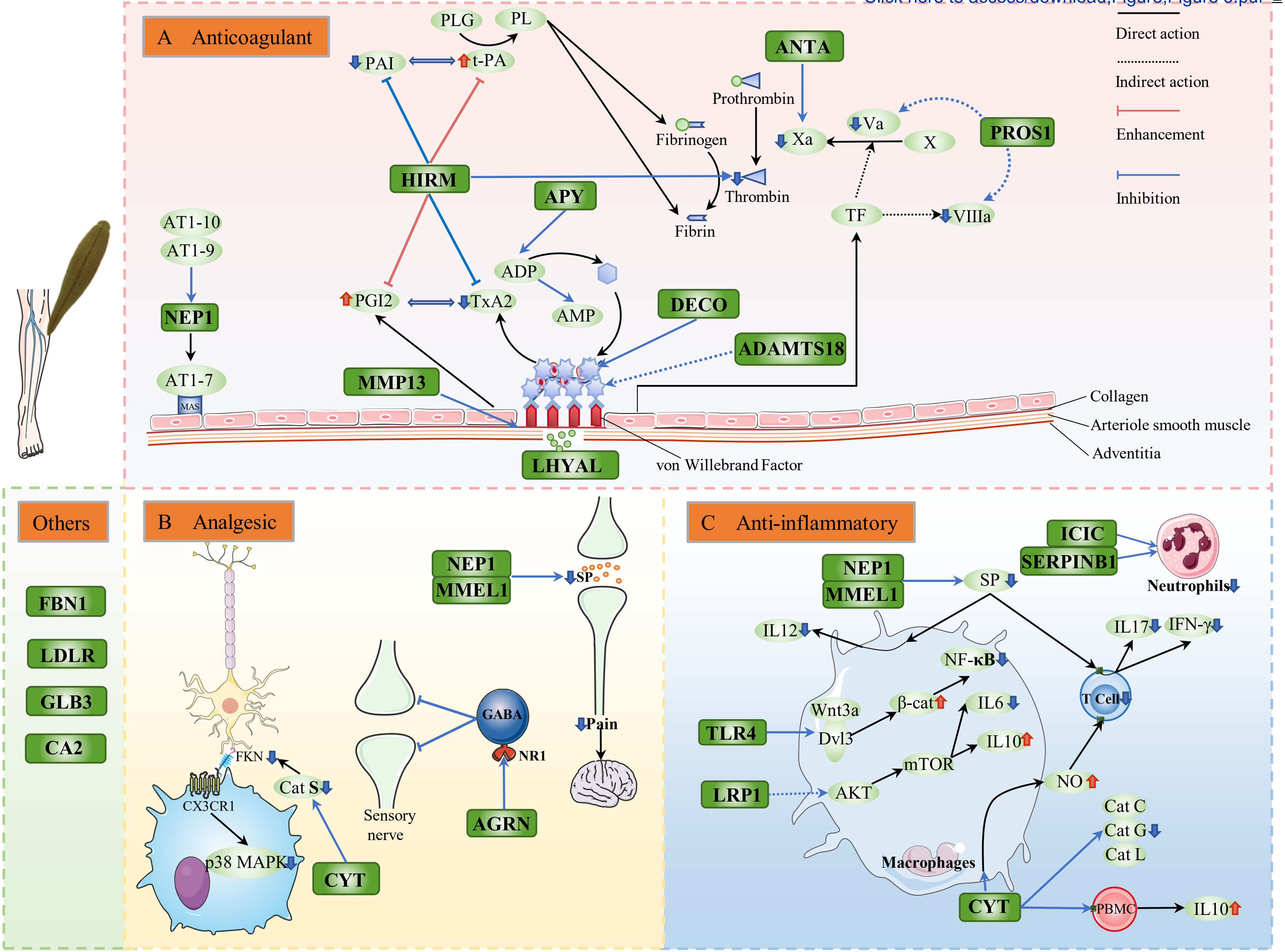

Click here to access/download
Supplementary Material
Supplementary_Materials.docx

Click here to access/download;Personal Cover;Coverletter.docx

State Key Laboratory for Conservation and Utilization of Subtropical Agro-bioresources, Guangxi University
100 Daxue Road, Nanning 530005
PR China
Jul. 29, 2022

Dear Editor,

Our manuscript entitled "**Molecular mechanisms underlying hematophagia revealed by comparative analyses of leech genomes**", which we would like to be considered for publication in *Gigascience.*

Leeches are aquatic predators that are widely distributed worldwide, and they display various fascinating behavioural and physiological characteristics that are of evolutionary, biochemical and pharmaceutical interest. Leeches have been used in the treatment of diverse ailments since ancient times in Greece, Rome, Arabia and China. Leeches secrete the most potent natural thrombin inhibitor, hirudin, and a few of bioactive proteins have been identified in its salivary gland help to eliminate microcirculation disorders, restore vascular permeability, remove hypoxia, decrease blood pressure and detoxify the organism via antioxidant pathway, which have been regarded as vital treatment of diverse human ailments.In this study, we have made exciting breakthroughs that reveal the diverse biological adaptations of leeches with respect to environmental perception and sanguivorous behaviours.

Briefly, we provided chromosome-level genome assemblies of three leech species (bloodsucking *Hirudo nipponia* and *Hirudinaria manillensis*, and non-bloodsucking *Whitmania pigra*) widely used as Chinese traditional medicine. we sequenced and analyzed the transcriptome of 32 samples (three replicates for each sample) including different developmental stages, tissues and a series of dynamic bloodsucking process at 5 time points. We found that the two bloodsucking leeches shared similar gene expression patterns and a common genetic basis of hematophagia. Specially, the fibrillin family (*FBN1*) and the globin family (*GLB3*) underwent significantly expansion and showed increased expression levels in bloodsucking leeches, which potentially provided adaptions for rapid swelling during the bloodsucking process and long-time storage of host blood, separately.

Our high-quality reference genomes and comprehensive catalogue may help in leech-derived candidate drug prospecting, especially for the treatment of cardiovascular and cerebrovascular diseases, thrombus, pain and other potential disorders.

We deeply appreciate your consideration of our manuscript, and expect for receiving comments from the reviewers. Please let us know if we can provide any additional information. We look forward to hearing from you soon.

Thank you and best regards.

Sincerely,

Qingyou Liu

Corresponding author: Prof. Qingyou Liu, Guangxi University;

E-mail: qyliu-gene@gxu.edu.cn
