# Supplementary material for: Molecular mechanisms underlying hematophagia revealed by comparative analyses of leech genomes
Source: Gigascience. 2023 Apr 11;12:giad023. doi: 10.1093/gigascience/giad023 (PMC10087013; doi:10.1093/gigascience/giad023)
Supplement: giad023_GIGA-D-22-00200_Revision_1 [file giad023_giga-d-22-00200_revision_1.pdf]

GigaScience

Molecular mechanisms underlying hematophagia revealed by comparative analyses of leech genomes

--Manuscript Draft--

**Manuscript Number:**	GIGA-D-22-00200R1
**Full Title:**	Molecular mechanisms underlying hematophagia revealed by comparative analyses of leech genomes
**Article Type:**	Data Note
**Funding Information:**	National Natural Science Foundation of China (U20A2051) — Not applicable<br>National Natural Science Foundation of China (31760648) — Prof Qingyou Liu<br>National Natural Science Foundation of China (31860638) — Prof Qingyou Liu<br>Guangxi Natural Science Foundation (AB18221120) — Prof Qingyou Liu<br>Guangxi Distinguished scholars Program (201835) — Prof Qingyou Liu<br>Qihuang High-level Talent Team Training Projects of Guangxi University of Chinese Medicine: Application of Systems Biology in Chinese Medicine Research (2021005) — Prof Qingyou Liu
**Abstract:**	Background<br>Leeches have been used in traditional Chinese medicine since pre-historic times to treat a spectrum of ailments, but very little is known about their physiological, genetic, and evolutionary characteristics.<br>Findings<br>We sequenced and assembled chromosome-level genomes of three leech species (bloodsucking Hirudo nipponia and Hirudinaria manillensis and non-bloodsucking Whitmania pigra). The dynamic population histories and genome-wide expression patterns of the two bloodsucking leech species were found to be similar. A combined analysis of the genomic and transcriptional data revealed that the bloodsucking leeches have a presumably enhanced auditory sense for prey location in relatively deep fresh water. The copy number of genes related to anticoagulation, analgesia, and anti-inflammation increased in the bloodsucking leeches, and their gene expressions responded dynamically to the bloodsucking process. Furthermore, the expanded FBN1 gene family may help in rapid body swelling of leeches after bloodsucking, and the expanded GLB3 gene family may be associated with long-term storage of prey blood in a leech's body.<br>Conclusions<br>The high-quality reference genomes and comprehensive datasets obtained in this study may facilitate innovations in the artificial culture and strain optimization of leeches.
**Corresponding Author:**	Qingyou Liu<br>Foshan University<br>Foshan, GaungDong CHINA
**Corresponding Author Secondary Information:**	
**Corresponding Author's Institution:**	Foshan University
**Corresponding Author's Secondary Institution:**	
**First Author:**	Jinghui Zheng

Powered by Editorial Manager® and ProduXion Manager® from Aries Systems Corporation

**First Author Secondary Information:**	
**Order of Authors:**	Jinghui Zheng
	Xiaobo Wang
	Tong Feng
	Saif ur Rehman
	Xiuying Yan
	Huiquan Shan
	Xiaocong Ma
	Weiguan Zhou
	Wenhua Xu
	Liying Lu
	Jiasheng Liu
	Xier Luo
	Kuiqing Cui
	Chaobin Qin
	Weihua Chen
	Jun Yu
	Zhipeng Li
	Jue Ruan
	Qingyou Liu
**Order of Authors Secondary Information:**	
**Response to Reviewers:**	Dec. 14, 2022<br><br>To<br>The Editorial Office,<br>GigaScience<br><br>Dear editors and reviewers:<br><br>Thank you for your kind reply. We are very grateful for the reviewer's comments on our manuscript entitled "Molecular mechanisms underlying hematophagia revealed by comparative analyses of leech genomes" (Manuscript id: GIGA-D-22-00200). Indeed, your comments are very helpful in improving our manuscript. We have carefully studied your comments and tried our best to revise the manuscript to meet your approval. We think we have finished all concerns from reviewers. Here, the revisions are documented in the revised manuscript, response letter, and Supplementary Figs and Tables. The responses to the reviewers' comments are as follows.<br><br>Kind regards,<br><br>Qingyou Liu<br><br>E-mail address: qyliu-gene@gxu.edu.cn<br><br><br>Response to reviewers<br><br><br>Reviewer #1:<br>This study entitled "Molecular mechanisms underlying hematophagia revealed by comparative analyses of leech genomes" has reported three chromosome-level

Powered by Editorial Manager® and ProduXion Manager® from Aries Systems Corporation

genomes of leeches. This study has shown population changes of three leeches, and comparative analysis result of copy number of some special gene families has found some clues for divergence between bloodsucking and nonbloodsuking behaviours. The genome materials and findings of this study are indeed significant.
The quality of these three genomes are pretty well. The analysed approaches used in this paper are also well performed.

Response: Thank you very much for your supportive comments. We sincerely appreciate for the valuable comments from you.

Nevertheless, the English language of this paper indeed need to be largely revised by a native speaker. Too much errors were appeared in this paper.

Response: Thank you for your suggestion. The manuscript has been revised by professional editing company ELIXIGEN to improve the writing expression. They are highlighted with red colors and underlines. The editorial certification is listed below.

The detailed version and used library of BUSCO should be described, and the annotation BUSCO number also should be reported.

Response: The BUSCO library we used is metazoa_odb9 (2016-02-13), and the detailed BUSCO number and total BUSCO number are listed in Table S4. Please see below.

Table S4. BUSCO evaluation of the draft assemblies using metazoa_odb9 (2016-02-13) database.
H. nipponiaH. manillensisW. pigra
NumberRatioNumberRatioNumberRatio
Complete89591.5%88890.8%89791.7%
Fragmented202.0%222.2%242.5%
Missing636.5%687.0%575.8%
Total978100.0%978100%978100.0%

The format of this study should be largely revised, and RRIDs of used software should be added in this manuscript.

Response: We follows the “Data Note” format in gigascience journal to revise our manuscript as editor suggested, and all the available software RRIDs are added.

In figure 1B, the inner aligned lines only contain the alignments between H. nipponia and H. manillensis, and H. nipponia and W. pigra. Why the alignments between H. man and W.pigra are not shown in this figure?

Response: Sorry for lack of the alignments between H. manillensis and W.pigra. Now we added it. Please check it below in figure1B.

In figure 1C, I suggest authors to add the sea-level and air temperature curves in this figure.
The detailed curves you can reference this paper, Whole Genome Sequencing of Chinese White Dolphin (Sousa chinensis) for High-Throughput Screening of Antihypertensive Peptides

Response: That’s a good point. We have added the curves in figure1C. Please check it above.

Some minor suggestions:
"Here we provided three high-quality leech genomes and abundant transcriptomes which illustrated the gene expression dynamics of bloodsucking leeches including anticoagulation, analgesic, and anti-inflammation that would facilitate the understanding at the genetic level and could be crucial for drug candidate prospecting" revised to 'Here we provided three high-quality leech genomes and abundant

Powered by Editorial Manager® and ProduXion Manager® from Aries Systems Corporation

transcriptomes that illustrated the gene expression dynamics of bloodsucking leeches including anticoagulation, analgesic, and anti-inflammation, which would facilitate the understanding at the genetic level and could be crucial for drug candidate prospecting'.

Response: Thank you for your suggestion. It has been corrected and highlighted with yellow in the manuscript.

For "contained 985, 622, and 437 Mb contigs with N50 contig lengths of 1.1, 2.5, and 4.1 Mb", the decimal places through whole paper should be uniformed. Moreover, this could be an error description for '985, 622 and 437 Mb contigs'. In my opinion, 'Mb' should be removed, there is the number of contigs.

Response: Thanks for pointing this out. The decimal places are now kept to one through the whole paper. “985, 622 and 437” indeed represent the number of contigs, and ‘Mb’ has been removed.

'and we identified' changed to 'to identify'.

Response: Change made.

"we constructed the gene family" changed to "we constructed gene families".

Response: Change made.

'20430, 18106, and 18540 protein-coding genes' should be '20,430, 18,106, and 18,540 protein-coding genes'

Response: Corrected.

'restored to that' changed to 'restored to those'.

Response: Change made.

'is fundamental to understanding' revised to 'is fundamental to understand'.

Response: Corrected.

Reviewer #2: This manuscript provides some useful genomic and transcriptomic data of three leech species. And the bloodsucking characteristics of leeches are explained to some extent. However, the present version does not meet the publish criteria of GigaScience. Here are my detailed comments which may help to improve the manuscripts before it can be accepted for publication.

Response: Many thanks for your professional comments. We appreciate your time and constructive suggestions, which is very helpful for improving our manuscript. We hope our revisions in this version could meet your criteria and further receive your replies. Please check our improvements as follows.

1.Correct species classification is crucial for scientific papers. I see the author states that leeches are "arthropods" in the first sentence. It is a trivial but serious mistake, because it has long been accepted by researchers that leeched belong to Annelida. Hence it is reasonable for readers to suspect the validity of taxonomic status of all specimen involved in this manuscript. I strongly suggest that the authors firstly taking both a morphological and a molecular identification of all their leech specimens.

Response: We feel very sorry for that mistake and definitely agree with your opinion. Now we have corrected and changed “arthropods” to “annelids”.

2.As mentioned in the title as well as the whole text, the main interest of this manuscript focus on the molecular mechanisms of hematophagous behavior of leech. However, the most characteristic anticoagulants of leech such as hirudin is poorly discussed, e.g. gene structure, sequence specificity. On the contrary, FBN1 and GLB3, which is mainly discussed in the manuscript, is farfetched to explain the blood sucking

Powered by Editorial Manager® and ProduXion Manager® from Aries Systems Corporation

habit.

Response: Thank you for pointing this out. Following your suggestions, we performed analysis on hirudin genes about gene structure, sequence specificity and gene expression pattern. Three hirudin genes were identified in H. manillensis, and one is identified in both H. nipponia and W. Pigra. The intron regions of HN_hirudin and WP_hirudin are inserted with transposable elements (TEs), leading to longer length than the three HM_hirudin genes without TE insertions (Fig.S11A below). Multiple sequence alignments show that the cysteine pattern of the hirudin is more conserved than the tail but less conserved than the beginning of the alignment (Fig.S11B below). Finally, we find that hirudin genes mainly express in the oral suckers of the three leeches, and the expression of HM_hiridin1 is comparatively increased at the time point of 30 minutes (Fig.S11C), which is consistent with the time of physiological coagulation.

Fig. S11. Analysis of hirudin genes in the three leeches.

We noticed that HM_hirudin3 shows "redundant" sequence in the region of the cysteine pattern. We checked the alignment of the transcriptome data and confirmed the authenticity of the "redundant" sequence (figure below). It suggests that the cysteine pattern is presumably highly plastic.

We thus described our revisions in this main text as follows:
"The gene structure and copy number of the well-known anticoagulant HIRM1 (hirudin) were different in the three leeches (Supplementary Fig. 11). The gene lengths of HN_hirudin and WP_hirudin were apparently longer than the three HM_hirudin copies, and this was mainly attributable to TE insertion into intron regions (Supplementary Fig. 11A). We performed multiple sequence alignment for five HIRM1 genes and found that the cysteine pattern of hirudin [7] is more conserved than the tail but less conserved than the beginning of the alignment (Supplementary Fig. 11B). This indicates that the cysteine pattern may have the potential to be plastic. HIRM1 was mainly expressed in the oral suckers of the three leech species, and the expression of HM_hiridin1 comparatively increased at 30 min (Supplementary Fig. 11C), which is consistent with the time of physiological coagulation."

3. The authors mentioned in the Introduction "Currently, a few leech species genomic data including one non-blood sucking leech (Helobdella robusta) and low coverage genome sequence data of two lineages Amynthas cortices have been published [3-6],…". Actually, besides Helobdella robusta there have been four leech species deposited in the GenBank: Hirudo medicinalis (accession numbers: GCA_011800805.1, GCA_903470615.1); Hirudo verbana (GCA_020137395.1); Hirudinaria manillensis (ASM1534595v1); Whitmania pigra (GCA_021613335.1, GCA_021650995.1). The latter two overlapped with the species used in this manuscript. It is clear that the authors noticed the GenBank data since H. medicinalis and H. manillensis are listed in the Table S1. It is not clear, however, that why they drop two more famous species H. verbana and W. pigra, but instead using a distantly related species Helobdella robusta and even earthworms?

Response: We apologize for the incomplete collection of genomic information. We change the sentence to "the genomic data of several leech species have been published" and add all the genomic information of the mentioned species into Table S1 as well as the available references into the main content.

Table S1. Comparison of genome assembly to other publicized leech genomes.
SpeciesTotal genome size (Mb)Scaffolds NumberScaffold N50Number of genes
H. nipponia203.711+25318.5 MB20,430
H. manillensis157.513+24311.9 MB18,106
W. Pigra181.411+18316.2 MB18,540
H. medicinalis (Genbank accession numbers:GCA_903470615.1)177.019,92950.4 KB35,166
H. medicinalis (GCA_011800805.1)187.614,04297.8 KB14,596
H. manillensis (ASM1534595v1)151.84672.3 MB17,865
H. verbana (GCA_020137395.1)235.059,8178.4 KB-

	W. pigra (GCA_021613335.1)178.84832.0 MB-<br>W. pigra (GCA_021650995.1)17710,050728.0 KB26,743<br>H. robusta (GCA_000326865.1)2281,9913.6MB23,400<br><br>Additional reference:<br>Tong, Lei, et al. "The genome of medicinal leech (Whitmania pigra) and comparative genomic study for exploration of bioactive ingredients." BMC Genomics 23.1 (2022): 1-13.<br><br>4.There are obvious differences in genome size between different species and different papers of the same species. It is suggested that the authors explaining the reason or examine whether there is DNA contamination from microorganisms or other species. For example, the H. nipponia has a much larger genome than the other species in Hirudinidae. Is there potential contamination by bacteria genome?<br><br>Response: In our analysis, we removed these contigs that covered more than 50% of the bacterial genome sequences deposited in NCBI (see the method “Genome assembly and assessment”). Thus, bacterial contamination likely has little effect on genome size in this study. In recent studies on large genomes, such as african lungfish[1] (~40Gb) and Mexican axolotl[2] (~32Gb), TEs mainly contribute to their huge genomes. In our leech genomes, the maximum difference in the lengths of the TE sequences is 28.8Mb (accounts for 62.3% of the difference of genome sizes) between H. nipponia, and H. manillensis, suggesting the TE contents maybe a major driver to the larger leech genome. Consistent with the speculation, TEs are responsible for the intron expansion in hirudin genes of H. nipponia and W. pigra compared to them of H. manillensis (Question 3).<br>For the same species, it is known that the genome size and contig N50 could be improved by using more sequencing reads. For example, we provided ~48 Gb compared with ~12Gb in Guan et al.[3] for H. manillensis, and we obtained more ~5.7Mb genome sequences as well as longer contig N50 (2.5Mb vs 2.3Mb). Besides, long-read sequencing technology are usually used to resolve many repeats longer than the second-sequencing reads, leading to more complete assembly results. For example, we assembled larger genome (181.4Mb vs 177Mb) for W. pigra and more repetitive contents (30.5% vs 23%) than Tong et al[4]. In addition, the different assembling strategies applied in different papers presumably partly contribute to the difference of genome sizes for the same species.<br>[1]Wang, Kun, et al. "African lungfish genome sheds light on the vertebrate water-to-land transition." Cell 184.5 (2021): 1362-1376.<br>[2]Nowoshilow, Sergej, et al. "The axolotl genome and the evolution of key tissue formation regulators." Nature 554.7690 (2018): 50-55.<br>[3]Guan, De-Long, et al. "Draft genome of the Asian buffalo leech Hirudinaria manillensis." Front. Genet. 10 (2020): 1321.<br>[4]Tong, Lei, et al. "The genome of medicinal leech (Whitmania pigra) and comparative genomic study for exploration of bioactive ingredients." BMC Genomics 23.1 (2022): 1-13.<br><br>5.The number of chromosome of leech varies between species. The HI-C analysis in this work was based on an assumed number of these three species, which could lead to a specious result. It is recommended that the author provide verification on the chromosome number of those new reported genomes before they draw conclusions from the HI-C analysis.<br><br>Response: We apologize that the software used for contig clustering was incorrectly written as Lachesis (the corresponding reference is about 3D-DNA software), which is in fact 3D-DNA. Just as you said, Lachesis indeed was based on assumed chromosome number. 3D-DNA automatically cluster the contigs without setting chromosome numbers. We tried to identify their karyotypes using experimental method but failed previously, so we describe the the HiC clustering results as “pseudo-chromosomes”.
**Additional Information:**	
**Question**	**Response**
Are you submitting this manuscript to a	No

Powered by Editorial Manager® and ProduXion Manager® from Aries Systems Corporation

special series or article collection?	
**Experimental design and statistics**<br><br>Full details of the experimental design and statistical methods used should be given in the Methods section, as detailed in our Minimum Standards Reporting Checklist. Information essential to interpreting the data presented should be made available in the figure legends.<br><br>Have you included all the information requested in your manuscript?	Yes
**Resources**<br><br>A description of all resources used, including antibodies, cell lines, animals and software tools, with enough information to allow them to be uniquely identified, should be included in the Methods section. Authors are strongly encouraged to cite Research Resource Identifiers (RRIDs) for antibodies, model organisms and tools, where possible.<br><br>Have you included the information requested as detailed in our Minimum Standards Reporting Checklist?	Yes
**Availability of data and materials**<br><br>All datasets and code on which the conclusions of the paper rely must be either included in your submission or deposited in publicly available repositories (where available and ethically appropriate), referencing such data using a unique identifier in the references and in the "Availability of Data and Materials" section of your manuscript.<br><br>Have you have met the above requirement as detailed in our Minimum Standards Reporting Checklist?	Yes

Powered by Editorial Manager® and ProduXion Manager® from Aries Systems Corporation

Manuscript

Click here to access/download;Manuscript;leeches-final-revise.docx

Molecular mechanisms underlying hematophagia revealed by comparative analyses of leech genomes

Jinghui Zheng*[2], Xiaobo Wang*[3,4], Tong Feng*[3,4], Saif ur Rehman[3], Xiuying Yan[3], Huiquan Shan[3], Xiaocong Ma[2], Weiguan Zhou[6], Wenhua Xu[2], Liying Lu[2], Jiasheng Liu[2], Xier Luo[3,4], Kuiqing Cui[3], Chaobin Qin[3], Weihua Chen[5], Jun Yu[7], Zhipeng Li[3], Jue Ruan[†4], Qingyou Liu[†1]

1. Guangdong Provincial Key Laboratory of Animal Molecular Design and Precise Breeding, School of Life Science and Engineering, Foshan University, Foshan 528225, China.

2. Department of Cardiology, Ruikang Hospital Affiliated to Guangxi University of Chinese Medicine, Nanning 530011, China.

3. State Key Laboratory for Conservation and Utilization of Subtropical Agro-bioresources, Guangxi University, Nanning 530004, China.

4. Genome Analysis Laboratory of the Ministry of Agriculture, Agricultural Genomics Institute, Chinese Academy of Agricultural Sciences, Shenzhen, Guangdong, China.

5. Department of Bioinformatics and Systems Biology, College of Life Science and Technology, Huazhong University of Science and Technology, Wuhan, Hubei, China.

6. Biological Institute of Guangxi Academy of Sciences, Nanning 530007, China.

7. CAS Key Laboratory of Genome Sciences and Information, Beijing Institute of Genomics, Chinese Academy of Sciences, Beijing 100101, China.

*These authors contributed equally: Jinghui Zheng, Xiaobo Wang and Tong Feng

†Corresponding author. E-mail: qyliu-gene@gxu.edu.cn; ruanjue@caas.cn

ORCID iDs: Jinghui Zheng [0000-0001-7243-0147]; Xiaobo Wang [0000-0001-6754-7404]; Tong Feng [0000-0002-6056-0590]; Saif ur Rehman [0000-0002-5407-3112]; Xiuying Yan [0000-0002-1379-9013]; Huiquan Shan [0000-0001-8824-1703]; Xiaocong Ma [0000-0002-3507-0485]; Wenhua Xu [0000-0002-6030-7077]; Liying Lu [0000-0003-3234-6455]; Jiasheng Liu [0000-0001-7762-3685]; Kuiqing Cui [0000-0002-9777-1084]; Chaobin Qin [0000-0002-7186-6608]; Jun Yu [0000-0002-2702-055X]; Zhipeng Li [0000-0003-3190-2253]; Jue Ruan [0000-0003-3713-3192]; Qingyou Liu [0000-0003-3265-540X].

Abstract

Background

Leeches have been used in traditional Chinese medicine since pre-historic times to treat a spectrum of ailments, but very little is known about their physiological, genetic, and evolutionary characteristics.

Findings

We sequenced and assembled chromosome-level genomes of three leech species (bloodsucking *Hirudo nipponia* and *Hirudinaria manillensis* and non-bloodsucking *Whitmania pigra*). The dynamic population histories and genome-wide expression

patterns of the two bloodsucking leech species were found to be similar. A combined analysis of the genomic and transcriptional data revealed that the bloodsucking leeches have a presumably enhanced auditory sense for prey location in relatively deep fresh water. The copy number of genes related to anticoagulation, analgesia, and anti-inflammation increased in the bloodsucking leeches, and their gene expressions responded dynamically to the bloodsucking process. Furthermore, the expanded *FBN1* gene family may help in rapid body swelling of leeches after bloodsucking, and the expanded *GLB3* gene family may be associated with long-term storage of prey blood in a leech's body.

Conclusions

The high-quality reference genomes and comprehensive datasets obtained in this study may facilitate innovations in the artificial culture and strain optimization of leeches.

Introduction

Leeches are obligate blood-feeding annelids distributed from tropical to subarctic regions around the globe. Hematophagous species, such as bats, ticks, and mosquitos, are the most versatile vectors capable of transmitting a wide range of pathogens (such as protozoa, bacteria, nematodes, fungi, and viruses) to humans, livestock, and wildlife [1]; however, leeches have been found to transmit few infectious diseases. Furthermore, leeches have been used in traditional Chinese medicine since pre-historic times to treat

a spectrum of ailments. Leeches secrete the most potent natural thrombin inhibitor, hirudin [2], and exhibit a variety of fascinating behavioral and physiological characteristics that are of interest from an evolutionary, biochemical, and pharmaceutical point of view. Leeches have also developed persistent adaptive strategies and characteristics to perceive their environment during long-term evolution. Leeches continuously receive sensory information from their surroundings by either mechanical or visual sensation to locate and target their prey. Additionally, the sanguivorous behavior of leeches is capable of reducing natural host reflexes (blood coagulation, pain, and inflammation) during bloodsucking [3]. To understand prey localization, sanguivorous behavior, and medicinal value of leeches, fundamental knowledge of leech genomes and genetic diversity is necessary, and this would undoubtedly open new avenues for research on leech biology, host interactions, and control strategies at the molecular level. Heretofore, leech research was primarily focused on strain optimization, artificial culture, and identification and development of therapeutic strategies; however, well-annotated genomes or genetic data are still unavailable. Currently, the genomic data of several leech species have been published [3-7], but not to the chromosome level. Here we provided three high-quality leech genomes (*Hirudo nipponia* (NCBI:txid42736), *Hirudinaria manillensis* (NCBI:txid1348078) and *Whitmania pigra* (NCBI:txid486152)) and abundant transcriptomes that illustrated the gene expression dynamics of bloodsucking leeches

including anticoagulation, analgesic, and anti-inflammation, which would facilitate the understanding at the genetic level and could be crucial for drug candidate prospecting.

Results

Genome assemblies

We used the Nanopore platform for sequencing and performed genome assembly for three leech species, *H. nipponia*, *H. manillensis*, and *W. pigra* (Fig. 1A), that are ubiquitously used in the Chinese pharmacopeia. The assemblies for *H. nipponia*, *H. manillensis*, and *W. pigra* contained 985, 622, and 437 contigs with N50 contig lengths of 1.1, 2.5, and 4.1 Mb, respectively (Table 1). On the basis of the Hi-C data, the contigs of *H. nipponia*, *H. manillensis*, and *W. pigra* were consolidated into scaffolds with N50 lengths of 18.5, 11.9, and 16.2 Mb, respectively, and they comprised 11, 13, and 11 pseudo-chromosomes, respectively (Table 1 and Fig. 1B). The sizes of the final genome assemblies of *H. nipponia*, *H. manillensis*, and *W. pigra* were approximately 203.7, 157.5, and 181.4 Mb, respectively; the results were similar to the estimated genome sizes based on k-mer (K = 17) analysis (Supplementary Fig. 1). The assemblies had larger scaffold N50 sizes and lower scaffold numbers, indicating higher continuity than previously reported genomes (Supplementary Table 1).

Furthermore, we aligned the short reads and transcriptome assemblies to the genomes to assess the completeness of our genome assemblies and found that more than 98% of

the reads and 95% of transcriptome data were mapped to the assemblies (Supplementary Tables 2 and 3). We also estimated the completeness and accuracy of the final assemblies and found that 91.5%, 90.8%, and 91.7% of the BUSCO orthologs were captured for *H. nipponia*, *H. manillensis*, and *W. pigra*, respectively (Supplementary Table 4). We used Merqury [8] to obtain QV scores of 35.8 for *W. pigra*, 33.4 for *H. nipponia*, and 32.1 for *H. manillensis*. We combined the results of the de novo and homolog-based approaches and identified about 25–33% of repetitive sequences in the leech genomes (Supplementary Table 5 and Supplementary Fig. 3).

Population history of the leeches

We used pairwise sequentially Markovian coalescent analysis to infer changes in the effective population size (Ne) of the ancestral leech populations. The population of the non-bloodsucking leech *W. pigra* underwent two expansions, whereas the populations of the two bloodsucking leeches, *H. nipponia* and *H. manillensis*, experienced only one expansion (Fig. 1C). The different fluctuations in Ne may hint at different environmental adaptations of the two types of leeches. Interestingly, the Ne of the bloodsucking leeches began to decrease at the onset of the Pleistocene (~2 MYA), which was characterized by repeated cycles of glaciations. The glaciations probably reduced the contact between leeches and animal hosts, resulting in a decline in the size of the bloodsucking leech populations.

Gene annotation and gene family construction

Three methods, namely, de novo, homology-based, and transcriptome-based gene predictions, were used to identify a total of 20,430, 18,106, and 18,540 protein-coding genes in the *H. nipponia*, *H. manillensis*, and *W. pigra* genomes, and the total CDS lengths were 32.3, 27.4, and 32.3 Mb and mean CDS lengths were 1,739, 1,647, and 1,801 bp, respectively (Supplementary Tables 6–8). Further, the gene sets were aligned against UniProt, InterPro, and KEGG databases, and about 88% of the genes were functionally assigned or annotated (Supplementary Table 9). Besides, thousands of ncRNA genes and secreted genes were also identified in each of the three leech genomes (Supplementary Tables 10–13).

We constructed gene families and performed a phylogenetic analysis of 14 species. *W. pigra* and *H. nipponia* shared a common ancestor about 50 MYA, whereas *H. manillensis* diverged at an earlier date (Fig. 2A). This implies that the blood-sucking behavior may have existed in the ancestors of leeches, but this behavior was lost in the lineage of *W. pigra*. Four leech species were found to share most of their gene families, which is consistent with evolutionary relationships (Fig. 2B). Additionally, a total of 1,289, 925, and 719 expanded and 927, 2,164, and 1,312 contracted gene families were identified in the *H. nipponia*, *H. manillensis*, and *W. pigra* genomes, respectively. The GO analysis depicted that expanded gene families such as the ATP-binding cassette transporter complex, transcription factor IIA complex, and calcium ion binding functions were significantly enriched in both bloodsucking leech species (Fig. 2C).

Transcriptome dynamics

To analyze the gene expression patterns of the three leeches, we sequenced and analyzed the transcriptomes of 32 samples (three replicates for each sample) of different developmental stages, tissues, and a series of bloodsucking behaviors at five different time points (Supplementary Figs. 5–9). We used DEseq2 to identify differentially expressed genes (DEGs) between the non-bloodsucking and bloodsucking leeches and found that most of the DEGs of the two bloodsucking leeches shared similar expression patterns (Fig. 3B). We further investigated the transcriptomic dynamics during bloodsucking in *H. manillensis*. Mostly, the DEGs responded quickly after bloodsucking and continuously changed in the following 60 min (Fig. 3C, F). After 24 h, the expression patterns of these DEGs were virtually restored to those of the pre-bloodsucking group (Fig. 3C). Furthermore, the GO and KEGG pathway analyses showed that most of the DEGs were significantly enriched to calcium, indicating that calcium-related regulation may play an important role in the bloodsucking behavior of leeches (Fig. 3D, E).

Genetic basis of prey location for leeches

Leeches are efficient predators because they can use their mechanical and auditory systems to acquire information and locate their prey. The associated genes were identified by homolog-based functional annotation in leeches (Fig. 4). Among the hearing-related genes of leeches (Fig. 4A), *SIX1* plays a crucial role in audio sensation, and we found a single copy of *SIX1* in the non-bloodsucking leeches and two or four copies of *SIX1* in the bloodsucking leeches (Supplementary Table 14). The expression

pattern of *SIX1* was generally higher in the bloodsucking leeches than in the non-bloodsucking leeches (Supplementary Fig. 10), which clearly indicates that the bloodsucking leeches may possess better auditory perception.

Similarly, detection of visual signals in the eye is attributable to various mechanisms, with the coordinated involvement of genes and their related proteins or enzymes; intriguingly, *PDE6D*, which encodes the delta subunit of rod-specific photoreceptor phosphodiesterase, was detected in the non-bloodsucking leeches and not found in the bloodsucking leeches (Supplementary Table 14). Thus, we speculated that the bloodsucking leeches potentially strengthened audition and weakened vision to hide in relatively deep fresh water.

Genetic basis of the sanguivorous behavior of bloodsucking leeches

Leeches avoid detection by hosts during the bloodsucking process by executing three key operations: inhibition of blood coagulation, suppression of inflammation, and alleviation of pain. We identified the genes related to each process (Fig. 5) and found that the total copy number of these genes was higher in the bloodsucking leeches than in the non-bloodsucking leeches (Supplementary Table 14).

Leeches inhibit hemagglutination mainly via three ways: suppressing the thrombin cascade (*HIRM1*, *ANTA*, and *PROS1*), inhibiting platelet aggregation (*DECO*, *MMP13*, *ADAMTS18*, *APY*, and *HIRM1*), and dilating vessels (*NEP1* and *HIRM1*) (Fig. 5A). The gene structure and copy number of the well-known anticoagulant *HIRM1* (hirudin) were

different in the three leeches (Supplementary Fig. 11). The gene lengths of HN_hirudin and WP_hirudin were apparently longer than the three HM_hirudin copies, and this was mainly attributable to TE insertion into intron regions (Supplementary Fig. 11A). We performed multiple sequence alignment for five *HIRM1* genes and found that the cysteine pattern of hirudin [7] is more conserved than the tail but less conserved than the beginning of the alignment (Supplementary Fig. 11B). This indicates that the cysteine pattern may have the potential to be plastic. *HIRM1* was mainly expressed in the oral suckers of the three leech species, and the expression of HM_hiridin1 comparatively increased at 30 min (Supplementary Fig. 11C), which is consistent with the time of physiological coagulation.

We found that the expressions of two hyaluronidase (*LHYAL*) copies were the highest in only 5 min during the bloodsucking process (Supplementary Fig. 12). As expected, the gene expressions of five antistasin (*ANTA*) copies and two *PROS1* copies were relatively higher during the process of bloodsucking (5–60 min; Supplementary Fig. 12). Besides, all gene copies of *ADAMTS18* and *NEP1* showed peak expression levels in 10 min, suggesting that they may play a potentially pivotal role in the inhibition of hemagglutination.

During the bloodsucking process, leeches also produced agrin (*AGRN*), cystatin (*CYT*), neprilysin-1 (*NEP1*), and membrane metalloendopeptidase like 1 (*MMEL1*) (Fig. 5B), which may reduce pain sensitivity and help the leeches to avoid recognition by the host. Generally, all copies of *AGRN* and *MMEL1* as well as *CYT* showed high expressions

during the bloodsucking process (Supplementary Fig. 11). Further analysis showed that the leeches also expressed various anti-inflammatory genes, such as *NEP1*, *MMEL1*, *CYT*, eglin (*ICIC*), cystatin (*CYT*), Leuko*CYT*e elastase inhibitor (*SERPINB1*), Toll-like receptor 4 (*TLR4*), and lipoprotein receptor-related protein 1 (*LRP1*) (Fig. 5C). Surprisingly, the expression levels of most *TLR4* and *LRP1* copies decreased quickly at the beginning of the bloodsucking process and increased after bloodsucking (24 h; Supplementary Fig. 11). This indicates that leeches always maintain anti-inflammatory proteins for swift release into the prey body.

Moreover, we noted that *GLB3* and *FBN1* were the most expanded genes associated with sanguivorous behavior in the bloodsucking leeches. Seven or eight copies of *GLB3* were tandemly arranged in the two bloodsucking leeches, with only one copy in *W. pigra* and no copy in *Helobdella robusta* (Supplementary Table 13 and Supplementary Fig. 13). Moreover, the expression levels of the *GLB3* family increased during the bloodsucking process. In particular, three *GLB3* copies displayed significant expression level changes after the bloodsucking process (Supplementary Fig. 12). This could presumably explain why leeches can store prey blood in their body for months. Twelve copies of *FBN1* were detected in the two bloodsucking species, and only four or zero copies were found in the non-bloodsucking leeches (Supplementary Table 15). Generally, the *FBN1* family showed a continual increase in their expressions during the bloodsucking process (Supplementary Fig. 13), indicating that *FBN1* may be associated with the adaptability of leech body swelling after bloodsucking.

Discussion

Precise non-redundant reference genomes with verified annotations are critical for functional as well as evolutionary analyses, and, indeed, it is still a challenge to produce an accurate chromosome-level assembly, particularly for leech chromosomes. Although leeches have been used to treat diverse ailments since ancient times, most of our information on them is based on psychometrics. A comprehensive catalog of their genomes and gene expression patterns is fundamental to understand the genetic basis of their behavior and will be crucial for drug candidate prospecting. Although the genome of medicinal leech has been sequenced in several studies, the assembly results are fragmented [3-7]. However, in this study, we developed three chromosome-level genome assemblies for *H. nipponia*, *H. manillensis*, and *W. pigra* by integrating short-read sequencing, Nanopore sequencing, and Hi-C technology.

Leeches are efficient predators because of their specialized predation adaptation, with acute senses such as hearing, vision, and chemosensation. Leeches trace and locate their prey via mechanical and visual cues from water waves on the basis of S cells [9]. Ethological experiments have shown that leeches can quickly identify and locate the source of sound by analyzing the distribution of water waves [9]. Among hearing-related genes, *SIX1* mediates the relative numbers of sensory hair cells and statoacoustic ganglion neurons [10]. Overexpression of *SIX1* could result in more hair cells [10]. In our study, higher expression of *SIX1* in the bloodsucking leeches rather than the non-bloodsucking leeches clearly indicated that the bloodsucking leeches may possess

better auditory perception. Furthermore, genes that encode opsin had very early origins and were recruited repeatedly during eye evolution [11]. The opsin family can be divided into seven subfamilies, and rhodopsin and Gq-coupled opsin/melanopsin are the most abundant proteins in rod cells [12]. Phototransduction is initiated when rhodopsin absorbs photons and triggers the exchange of GDP for GTP on the G-protein, which leads to an increase in cGMP hydrolysis by the phosphodiesterase (PDE) complex [13]; surprisingly, *PDE6D*, which encodes the delta subunit of rod-specific photoreceptor phosphodiesterase, was present in the non-bloodsucking leeches but not in the bloodsucking leeches. We demonstrated that bloodsucking leeches possibly prefer to enhance audition and hide in relatively deep fresh water for prey.

In many species of invertebrates [14] or vertebrates [15], the choice of low-risk feeding seems to be evaluated as a cost-benefit analysis influenced by hunger cues that face immediate risks, including nociception, that may lead to identification by the host. To prevent detection by hosts throughout the bloodsucking process, leeches performed three crucial operations: inhibition of blood coagulation, suppression of inflammation, and pain relief. Hyaluronidase (*LHYAL*) boosts the diffusion and penetration of bioactive substances into tissues, and it can be used to ameliorate various complications associated with hyaluronic acid [16]. Hirudin (*HIRM1*) not only prevents fibrinogen clotting but also hinders other thrombin-catalyzed hemostatic reactions and activation of thrombin-induced platelets [17]. Additionally, hirudin can dissolve clots that have been already formed by promoting the release of T-PA [18], so it may help in thrombus

clearance. Antistasin (*ANTA*) can inhibit the function of coagulation factor Xa [19], and protein S (*PROS1*) blocks anticoagulant protease coenzyme C and factor VIII [20]. Moreover, throughout the bloodsucking process, leeches can activate anti-inflammatory proteins that may lower pain sensitivity and avoid host detection. Similarly, genes related to sanguivorous behavior such as *GLB3* and *FBN1* are essential for bloodsucking leeches. *GLB3,* which is associated with oxygen binding and carrier, heme, and iron ion binding, is involved in the formation of the hemoglobin complex [21], whereas *FBN1*, which is a major structural component of microfibrils, has been found to be the largest influential factor for height-associated variation in a human population [22]. In this study, we found that the copy number of genes related to sanguivorous behaviors was higher in the bloodsucking leeches than in the non-bloodsucking leeches. Furthermore, the expressions of these genes responded dynamically to the bloodsucking process.

Overall, we have provided three leech genomes with optimal assemblies and raised some profoundly interesting questions on the environmental perception and sanguivorous behaviors of leeches. The chromosome-level reference genomes and underlying genetic mechanisms may provide insights into the genetic basis of the bloodsucking lifestyle of leeches. The comprehensive genomic and transcriptomic datasets may serve as a powerful platform to facilitate innovations in the artificial culture and strain optimization of leeches, identification of novel bioactive compounds, and candidate drug prospecting.

Methods

DNA isolation, Nanopore library preparation, and sequencing

Three leech species, namely, *Hirudo nipponia*, *Hirudinaria manillensis*, and *Whitmania pigra*, were obtained from the bank of Changjiang River, and their intestinal tracts were removed and washed with saline solution. The genomic DNA was collected using the DNeasy Blood & Tissue Kit (Qiagen, Wroclaw, Poland). The DNA quality was assessed, a long-read library was constructed (insert size, 20 kb), and Nanopore PromethION platform was used to perform long-read sequencing. Hi-C was performed using the following protocol: The leech tissues were fixed in 1% formaldehyde solution. Nuclear chromatin was obtained from the fixed tissue and digested using *Hind*III (New England Biolabs, NEB, USA). The overhangs were blunted with bio-14-dCTP (Invitrogen, California, USA) and Klenow enzyme (NEB). After dilution and re-ligation using T4 DNA ligase (NEB), the genomic DNA was extracted and sheared to 350–500 bp with a Bioruptor (Diagenode, Belgium). Then, the biotin-labeled DNA fragments were enriched with streptavidin beads (Invitrogen).

Genome size estimation

The genome size was estimated using 17-mer analysis. The short reads were mapped to the genomes of bacteria and leeches by using Minimap2 v2.17-r941 (RRID:SCR_018550) [23]. The reads that aligned best to the bacterial genomes were filtered. Fastp v0.20.0 (RRID:SCR_016962) [24] was used to filter the low-quality

reads. Jellyfish v2.3.0 (RRID:SCR_005491) [25] was used to divide the short reads into 17-mers and calculate 17-mer frequency. The 17-mer distributions of the three leeches generated using GenomeScope (RRID:SCR_017014) [26] followed Poisson distribution. The genome sizes were estimated by dividing the total number of 17-mers by the peak of the distribution and found to be 206 Mb, 155 Mb, and 172 Mb for *H. nipponia*, *H. manillensis*, and *W. pigra*, respectively.

Genome assembly and assessment

Nanopore long reads (~43 Gb for *H. nipponia*, ~48 Gb for *H. manillensis*, and ~47 Gb for *W. pigra*) were used to establish de novo genome assemblies by using Flye v2.6 (RRID:SCR_017016) [27]. Three rounds of correction were conducted using Racon v1.4.7 (RRID:SCR_017642) [28] with the default parameters based on alignments of long reads by using Minimap2 v2.17-r941 (RRID:SCR_018550) [23]. The resulting assemblies were further polished using two rounds of Pilon v1.23 (RRID:SCR_014731) [29]. Contigs that covered more than 50% of the bacterial genome sequences were filtered. Finally, 3D-DNA (RRID:SCR_017227) [30] was used to hierarchically cluster the contigs and obtain pseudo-chromosome assemblies. The completeness and accuracy of the final assemblies were estimated using BUSCO v5.3.2 (RRID:SCR_015008) [31], Merqury (v1.3) [8], and short read alignment.

Repeat annotation

Both de novo and homology approaches were used to identify repetitive sequences in the leech genomes. RepeatModeler v1.0.11 (RRID:SCR_015027) [32] was used to construct the de novo libraries. Then, RepeatMasker (RRID:SCR_012954) [32] was run for the three leech genomes by using the de novo libraries and a known repeat library (Repbase-20181026, RRID:SCR_021169). A total of 25–33% repeat content was obtained by combining the annotation results of the two approaches.

Gene and functional annotation

Three gene prediction methods based on de novo prediction, homologous genes, and transcriptomes were used to annotate protein-coding genes in the three leech genomes. Two de novo programs, Augustus v3.0.3 (RRID:SCR_008417) [33] and SNAP v2006-07-28 (RRID:SCR_007936) [34], were used to predict genes in the repeat-masked genome sequences. Transcriptome assemblies processed with PASA r20140417 (RRID:SCR_014656) [35] were used to train gene model parameters for the two de novo programs. For homology-based prediction, protein sequences from *Capitella teleta*, *H. robusta*, and *Eisenia andrei* were aligned over the leech genomes by using tblastn (e-value < 10-5). GenblastA (RRID:SCR_020951) [36] was used to cluster adjacent high-scoring pairs from the same protein alignments, and GeneWise v2.4.1 (RRID:SCR_015054) [37] was used to identify accurate gene structures. After quality control and filtering, reads from all RNA libraries were mapped to the leech genomes by using hisat2 v2.1.0 (RRID:SCR_015530) [38], and StringTie v2.0.6 (RRID:SCR_016323) [39] was subsequently used to predict the gene models. All

predicted genes from the three approaches were combined with EVM r2012-06-25 (RRID:SCR_014659) [40] to generate high-confidence gene sets.

To obtain gene function annotations, SwissProt and TrEMBL [41] protein databases were searched using blastp (RRID:SCR_001010) (e-value < 1e-05). The best blastp hits were used to assign homology-based gene functions. KOBAS v3.0.3 (RRID:SCR_006350) [42] was used to search the KEGG [43] database for KO assignments. The functional classification of GO categories and InterPro entries was performed using InterProScan v5.39-77.0 (RRID:SCR_005829) [44].

Annotation of ncRNAs

RNAmmer v1.2 (RRID:SCR_017075) [45] was used to identify the rRNA genes. tRNAscan-SE v.2.0.5 (RRID:SCR_010835) [46] was used to annotate the tRNA genes, and tRNAs decoding 20 standard amino acids were reserved. Other non-coding RNAs, including miRNAs and snRNAs, were detected using Infernal v1.1.2 (RRID:SCR_011809) [47]. All programs were run with default parameters.

Prediction of secreted proteins

For the secreted protein analysis, three methods, SignalP 5.0 (RRID:SCR_015644) [48], Phobius (RRID:SCR_015643) [49], and SPOCTOPUS [50], were used. SignalP 5.0 (RRID:SCR_015644) focuses on the prediction of signal peptides (SPs), and the other two algorithms can predict both transmembrane regions and SPs. The protein with at

least one SP predicted using at least two out of the three methods was identified as a secreted protein.

Gene family construction

The following 14 species were compared to construct the gene families: *Amphimedon queenslandica*, *Anopheles gambiae*, *C. teleta*, *Cimex lectularius*, *Desmodus rotundus*, *H. robusta*, *Ixodes scapularis*, *Lottia gigantea*, *Petromyzon marinus*, *Rhodnius prolixus*, *E. andrei*, *H. manillensis*, *W. pigra*, and *H. nipponia*. The longest transcript for each gene was selected, and OrthoFinder v2.3.3 (RRID:SCR_017118) [51] software was used to cluster the gene families on the basis of the all-versus-all blastp alignments. Expansion and contraction of the gene families were detected using CAFÉ v4.2.1 (RRID:SCR_018924) [52].

Phylogenetic tree and divergence time

To perform phylogenetic analyses, peptide alignments for each single-copy family were obtained using MUSCLE (RRID:SCR_011812) [53] and concatenated to a supergene for each species. RAxML v8.2.9 (RRID:SCR_006086) [54] with PROTGAMMAAUTO model and 100 bootstraps was used to construct the phylogenetic tree. The peptide alignments were converted to CDS sequences, which were analyzed using mcmctree in PAML v4.9 (RRID:SCR_014932) [55] package to estimate divergence time.

Syntenic analysis

MCScanX (RRID:SCR_022067) [56] with default parameters was used to detect syntenic genome regions among the three leeches, and jcvi (RRID:SCR_021641) was used to plot Figure 1B and show their syntenic relationships.

Transcriptome analysis

The total RNA was extracted from different leech parts at different developmental stages and before/after bloodsucking (each sample included three biological replicates) by using TRIzol reagent (Invitrogen Corp., Carlsbad, CA). RNA purification was performed using the RNeasy Mini Kit (Qiagen, Chatsworth, CA). Sequencing libraries were generated using the NEBNext Ultra RNA Library Prep Kit for Illumina (NEB, USA), according to the manufacturer's instructions. The libraries were sequenced on an Illumina HiSeq 4000 platform, and 150 bp paired-end reads were generated. Each sample was trimmed using Trimmomatic v.0.39 (RRID:SCR_011848) [57] with the options "ILLUMINACLIP: TruSeq2-PE.fa:2:30:10 SLIDINGWINDOW:15:30 MINLEN:110 TRAILING:30 AVGQUAL:30." After quality control, HISAT2 v 2.1.0 (RRID:SCR_015530) [37] was used to map the reads of each sample to the reference genome, and SAMtools v.1.9 (RRID:SCR_002105) [58] was used to sort and convert the SAM files to BAM. Then, StringTie v2.0.6 (RRID:SCR_016323) [38] was used to assemble and merge the transcripts of each sample. Gffcompare (v0.11.5) [59] was used to compare the merged transcripts with the reference annotation file in GTF, and StringTie v2.0.6 (RRID:SCR_016323) was used to estimate transcript abundances with the options "-e -B -p 20." The abundance results were folders that ended with

".balltown," and prepDE.py was used to compare the folders. DESeq2 (RRID:SCR_015687) [60] with default parameters was used for the analysis of DEGs. To perform differential expression analysis using a genome model, the cDNA reads were mapped against the genome assembly by using HISAT2 (RRID:SCR_015530). HTSeq (RRID:SCR_005514) [61] was used to count the number of reads mapped against the annotated genes.

Data Availability

The genomic and transcriptomic Illumina data, Nanopore sequencing, and HiC data were uploaded at NCBI with BioProject (number: PRJNA762643). The genomes and gene annotations of the three leeches were under Figshare [62]. Supporting data are deposited in the *GigaScience* database GigaDB [63], with 3 species: *Whitmania pigra* [64], *Hirudo nipponia* [65] and *Hirudinaria manillensis* [66].

Additional Files

Supplementary Fig. S1. The 17-mer distributions of three leech genomes.

Supplementary Fig. S2. Interspecific synteny analysis of three leech genomes.

Supplementary Fig. S3. TE sequence divergences of four leech genomes.

Supplementary Fig. S4. The number of endogenous viral elements (EVEs) in the genomes of humans and several hematophagous species.

Supplementary Fig. S5. Gene expression in different body parts of *H. manillensis*.

Supplementary Fig. S6. Gene expression in different body parts of *H. nipponia*.

Supplementary Fig. S7. Gene expression in different body parts of *W. pigra*.

Supplementary Fig. S8. Gene expression in different developmental stages of *H. manillensis*.

Supplementary Fig. S9. Gene expression in different developmental stages of *H. nipponia*.

Supplementary Fig. S10. Expression of genes related to prey location in three leech species.

Supplementary Fig. S11. Analysis of hirudin genes in three leech species.

Supplementary Fig. S12. Expression of bloodsucking-related genes in *H. manillensis*.

Supplementary Fig. S13. *GLB3* copies in four leech genomes.

Supplementary Table S1. Comparison of the assembled genomes with other published leech genomes.

Supplementary Table S2. Assessment of genome completeness and base accuracy on the basis of Illumina reads.

Supplementary Table S3. Mapping ratio of two random transcriptome assemblies for each of the three leech species.

Supplementary Table S4. BUSCO evaluation of the draft assemblies by using the metazoa_odb9 (2016-02-13) database.

Supplementary Table S5. Summary of the repeat contents in three leech genomes.

Supplementary Table S6. Statistics of predicted protein-coding genes in *H. nipponia* genome.

Supplementary Table S7. Statistics of predicted protein-coding genes in *H. manillensis* genome.

Supplementary Table S8. Statistics of predicted protein-coding genes in *W. pigra* genome.

Supplementary Table S9. Statistics of gene functional annotation of leech genomes.

Supplementary Table S10. Statistics of ncRNA annotation of *H. nipponia* genome.

Supplementary Table S11. Statistics of ncRNA annotation of *H. manillensis* genome.

Supplementary Table S12. Statistics of ncRNA annotation of *W. pigra* genome.

Supplementary Table S13. Statistics of secreted protein in leech genomes.

Supplementary Table S14. Copy number of genes related to prey tracking and location.

Supplementary Table S15. Copy number of genes related to bloodsucking characteristics.

Competing interests

The authors declare no competing interests.

Abbreviations

BUSCO: Benchmarking Universal Single-Copy Orthologs; Mb: megabase pairs; KEGG: Kyoto Encyclopedia of Genes and Genomes; GO: gene ontology; NCBI: The National Center for Biotechnology Information; QV: quality value.

Authors' contributions

J. Z. and Q.L. developed the concept of this study; Q.L., J.R. and Z.L. designed the research; X.W. performed genome assembly, gene annotation, and evolutional analysis; T.F. analyzed the transcriptome data; H.S. analyzed the bloodsucking characteristic; X.Y. analyzed the mechanical and visual characteristics; W.Z., C.Q., X.M. J.L., L.L., and K.C. helped with the sample collection; Q.L., Z.L., J.Y., W.C., and J.Y. discussed the results and implications; X.M., L.L., W.X. and J.L. helped with the medicinal applications; X.L. and H.L. performed the statistical analysis; Z.L. drafted the manuscript; S.R. revised the manuscript. All authors read and approved the final manuscript.

ACKNOWLEDGEMENTS

This work was supported by grants from the National Natural Science Foundation of China [grant numbers U20A2051, 31760648, 31860638], the Guangxi Natural Science Foundation [grant number AB18221120], the Guangxi Distinguished Scholars Program [grant number 201835], and the Qihuang High-level Talent Team Training Projects of

Guangxi University of Chinese Medicine: Application of Systems Biology in Chinese Medicine Research [grant number 2021005].

Genomic features	*H. nipponia*	*H. manillensis*	*W. pigra*
Total genome size (Mb)	203.7	157.5	181.4
Number of scaffolds[a]	11+253	13+243	11+183
Scaffold N50 (Mb)	18.5	11.9	16.2
Number of contigs	985	622	437
Contig N50 (Mb)	1.1	2.5	4.1
Number of genes	20,430	18,106	18,540
Repeat sequences	33.7%	25.3%	30.5%

[a] Number of chromosome-level sca□olds and unplaced sca□olds.

Table 1. Summary statistics for the three leech genomes.

Figures

Fig. 1. Genome assembly of the three leech species. A: Hi-C interactive heatmap for genome-wide organization of the three leech species. B: Comparative genomic analysis of the three leech species. Circos diagram depicts genome characteristics. Tracks from the outer to inner circles indicate the following: chromosomes, TE coverage, gene coverage, GC content, gene expression, and syntenic block (*H. nipponia* as reference). C: Demographic history inferred using pairwise sequentially Markovian coalescent analysis.

Fig. 2. Phylogenetic tree and gene family analysis. A: Phylogenetic tree generated using single-copy orthologous genes. Numbers on the nodes are the numbers of expanded (+) and contracted (-) gene families. B: Venn diagram showing the numbers of detected orthologous gene families of four leech species. C: GO analysis of the shared expanded gene families of the two bloodsucking leech species.

Fig. 3. Transcriptome sequencing and analysis of the three leech species. A: Anatomical diagram of a leech. The leech was divided into four parts according to the anatomical structure. B: Heat map of differentially expressed genes (DEGs) in the three leech species. C: Heat map of DEGs at different time points before bloodsucking, during

bloodsucking (5–60 min), and after bloodsucking (24 h) in *H. manillensis*. D & E: GO and KEGG analyses of DEGs at different bloodsucking times in *H. manillensis*. F: Venn diagram showing numbers of DEGs during different bloodsucking times in *H. manillensis*.

Fig. 4. Molecular basis for prey location and tracing by leeches. Schematic diagram for the auditory system (A), visual system (B), and heat-sensitive channel (C) in leeches. On the basis of the genomic and transcriptional data, genes that encode known mechanical or visual receptors were identified in the three leech species. Genes identified in the leeches are labeled using green boxes.

Fig. 5. Molecular basis for the sanguivorous behaviors of leeches. Schematic diagram shows anticoagulation (A), analgesic (B), and anti-inflammatory (C) processes in leeches. The solid lines represent direct interaction, and the dotted lines represent indirect interactions. Genes identified in the leeches are labeled with green boxes.

Reference

1. Jia N, Wang J, Shi W, Du L, Sun Y, Zhan W, et al. Large-Scale Comparative Analyses of Tick Genomes Elucidate Their Genetic Diversity and Vector Capacities. *Cell*. 2020;182(5):1328-40 e13. doi:10.1016/j.cell.2020.07.023.

2. Markwardt and Fritz. Hirudin As Alternative Anticoagulant- A Historical Review. *Semin Thromb Hemost*. 2002;28(5):405-14.

3. Babenko VV, Podgorny OV, Manuvera VA, Kasianov AS, Manolov AI, Grafskaia EN, et al. Draft genome sequences of Hirudo medicinalis and salivary transcriptome of three closely related medicinal leeches. *BMC Genomics*. 2020;21(1):1-16.

4. Simakov O, Marletaz F, Cho SJ, Edsinger-Gonzales E, Havlak P, Hellsten U, et al. Insights into bilaterian evolution from three spiralian genomes. *Nature*. 2013;493(7433):526-531. doi:10.1038/nature11696.

5. Guan D-L, Yang J, Liu Y-K, Li Y, Mi D, Ma L-B, et al. Draft genome of the Asian buffalo leech Hirudinaria manillensis. *Front Genet*. 2020;10:1321.

6. Kvist S, Manzano-Marín A, Carle DD, Trontelj P and Siddall ME. Draft genome of the European medicinal leech Hirudo medicinalis (Annelida, Clitellata, Hirudiniformes) with emphasis on anticoagulants. *Sci Rep*. 2020;10(1):1-11.

7. Tong L, Dai S-X, Kong D-J, Yang P-P, Tong X, Tong X-R, et al. The genome of medicinal leech (Whitmania pigra) and comparative genomic study for exploration of bioactive ingredients. *BMC Genomics*. 2022;23(1):1-13.

8. Rhie A, Walenz BP, Koren S and Phillippy AM. Merqury: reference-free quality, completeness, and phasing assessment for genome assemblies. *Genome Biol*. 2020;21(1):1-27.

9. Lehmkuhl AM, Muthusamy A and Wagenaar DA. Responses to mechanically and visually cued water waves in the nervous system of the medicinal leech. *J Exp Biol*. 2018;221(4):jeb171728.

10. Bricaud O and Collazo A. The transcription factor six1 inhibits neuronal and promotes hair cell fate in the developing zebrafish (Danio rerio) inner ear. *J Neurosci*. 2006;26(41):10438-10451. doi:10.1523/JNEUROSCI.1025-06.2006.

11. Fernald RD. Casting a genetic light on the evolution of eyes. *Science*. 2006;313(5795):1914-1918.

12. Terakita A. The opsins. *Genome Biol*. 2005;6(3):1-9.

13. Lenahan C, Sanghavi R, Huang L and Zhang JH. Rhodopsin: A Potential Biomarker for Neurodegenerative Diseases. *Front Neurosci*. 2020;14:326.

14. Gillette R, Huang R-C, Hatcher N and Moroz LL. Cost-benefit analysis potential in feeding behavior of a predatory snail by integration of hunger, taste, and pain. *Proc Natl Acad Sci*. 2000;97(7):3585-3590.

15. Pitcher T, Lang S and Turner J. A risk-balancing trade off between foraging rewards and predation hazard in a shoaling fish. *Behav Ecol Sociobiol*. 1988;22 3:225-228.

16. Buhren BA, Schrumpf H, Hoff N-P, Bölke E, Hilton S and Gerber PA. Hyaluronidase: from clinical applications to molecular and cellular mechanisms. *Eur J Med Res*. 2016;21(1):1-7.

17. Syed AA and Mehta A. Target specific anticoagulant peptides: a review. *Int J Pept Res Ther*. 2018;24(1):1-12.

18. Muñoz MC, Montes R, Hermida J, Orbe J, Paramo JA and Rocha E. Effect of the administration of recombinant hirudin and/or tissue□plasminogen activator (t□PA) on endotoxin□induced disseminated intravascular coagulation model in rabbits. *Br J Haematol*. 1999;105 (1):117-121.

19. Dunwiddie C, Thornberry N, Bull H, Sardana M, Friedman P, Jacobs J, et al. Antistasin, a leech-derived inhibitor of factor Xa: kinetic analysis of enzyme inhibition and identification of the reactive site. *J Biol Chem*. 1989;264(28):16694-16699.

20. O'Brien LM, Mastri M and Fay PJ. Regulation of factor VIIIa by human activated protein C and protein S: inactivation of cofactor in the intrinsic factor Xase. *Blood*. 2000;95(5):1714-1720.

21. Strand K, Knapp JE, Bhyravbhatla B and Royer Jr WE. Crystal structure of the hemoglobin dodecamer from Lumbricus erythrocruorin: allosteric core of giant annelid respiratory complexes. *J Mol Biol*. 2004;344(1):119-134.

22. Asgari S, Luo Y, Akbari A, Belbin GM, Li X, Harris DN, et al. A positively selected FBN1 missense variant reduces height in Peruvian individuals. *Nature*. 2020;582(7811):234-239. doi:10.1038/s41586-020-2302-0.

23. Li H. Minimap2: pairwise alignment for nucleotide sequences. *Bioinformatics*. 2018;34(18):3094-3100. doi:10.1093/bioinformatics/bty191.

24. Chen S, Zhou Y, Chen Y and Gu J. fastp: an ultra-fast all-in-one FASTQ preprocessor. *Bioinformatics*. 2018;34(17):i884-i890. doi:10.1093/bioinformatics/bty560.

25. Marcais G and Kingsford C. A fast, lock-free approach for efficient parallel counting of occurrences of k-mers. *Bioinformatics*. 2011;27(6):764-770. doi:10.1093/bioinformatics/btr011.

26. Vurture GW, Sedlazeck FJ, Nattestad M, Underwood CJ, Fang H, Gurtowski J, et al. GenomeScope: fast reference-free genome profiling from short reads. *Bioinformatics*. 2017;33(14):2202-2204.

27. Kolmogorov M, Yuan J, Lin Y and Pevzner PA. Assembly of long, error-prone reads using repeat graphs. *Nat Biotechnol*. 2019;37(5):540-546.

28. Vaser R, Sović I, Nagarajan N and Šikić MJGr. Fast and accurate de novo genome assembly from long uncorrected reads. *Genome Res.* 2017;27(5):737-746.

29. Walker BJ, Abeel T, Shea T, Priest M, Abouelliel A, Sakthikumar S, et al. Pilon: an integrated tool for comprehensive microbial variant detection and genome assembly improvement. *PloS One*. 2014;9(11):e112963.

30. Burton JN, Adey A, Patwardhan RP, Qiu R, Kitzman JO and Shendure J. Chromosome-scale scaffolding of de novo genome assemblies based on chromatin interactions. *Nat Biotechnol*. 2013;31(12):1119-1125. doi:10.1038/nbt.2727.

31. Simao FA, Waterhouse RM, Ioannidis P, Kriventseva EV and Zdobnov EM. BUSCO: assessing genome assembly and annotation completeness with single-copy orthologs. *Bioinformatics*. 2015;31(19):3210-3212. doi:10.1093/bioinformatics/btv351.

32. Chen N. Using Repeat Masker to identify repetitive elements in genomic sequences. *Curr Protoc Bioinformatics*. 2004;5(1):4-10. doi:10.1002/0471250953.bi0410s25

33. Stanke M, Diekhans M, Baertsch R and Haussler D. Using native and syntenically mapped cDNA alignments to improve de novo gene finding. *Bioinformatics*. 2008;24(5):637-644. doi:10.1093/bioinformatics/btn013.

34. Korf I. Gene finding in novel genomes. *BMC Bioinformatics*. 2004;5(1):1-9. doi:10.1186/1471-2105-5-59.

35. Haas BJ, Delcher AL, Mount SM, Wortman JR, Smith RK, Jr., Hannick LI, et al. Improving the Arabidopsis genome annotation using maximal transcript alignment assemblies. *Nucleic Acids Res*. 2003;31(19):5654-5666. doi:10.1093/nar/gkg770.

36. She R, Chu JS, Wang K, Pei J and Chen N. GenBlastA: enabling BLAST to identify homologous gene sequences. *Genome Res*. 2009;19(1):143-149. doi:10.1101/gr.082081.108.

37. Birney E, Clamp M and Durbin R. GeneWise and Genomewise. *Genome Res*. 2004;14(5):988-995. doi:10.1101/gr.1865504.

38. Kim D, Paggi JM, Park C, Bennett C and Salzberg SL. Graph-based genome alignment and genotyping with HISAT2 and HISAT-genotype. *Nat Biotechnol*. 2019;37(8):907-915.

39. Pertea M, Pertea GM, Antonescu CM, Chang T-C, Mendell JT and Salzberg SL. StringTie enables improved reconstruction of a transcriptome from RNA-seq reads. *Nat Biotechnol*. 2015;33(3):290-295.

40. Haas BJ, Salzberg SL, Zhu W, Pertea M, Allen JE, Orvis J, et al. Automated eukaryotic gene structure annotation using EVidenceModeler and the Program to Assemble Spliced Alignments. *Genome Biol*. 2008;9(1):1-22. doi:10.1186/gb-2008-9-1-r7.

41. Bairoch A, Apweiler R, Wu CH, Barker WC, Boeckmann B, Ferro S, et al. The universal protein resource (UniProt). *Nucleic Acids Res*. 2005;33(suppl_1):D154-D159.

42. Mao X, Cai T, Olyarchuk JG and Wei L. Automated genome annotation and pathway identification using the KEGG Orthology (KO) as a controlled vocabulary. *Bioinformatics*. 2005;21(19):3787-3793. doi:10.1093/bioinformatics/bti430.

43. Ogata H, Goto S, Sato K, Fujibuchi W, Bono H and Kanehisa M. KEGG: Kyoto Encyclopedia of Genes and Genomes. *Nucleic Acids Res*. 1999;27(1):29-34. doi:10.1093/nar/27.1.29 %J Nucleic Acids Research.

44. Quevillon E, Silventoinen V, Pillai S, Harte N, Mulder N, Apweiler R, et al. InterProScan: protein domains identifier. *Nucleic Acids Res*. 2005;33(suppl_2):W116-W120. doi:10.1093/nar/gki442.

45. Lagesen K, Hallin P, Rodland EA, Staerfeldt HH, Rognes T and Ussery DW. RNAmmer: consistent and rapid annotation of ribosomal RNA genes. *Nucleic Acids Res*. 2007;35 9:3100-8. doi:10.1093/nar/gkm160.

46. Lowe TM and Eddy SR. tRNAscan-SE: A Program for Improved Detection of Transfer RNA Genes in Genomic Sequence. *Nucleic Acids Res*. 1997;25(5):955-964. doi:10.1093/nar/25.5.955 %J Nucleic Acids Research.

47. Nawrocki EP and Eddy SR. Infernal 1.1: 100-fold faster RNA homology searches. *Bioinformatics*. 2013;29(22):2933-2935. doi:10.1093/bioinformatics/btt509.

48. Almagro Armenteros JJ, Tsirigos KD, Sonderby CK, Petersen TN, Winther O, Brunak S, et al. SignalP 5.0 improves signal peptide predictions using deep neural networks. *Nat Biotechnol*. 2019;37(4):420-423. doi:10.1038/s41587-019-0036-z.

49. Kall L, Krogh A and Sonnhammer EL. Advantages of combined transmembrane topology and signal peptide prediction--the Phobius web server. *Nucleic Acids Res*. 2007;35(suppl_2):W429-W432. doi:10.1093/nar/gkm256.

50. Viklund H, Bernsel A, Skwark M and Elofsson A. SPOCTOPUS: a combined predictor of signal peptides and membrane protein topology. *Bioinformatics*. 2008;24(24):2928-2929. doi:10.1093/bioinformatics/btn550.

51. Emms DM and Kelly S. OrthoFinder: phylogenetic orthology inference for comparative genomics. *Genome Biol*. 2019;20(1):1-14. doi:10.1186/s13059-019-1832-y.

52. De Bie T, Cristianini N, Demuth JP and Hahn MW. CAFE: a computational tool for the study of gene family evolution. *Bioinformatics*. 2006;22(10):1269-1271. doi:10.1093/bioinformatics/btl097.

53. Edgar RC. MUSCLE: a multiple sequence alignment method with reduced time and space complexity. BMC *Bioinformatics*. 2004;5(1):1-9. doi:10.1186/1471-2105-5-113.

54. Stamatakis A. RAxML version 8: a tool for phylogenetic analysis and post-analysis of large phylogenies. *Bioinformatics*. 2014;30(9):1312-1313. doi:10.1093/bioinformatics/btu033.

55. Yang Z. PAML 4: phylogenetic analysis by maximum likelihood. *Mol Biol Evol*. 2007;24(8):1586-1591. doi:10.1093/molbev/msm088.

56. Wang Y, Tang H, Debarry JD, Tan X, Li J, Wang X, et al. MCScanX: a toolkit for detection and evolutionary analysis of gene synteny and collinearity. *Nucleic Acids Res*. 2012;40(7):e49. doi:10.1093/nar/gkr1293.

57. Bolger AM, Marc L and Bjoern U. Trimmomatic: a flexible trimmer for Illumina sequence data. *Bioinformatics*. 2014;30(15):2114-2120.

58. Li H, Handsaker B, Wysoker A, Fennell T, Ruan J, Homer N, et al. The Sequence Alignment/Map format and SAMtools. *Bioinformatics*. 2009;25(16):2078-2079.

59. Pertea G and Pertea M. GFF utilities: GffRead and GffCompare. *F1000Research*. 2020;9.

60. Love MI, Huber W and Anders S. Moderated estimation of fold change and dispersion for RNA-seq data with DESeq2. *Genome Biol*. 2014;15(12):1-21.

61. Simon A, Theodor PP and Wolfgang H. HTSeq—a Python framework to work with high-throughput sequencing data. *Bioinformatics*. 2015;31(2):166-169.

62. JH Zheng. Genomes and gene annotations of three leeches. 2022. https://doi.org/10.6084/m9.figshare.20400729. Accessed: 10 Auguest, 2022.

63. Zheng J; Wang X; Feng T; Rehman Su; Yan X; Shan H; Ma X; Zhou W; Xu W; Lu L; Liu J; Luo X; Cui K; Qin C; Chen W; Yu J; Li Z; Ruan J; Liu Q. Supporting data for "Molecular mechanisms underlying hematophagia revealed by comparative analyses of leech genomes" GigaScience Database 2023. http://dx.doi.org/10.5524/102349

64. Zheng J; Wang X; Feng T; Rehman Su; Yan X; Shan H; Ma X; Zhou W; Xu W; Lu L; Liu J; Luo X; Cui K; Qin C; Chen W; Yu J; Li Z; Ruan J; Liu Q. Genomic data of non-bloodsucking leech, Whitmania pigra GigaScience Database 2023. http://dx.doi.org/10.5524/102363

65. Zheng J; Wang X; Feng T; Rehman Su; Yan X; Shan H; Ma X; Zhou W; Xu W; Lu L; Liu J; Luo X; Cui K; Qin C; Chen W; Yu J; Li Z; Ruan J; Liu Q. Genomic data of bloodsucking leech, Hirudo nipponia GigaScience Database 2023. http://dx.doi.org/10.5524/102364

66. Zheng J; Wang X; Feng T; Rehman Su; Yan X; Shan H; Ma X; Zhou W; Xu W; Lu L; Liu J; Luo X; Cui K; Qin C; Chen W; Yu J; Li Z; Ruan J; Liu Q. Genomic data of bloodsucking leech, Hirudinaria manillensis GigaScience Database 2023. http://dx.doi.org/10.5524/102365

Figure1

Click here to access/download;Figure;Figure 1.pdf

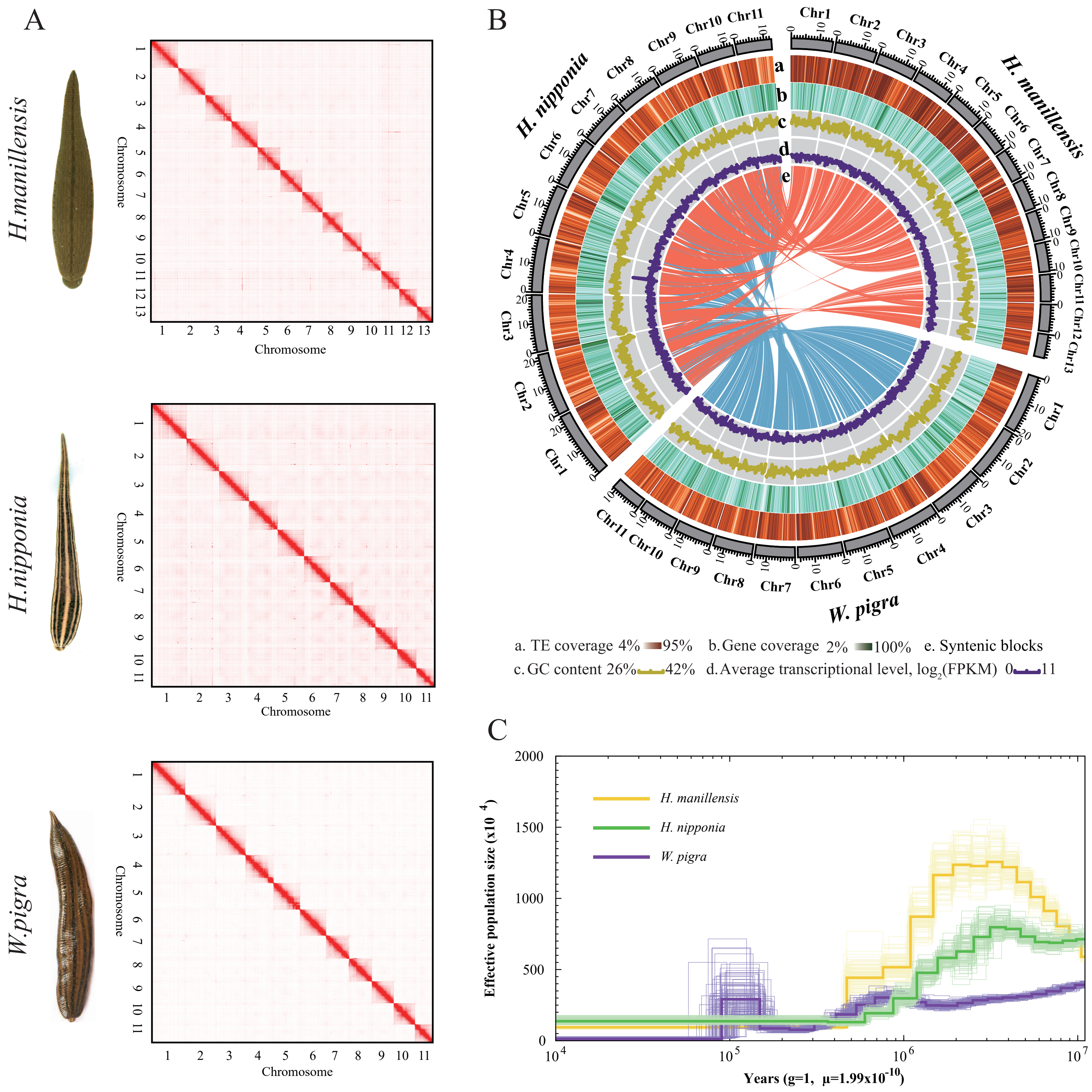

Figure2

Click here to access/download;Figure;Figure 2.pdf

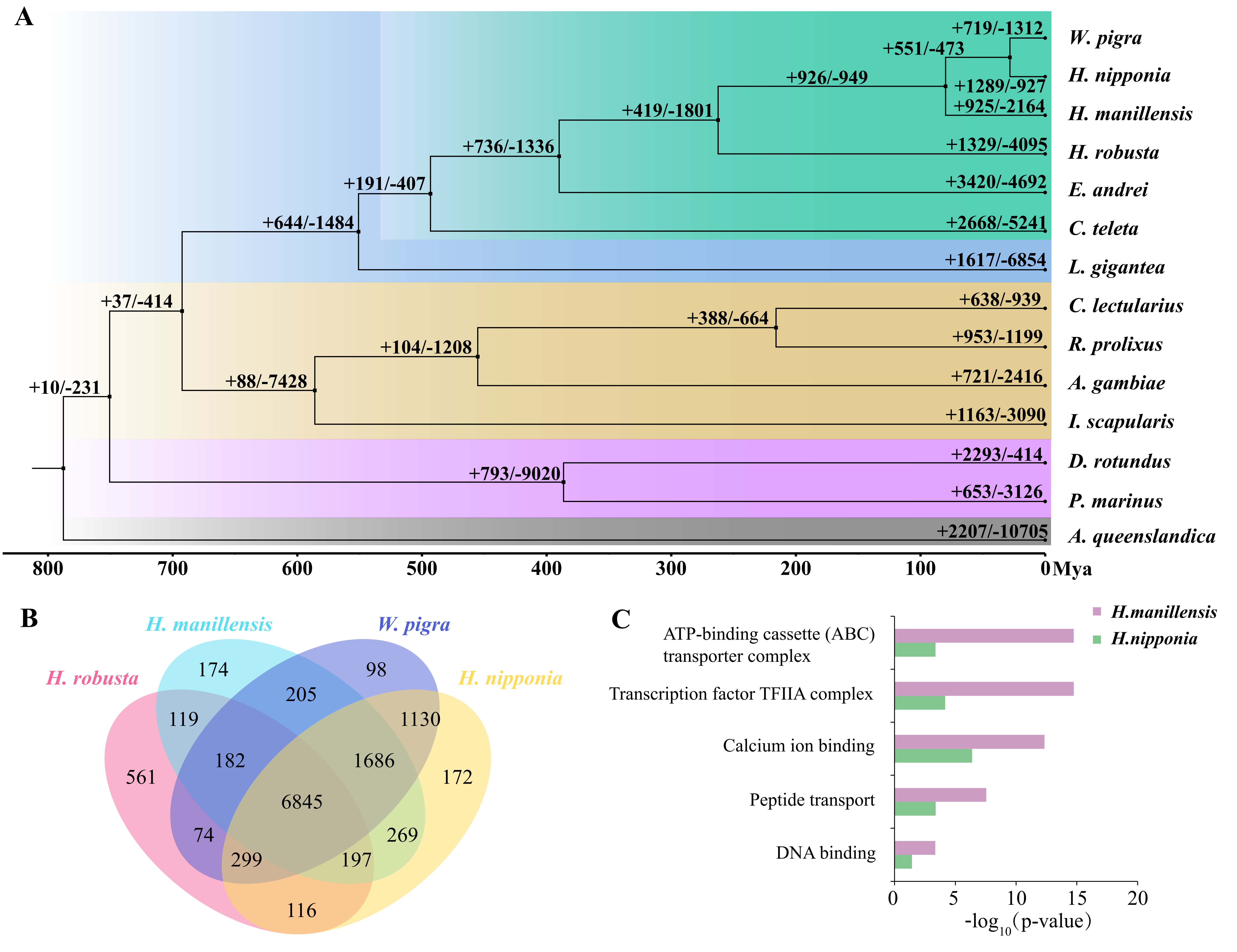

Figure3

Click here to access/download;Figure;Figure 3.pdf

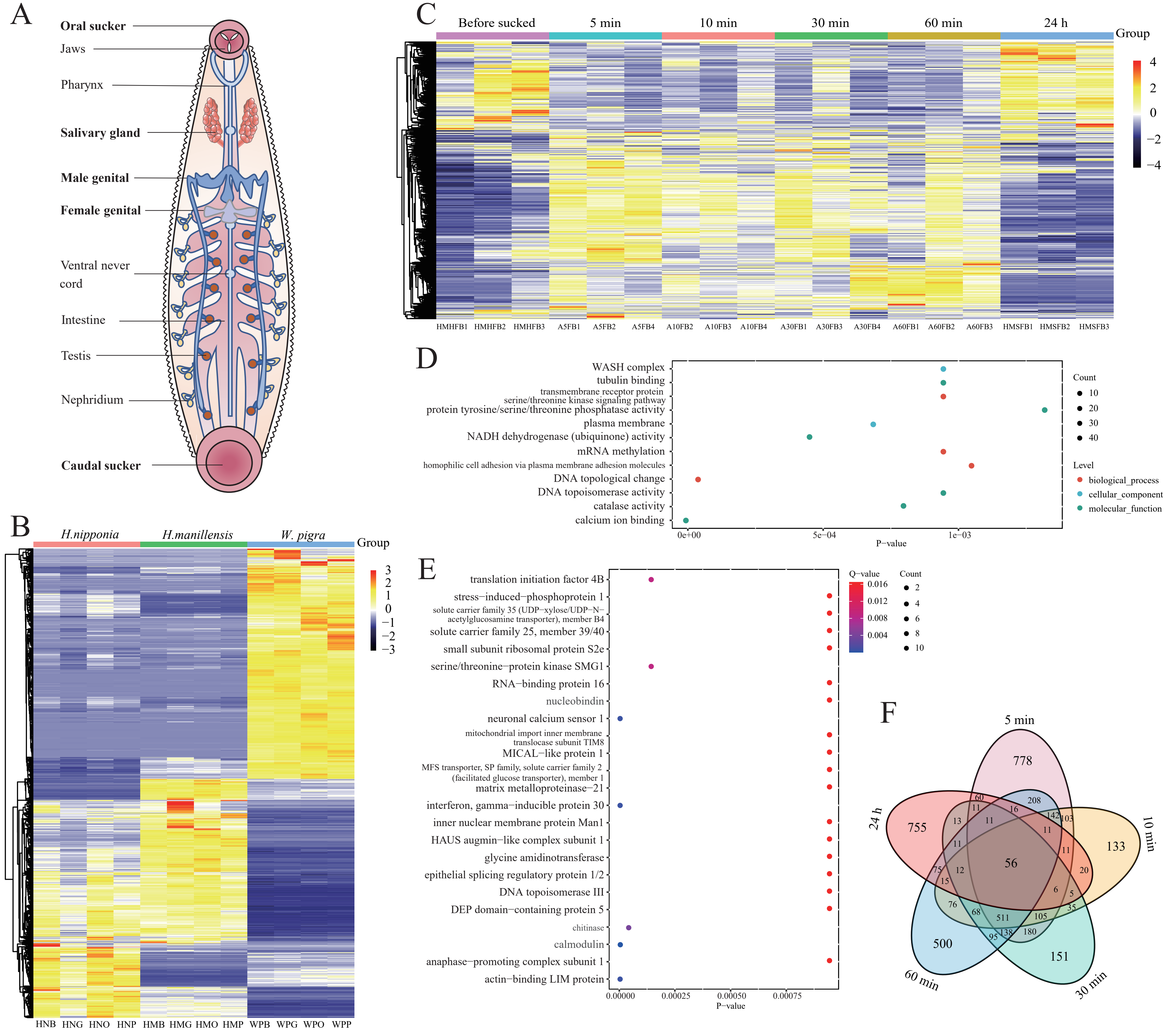

Figure4

Click here to access/download;Figure;Figure 4.pdf

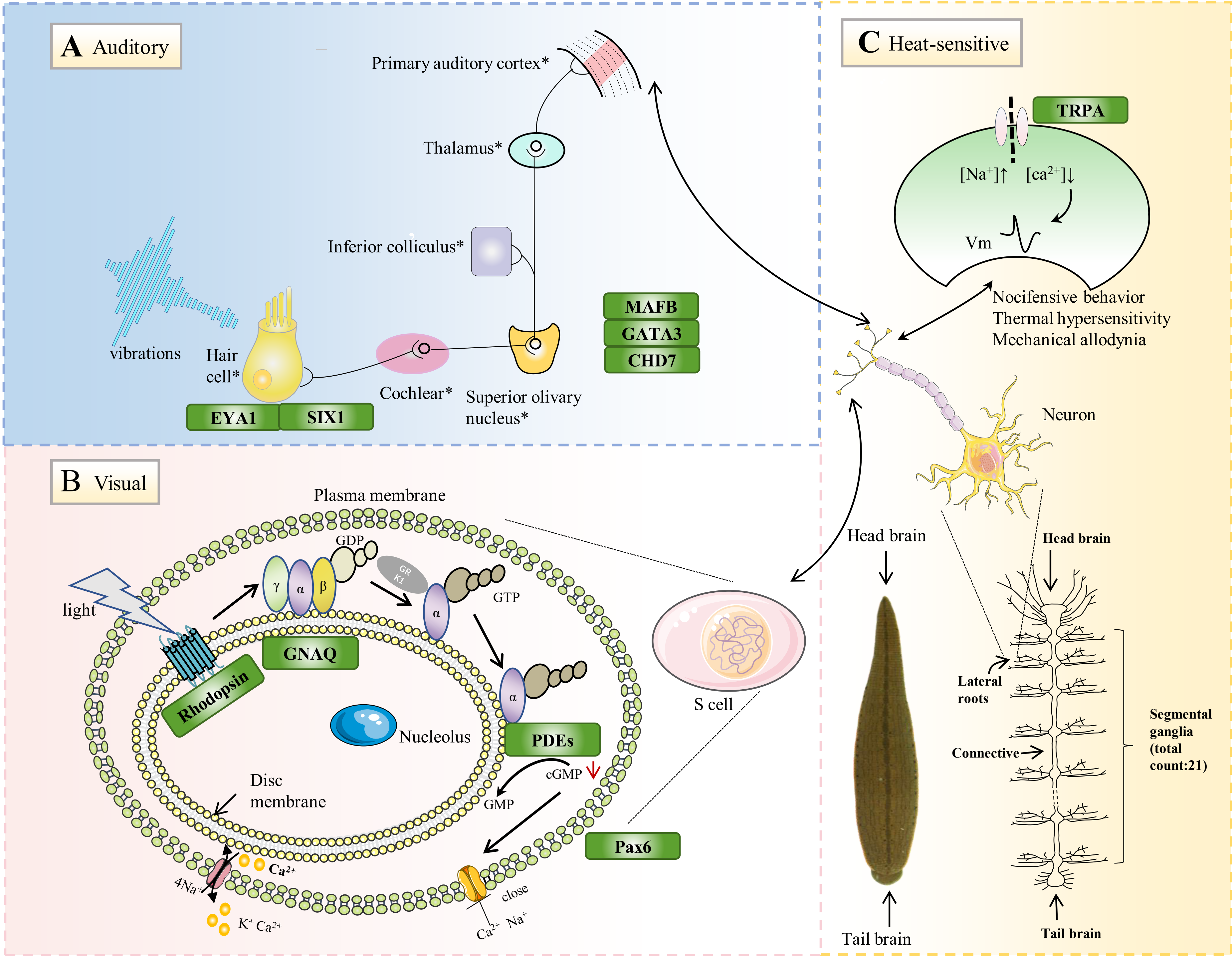

Figure5

Click here to access/download;Figure;Figure 5.pdf

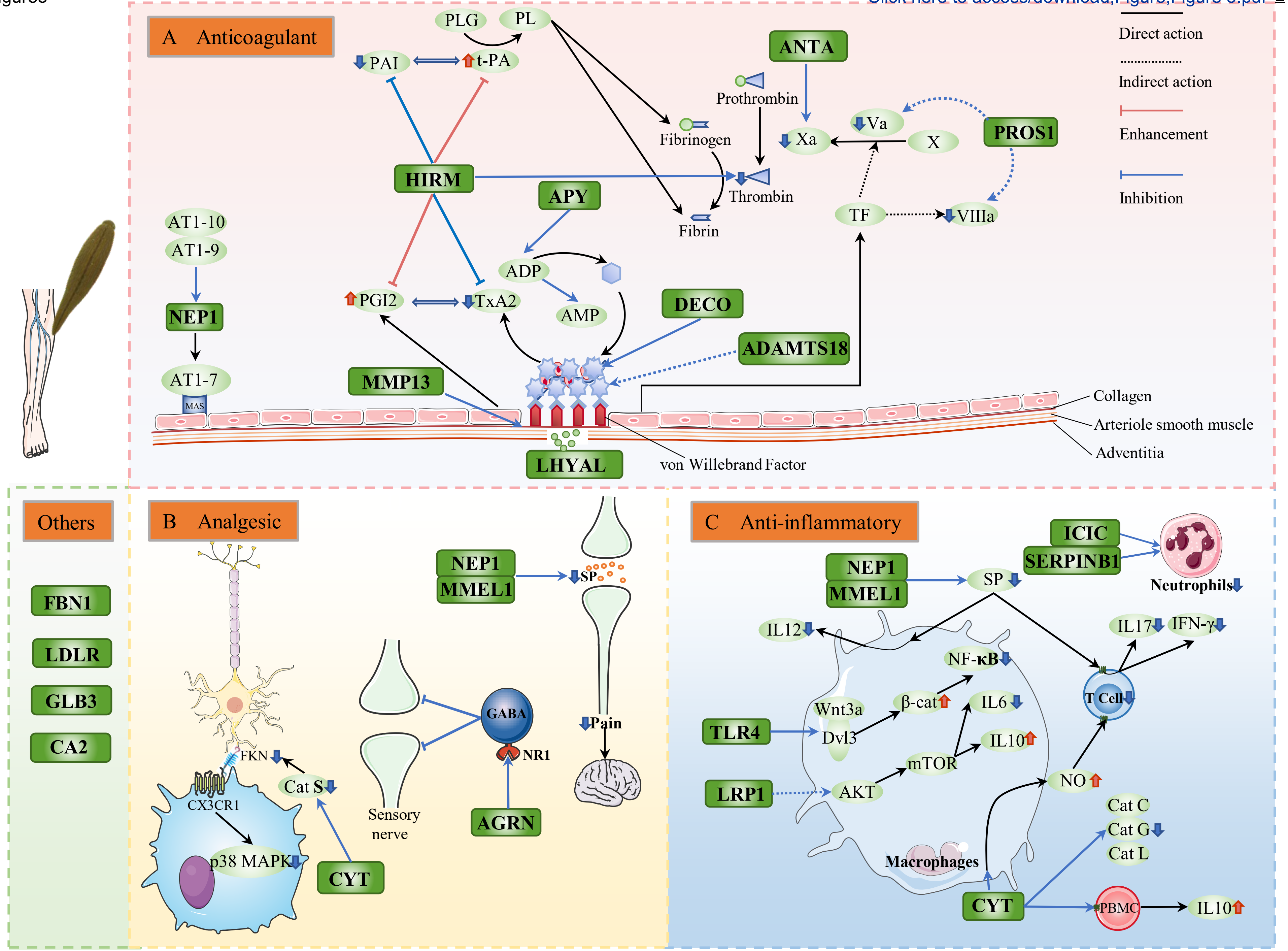

Click here to access/download
Supplementary Material
Supplementary_Materials-r-c.docx

Personal Cover
Click here to access/download;Personal Cover;response-r-c.docx

Dec. 14, 2022

To
The Editorial Office,
GigaScience

Dear editors and reviewers:

Thank you for your kind reply. We are very grateful for the reviewer's comments on our manuscript entitled "Molecular mechanisms underlying hematophagia revealed by comparative analyses of leech genomes" (Manuscript id: GIGA-D-22-00200). Indeed, your comments are very helpful in improving our manuscript. We have carefully studied your comments and tried our best to revise the manuscript to meet your approval. We think we have finished all concerns from reviewers. Here, the revisions are documented in the revised manuscript, response letter, and Supplementary Figs and Tables. The responses to the reviewers' comments are as follows.

Kind regards,

Qingyou Liu

E-mail address: qyliu-gene@gxu.edu.cn

Response to reviewers

Reviewer #1:
This study entitled "Molecular mechanisms underlying hematophagia revealed by comparative analyses of leech genomes" has reported three chromosome-level genomes of leeches. This study has shown population changes of three leeches, and comparative analysis result of copy number of some special gene families has found some clues for divergence between bloodsucking and nonbloodsuking behaviours. The genome materials and findings of this study are indeed significant.
The quality of these three genomes are pretty well. The analysed approaches used in this paper are also well performed.

Response: Thank you very much for your supportive comments. We sincerely appreciate for the valuable comments from you.

Nevertheless, the English language of this paper indeed need to be largely revised by a native speaker. Too much errors were appeared in this paper.

Response: Thank you for your suggestion. The manuscript has been revised by professional editing company ELIXIGEN to improve the writing expression. They are highlighted with red colors and underlines. The editorial certification is listed below.

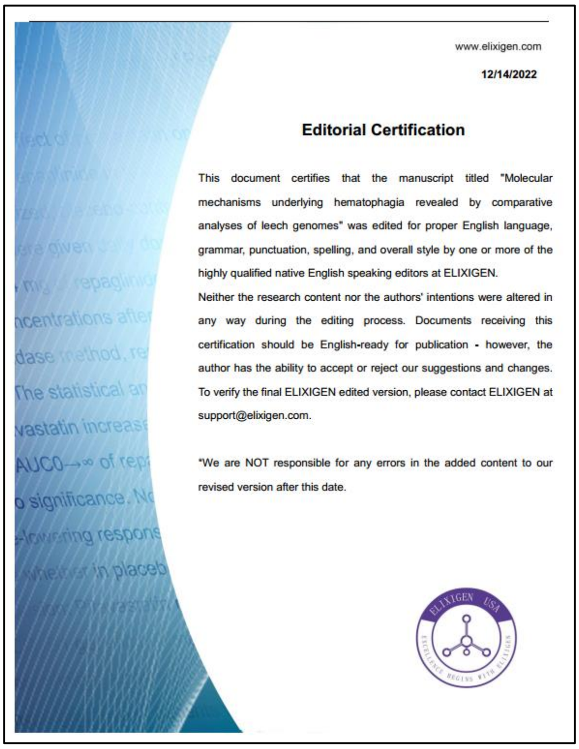

www.elixigen.com

12/14/2022

Editorial Certification

This document certifies that the manuscript titled "Molecular mechanisms underlying hematophagia revealed by comparative analyses of leech genomes" was edited for proper English language, grammar, punctuation, spelling, and overall style by one or more of the highly qualified native English speaking editors at ELIXIGEN.

Neither the research content nor the authors' intentions were altered in any way during the editing process. Documents receiving this certification should be English-ready for publication - however, the author has the ability to accept or reject our suggestions and changes. To verify the final ELIXIGEN edited version, please contact ELIXIGEN at support@elixigen.com.

*We are NOT responsible for any errors in the added content to our revised version after this date.

The detailed version and used library of BUSCO should be described, and the annotation BUSCO number also should be reported.

Response: The BUSCO library we used is metazoa_odb9 (2016-02-13), and the detailed BUSCO number and total BUSCO number are listed in Table S4. Please see below.

Table S4. BUSCO evaluation of the draft assemblies using metazoa_odb9 (2016-02-13) database.

	H. nipponia		*H. manillensis*		*W. pigra*	
	Number	Ratio	Number	Ratio	Number	Ratio
Complete	895	91.5%	888	90.8%	897	91.7%
Fragmented	20	2.0%	22	2.2%	24	2.5%
Missing	63	6.5%	68	7.0%	57	5.8%
Total	978	100.0%	978	100%	978	100.0%

The format of this study should be largely revised, and RRIDs of used software should be added in this manuscript.

Response: We follows the “Data Note” format in gigascience journal to revise our manuscript as editor suggested, and all the available software RRIDs are added.

In figure 1B, the inner aligned lines only contain the alignments between H. nipponia and H. manillensis, and H. nipponia and W. pigra. Why the alignments between H. man and W.pigra are not shown in this figure?

Response: Sorry for lack of the alignments between *H. manillensis* and *W.pigra*. Now we added it. Please check it below in figure1B.

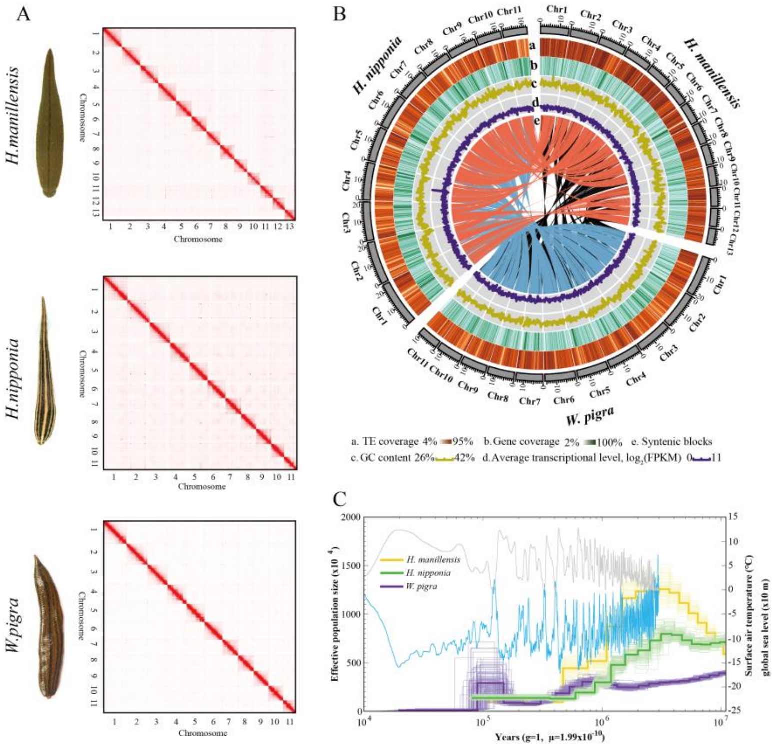

In figure 1C, I suggest authors to add the sea-level and air temperature curves in this figure. The detailed curves you can reference this paper, Whole Genome Sequencing of Chinese White Dolphin (Sousa chinensis) for High-Throughput Screening of Antihypertensive Peptides

Response: That’s a good point. We have added the curves in figure1C. Please check it above.

Some minor suggestions:

"Here we provided three high-quality leech genomes and abundant transcriptomes which illustrated the gene expression dynamics of bloodsucking leeches including anticoagulation, analgesic, and anti-inflammation that would facilitate the understanding at the genetic level and could be crucial for drug candidate prospecting" revised to 'Here we provided three high-quality leech genomes and abundant transcriptomes that illustrated the gene expression dynamics of bloodsucking leeches including anticoagulation, analgesic, and anti-inflammation, which would facilitate the understanding at the genetic level and could be crucial for drug candidate prospecting'.

Response: Thank you for your suggestion. It has been corrected and highlighted with yellow in the manuscript.

For "contained 985, 622, and 437 Mb contigs with N50 contig lengths of 1.1, 2.5, and 4.1 Mb", the decimal places through whole paper should be uniformed. Moreover, this could be an error description for '985, 622 and 437 Mb contigs'. In my opinion, 'Mb' should be removed, there is the number of contigs.

Response: Thanks for pointing this out. The decimal places are now kept to one through the whole paper. "985, 622 and 437" indeed represent the number of contigs, and 'Mb' has been removed.

'and we identified' changed to 'to identify'.

Response: Change made.

"we constructed the gene family" changed to "we constructed gene families".

Response: Change made.

'20430, 18106, and 18540 protein-coding genes' should be '20,430, 18,106, and 18,540 protein-coding genes'

Response: Corrected.

'restored to that' changed to 'restored to those'.

Response: Change made.

'is fundamental to understanding' revised to 'is fundamental to understand'.

Response: Corrected.

Reviewer #2: This manuscript provides some useful genomic and transcriptomic data of three leech species. And the bloodsucking characteristics of leeches are explained to some extent. However, the present version does not meet the publish criteria of GigaScience. Here are my detailed comments which may help to improve the manuscripts before it can be accepted for publication.

Response: Many thanks for your professional comments. We appreciate your time and constructive suggestions, which is very helpful for improving our manuscript. We hope our

revisions in this version could meet your criteria and further receive your replies. Please check our improvements as follows.

1. Correct species classification is crucial for scientific papers. I see the author states that leeches are "arthropods" in the first sentence. It is a trivial but serious mistake, because it has long been accepted by researchers that leeched belong to Annelida. Hence it is reasonable for readers to suspect the validity of taxonomic status of all specimen involved in this manuscript. I strongly suggest that the authors firstly taking both a morphological and a molecular identification of all their leech specimens.

Response: We feel very sorry for that mistake and definitely agree with your opinion. Now we have corrected and changed “arthropods” to “annelids”.

2. As mentioned in the title as well as the whole text, the main interest of this manuscript focus on the molecular mechanisms of hematophagous behavior of leech. However, the most characteristic anticoagulants of leech such as hirudin is poorly discussed, e.g. gene structure, sequence specificity. On the contrary, FBN1 and GLB3, which is mainly discussed in the manuscript, is farfetched to explain the blood sucking habit.

Response: Thank you for pointing this out. Following your suggestions, we performed analysis on hirudin genes about gene structure, sequence specificity and gene expression pattern. Three hirudin genes were identified in *H. manillensis*, and one is identified in both *H. nipponia* and *W. Pigra*. The intron regions of HN_hirudin and WP_hirudin are inserted with transposable elements (TEs), leading to longer length than the three HM_hirudin genes without TE insertions (Fig.S11A below). Multiple sequence alignments show that the cysteine pattern of the hirudin is more conserved than the tail but less conserved than the beginning of the alignment (Fig.S11B below). Finally, we find that hirudin genes mainly express in the oral suckers of the three leeches, and the expression of HM_hiridin1 is comparatively increased at the time point of 30 minutes (Fig.S11C), which is consistent with the time of physiological coagulation.

Fig. S11. Analysis of hirudin genes in the three leeches.

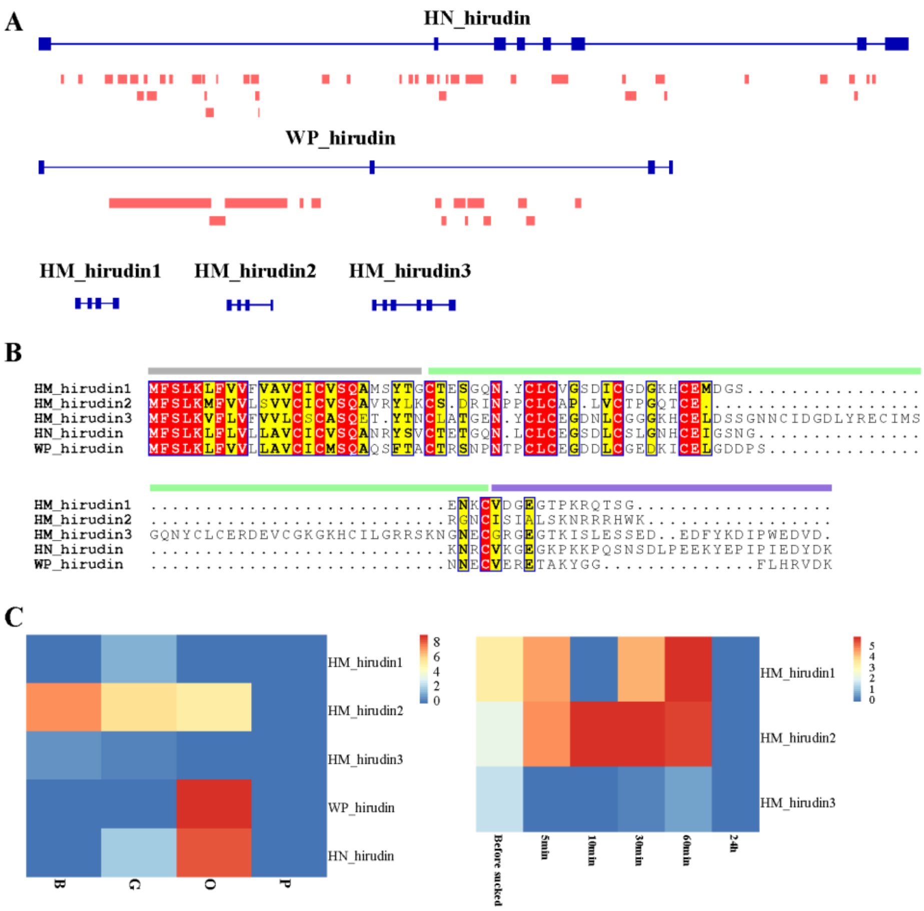

We noticed that HM_hirudin3 shows “redundant” sequence in the region of the cysteine pattern. We checked the alignment of the transcriptome data and confirmed the authenticity of the “redundant” sequence (figure below). It suggests that the cysteine pattern is presumably highly plastic.

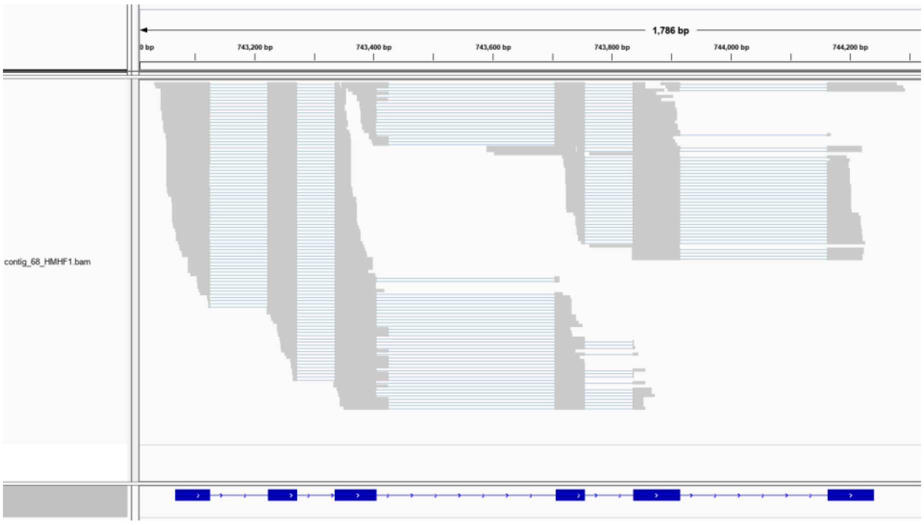

We thus described our revisions in this main text as follows:

“The gene structure and copy number of the well-known anticoagulant HIRM1 (hirudin) were different in the three leeches (Supplementary Fig. 11). The gene lengths of HN_hirudin and

WP_hirudin were apparently longer than the three HM_hirudin copies, and this was mainly attributable to TE insertion into intron regions (Supplementary Fig. 11A). We performed multiple sequence alignment for five HIRM1 genes and found that the cysteine pattern of hirudin [7] is more conserved than the tail but less conserved than the beginning of the alignment (Supplementary Fig. 11B). This indicates that the cysteine pattern may have the potential to be plastic. HIRM1 was mainly expressed in the oral suckers of the three leech species, and the expression of HM_hiridin1 comparatively increased at 30 min (Supplementary Fig. 11C), which is consistent with the time of physiological coagulation."

3. The authors mentioned in the Introduction "Currently, a few leech species genomic data including one non-blood sucking leech (Helobdella robusta) and low coverage genome sequence data of two lineages Amynthas cortices have been published [3-6],…". Actually, besides Helobdella robusta there have been four leech species deposited in the GenBank: Hirudo medicinalis (accession numbers: GCA_011800805.1, GCA_903470615.1); Hirudo verbana (GCA_020137395.1); Hirudinaria manillensis (ASM1534595v1); Whitmania pigra (GCA_021613335.1, GCA_021650995.1). The latter two overlapped with the species used in this manuscript. It is clear that the authors noticed the GenBank data since H. medicinalis and H. manillensis are listed in the Table S1. It is not clear, however, that why they drop two more famous species H. verbana and W. pigra, but instead using a distantly related species Helobdella robusta and even earthworms?

Response: We apologize for the incomplete collection of genomic information. We change the sentence to "the genomic data of several leech species have been published" and add all the genomic information of the mentioned species into Table S1 as well as the available references into the main content.

Table S1. Comparison of genome assembly to other publicized leech genomes.

Species	Total genome size (Mb)	Scaffolds Number	Scaffold N50	Number of genes
H. nipponia	203.7	11+253	18.5 MB	20,430
H. manillensis	157.5	13+243	11.9 MB	18,106
W. Pigra	181.4	11+183	16.2 MB	18,540
H. medicinalis (Genbank accession numbers:GCA_903470615.1)	177.0	19,929	50.4 KB	35,166
H. medicinalis (GCA_011800805.1)	187.6	14,042	97.8 KB	14,596
H. manillensis (ASM1534595v1)	151.8	467	2.3 MB	17,865

H. verbana (GCA_020137395.1)	235.0	59,817	8.4 KB	-
W. pigra (GCA_021613335.1)	178.8	483	2.0 MB	-
W. pigra (GCA_021650995.1)	177	10,050	728.0 KB	26,743
H. robusta (GCA_000326865.1)	228	1,991	3.6MB	23,400

Additional reference:

Tong, Lei, et al. "The genome of medicinal leech (Whitmania pigra) and comparative genomic study for exploration of bioactive ingredients." *BMC Genomics* 23.1 (2022): 1-13.

4.There are obvious differences in genome size between different species and different papers of the same species. It is suggested that the authors explaining the reason or examine whether there is DNA contamination from microorganisms or other species. For example, the H. nipponia has a much larger genome than the other species in Hirudinidae. Is there potential contamination by bacteria genome?

Response: In our analysis, we removed these contigs that covered more than 50% of the bacterial genome sequences deposited in NCBI (see the method “Genome assembly and assessment”). Thus, bacterial contamination likely has little effect on genome size in this study. In recent studies on large genomes, such as african lungfish[1] (~40Gb) and Mexican axolotl[2] (~32Gb), TEs mainly contribute to their huge genomes. In our leech genomes, the maximum difference in the lengths of the TE sequences is 28.8Mb (accounts for 62.3% of the difference of genome sizes) between *H. nipponia*, and *H. manillensis*, suggesting the TE contents maybe a major driver to the larger leech genome. Consistent with the speculation, TEs are responsible for the intron expansion in hirudin genes of *H. nipponia* and *W. pigra* compared to them of *H. manillensis* (Question 3).

For the same species, it is known that the genome size and contig N50 could be improved by using more sequencing reads. For example, we provided ~48 Gb compared with ~12Gb in Guan et al.[3] for *H. manillensis*, and we obtained more ~5.7Mb genome sequences as well as longer contig N50 (2.5Mb vs 2.3Mb). Besides, long-read sequencing technology are usually used to resolve many repeats longer than the second-sequencing reads, leading to more complete assembly results. For example, we assembled larger genome (181.4Mb vs 177Mb) for *W. pigra* and more repetitive contents (30.5% vs 23%) than Tong et al[4]. In addition, the different assembling strategies applied in different papers presumably partly contribute to the difference of genome sizes for the same species.

[1] Wang, Kun, et al. "African lungfish genome sheds light on the vertebrate water-to-land transition." *Cell* 184.5 (2021): 1362-1376.

[2] Nowoshilow, Sergej, et al. "The axolotl genome and the evolution of key tissue formation regulators." *Nature* 554.7690 (2018): 50-55.

[3] Guan, De-Long, et al. "Draft genome of the Asian buffalo leech Hirudinaria manillensis." *Front. Genet.* 10 (2020): 1321.

[4] Tong, Lei, et al. "The genome of medicinal leech (Whitmania pigra) and comparative genomic study for exploration of bioactive ingredients." *BMC Genomics* 23.1 (2022): 1-13.

5.The number of chromosome of leech varies between species. The HI-C analysis in this work was based on an assumed number of these three species, which could lead to a specious result. It is recommended that the author provide verification on the chromosome number of those new reported genomes before they draw conclusions from the HI-C analysis.

Response: We apologize that the software used for contig clustering was incorrectly written as Lachesis (the corresponding reference is about 3D-DNA software), which is in fact 3D-DNA. Just as you said, Lachesis indeed was based on assumed chromosome number. 3D-DNA automatically cluster the contigs without setting chromosome numbers. We tried to identify their karyotypes using experimental method but failed previously, so we describe the the HiC clustering results as “pseudo-chromosomes”.
