# Supplementary material for: Molecular mechanisms underlying hematophagia revealed by comparative analyses of leech genomes
Source: Gigascience. 2023 Apr 11;12:giad023. doi: 10.1093/gigascience/giad023 (PMC10087013; doi:10.1093/gigascience/giad023)
Supplement: giad023_Supplemental_File [file giad023_supplemental_file.docx]

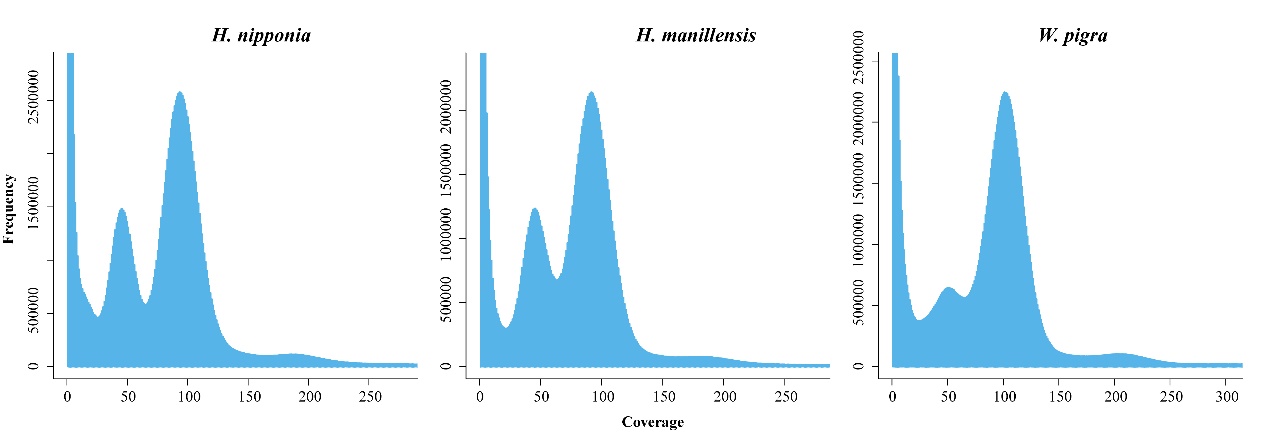


**Fig. S1. The 17-mer distributions of three leech genomes.** Estimated genome size was 206 Mb, 155 Mb, and 172 Mb for *H. nipponia*, *H. manillensis*, and *W. pigra*, respectively.


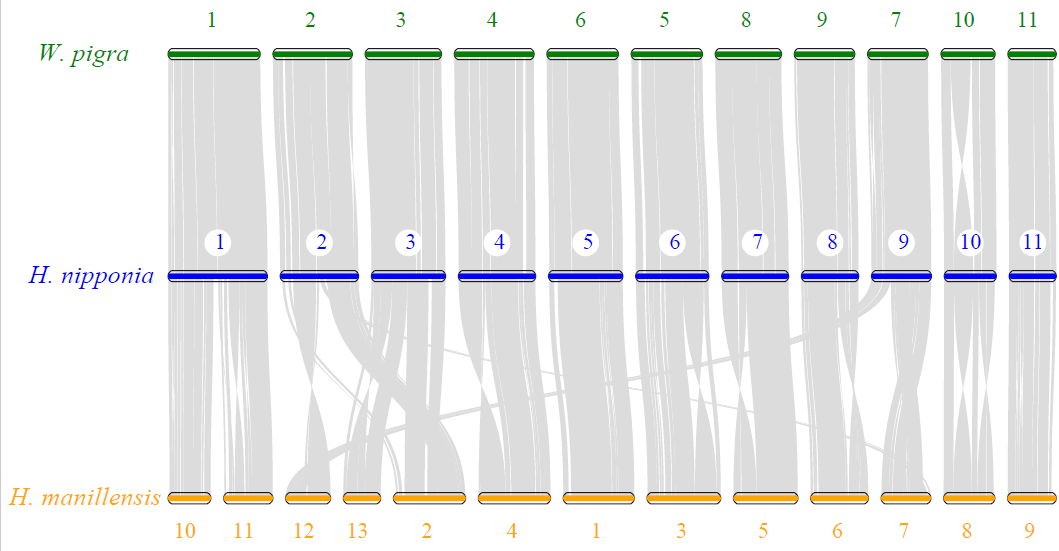


**Fig. S2. Interspecific synteny analysis of three leech genomes.** The number for each leech genome indicates chromosome number.


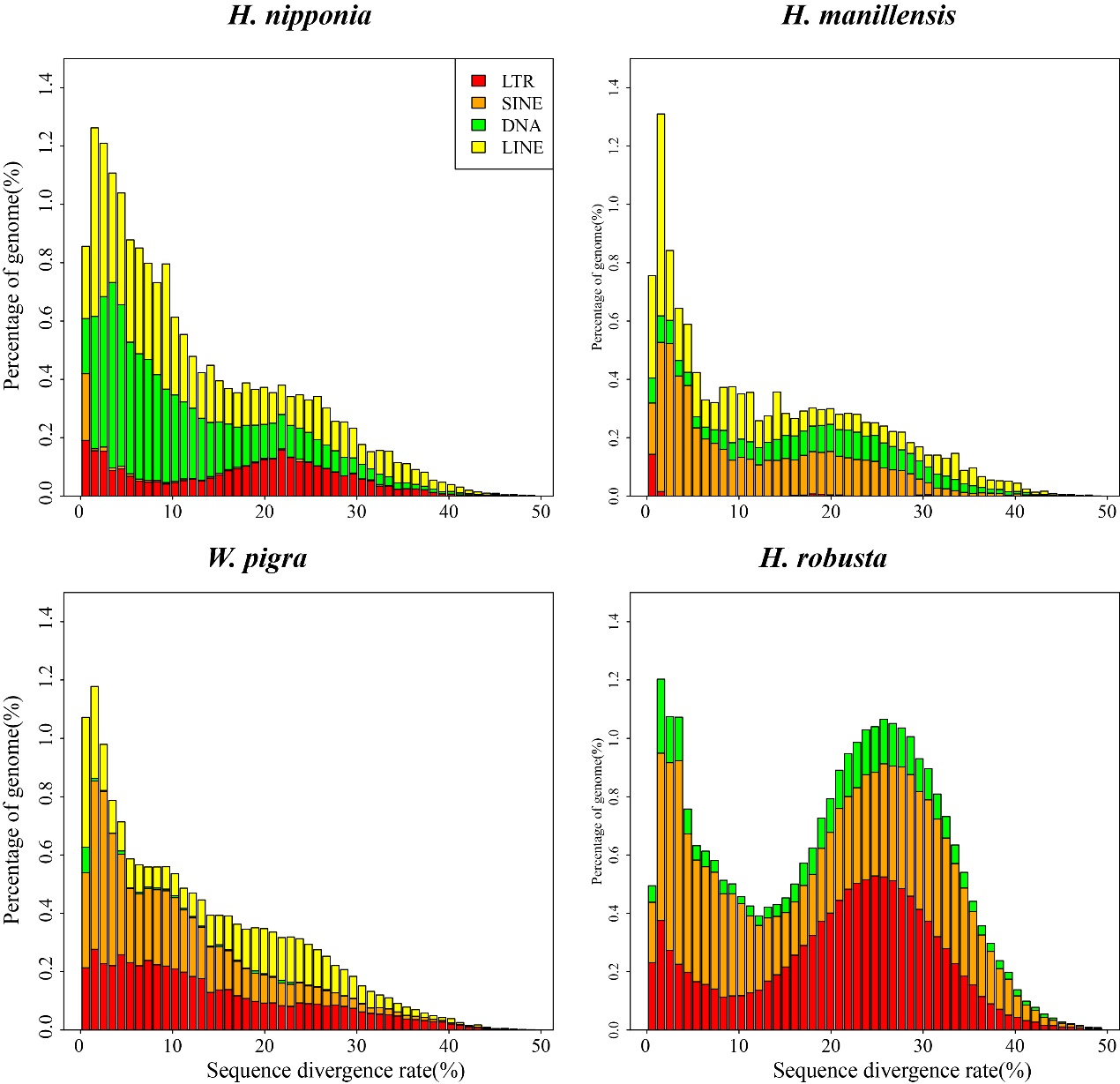


**Fig. 3. TE sequence divergences of four leech genomes.** DNA transposons and long interspersed nuclear elements (LINEs) comprise most of the repeats in *H. nipponia/H. manillensis/W. pigra* genomes, which is quite different from that in *H. robusta.*


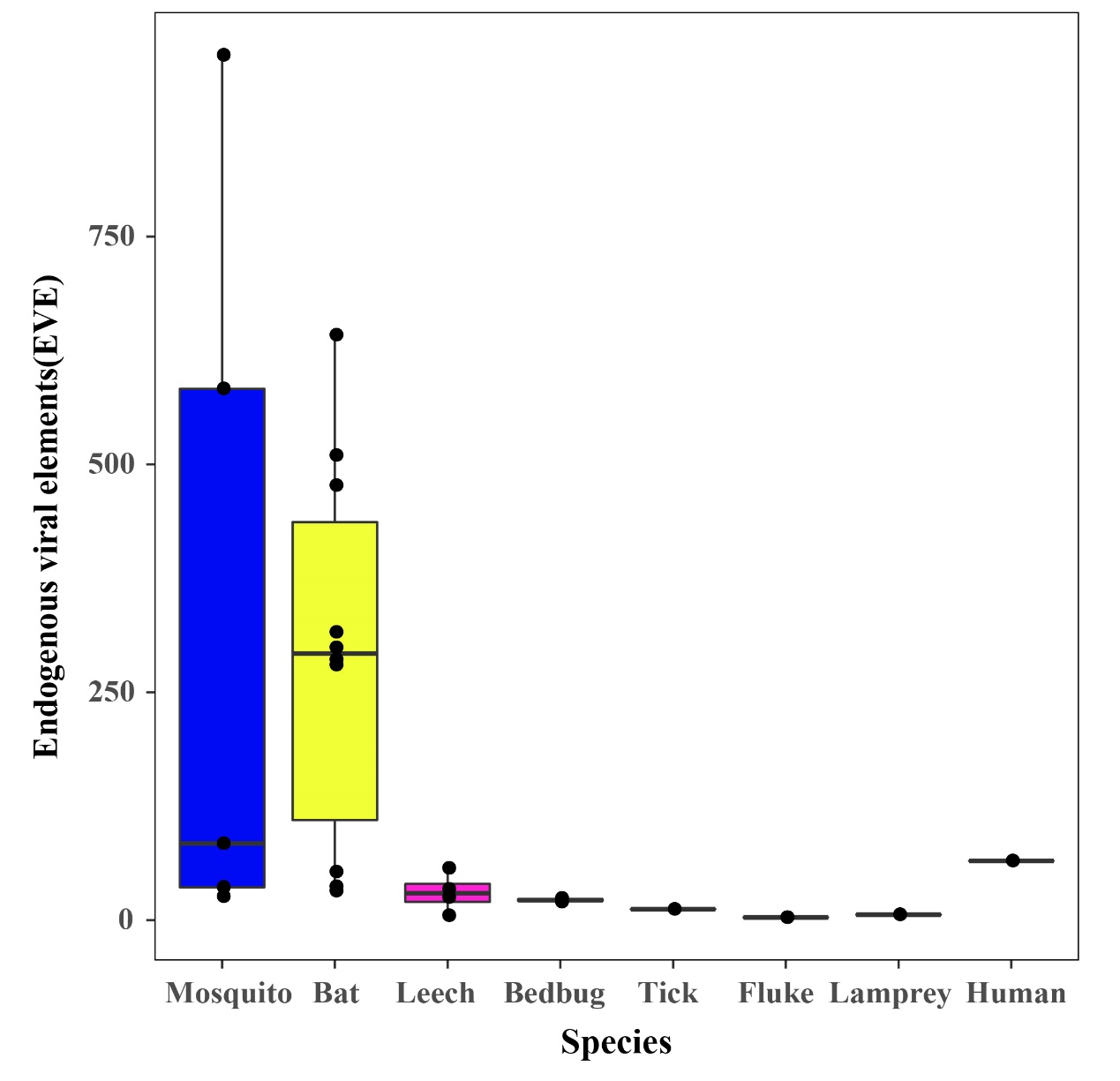


**Fig. S4. The number of endogenous viral elements (EVEs) in the genomes of humans and several hematophagous species.** The EVE number of leech is lower than those of mosquito and bat.


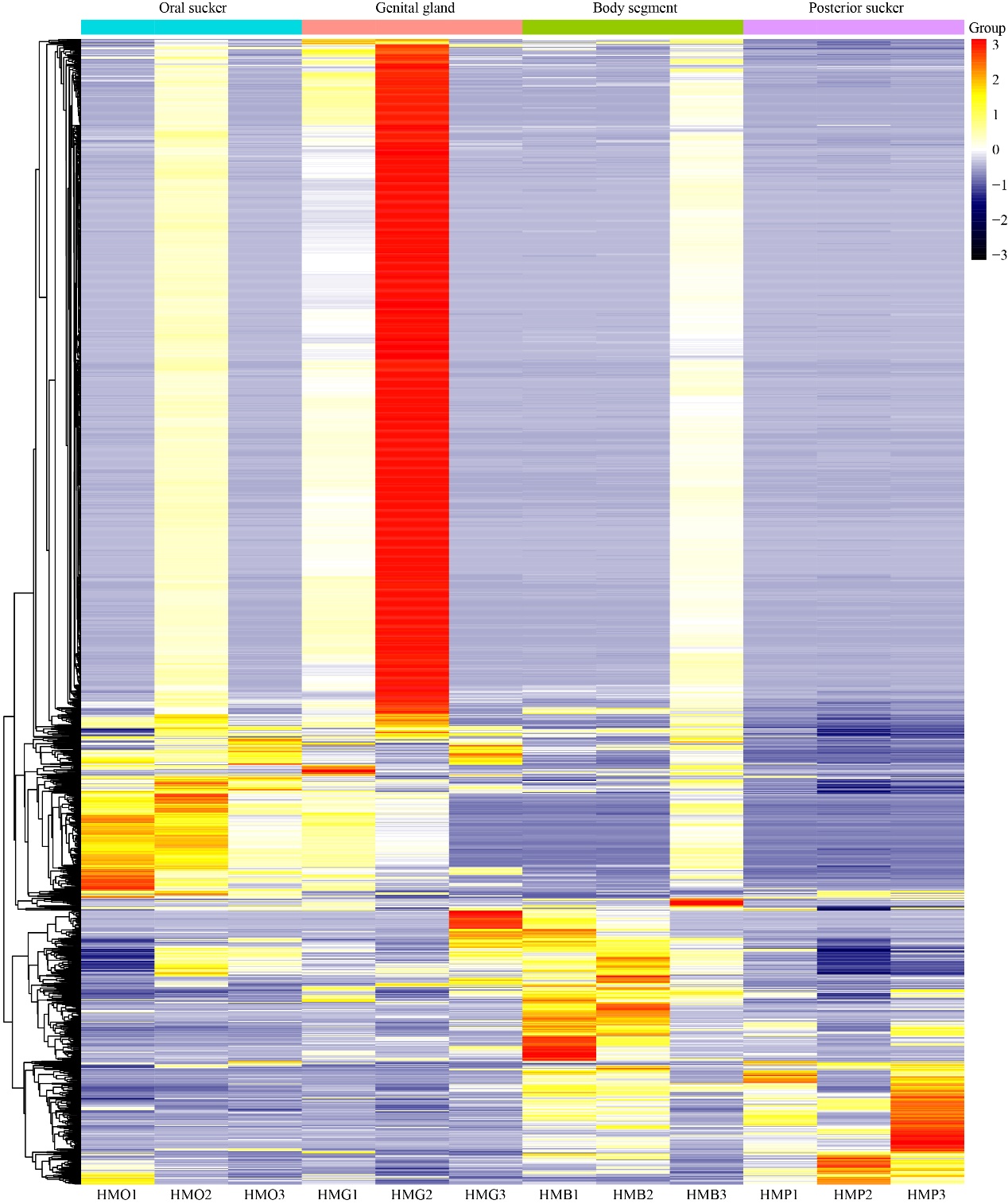


**Fig. S5.** **Gene expression in different body parts of *H. manillensis*.** Each tissue had three replicates.


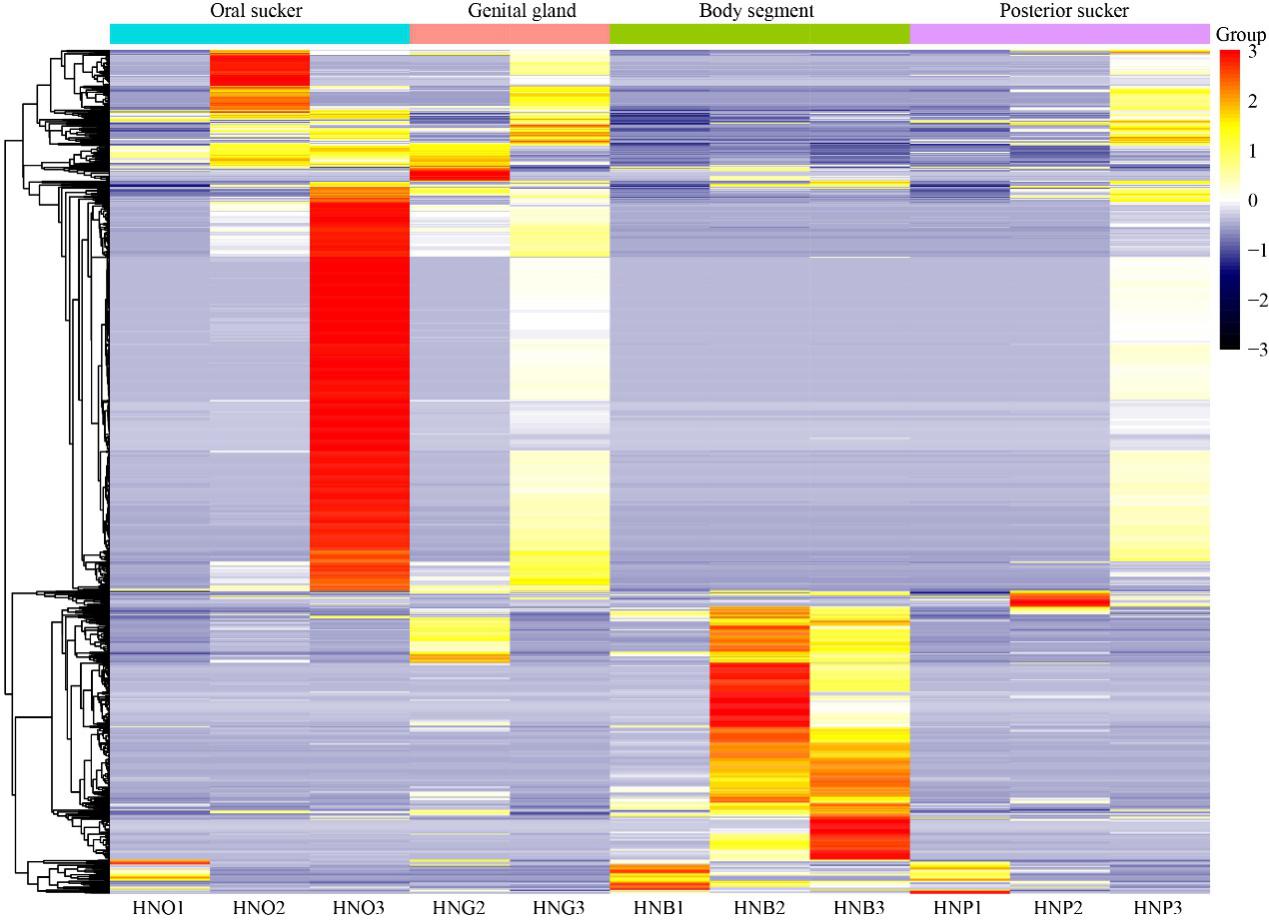


**Fig. S6.** **Gene expression in different body parts of *H. nipponia*.** Each tissue had three replicates.


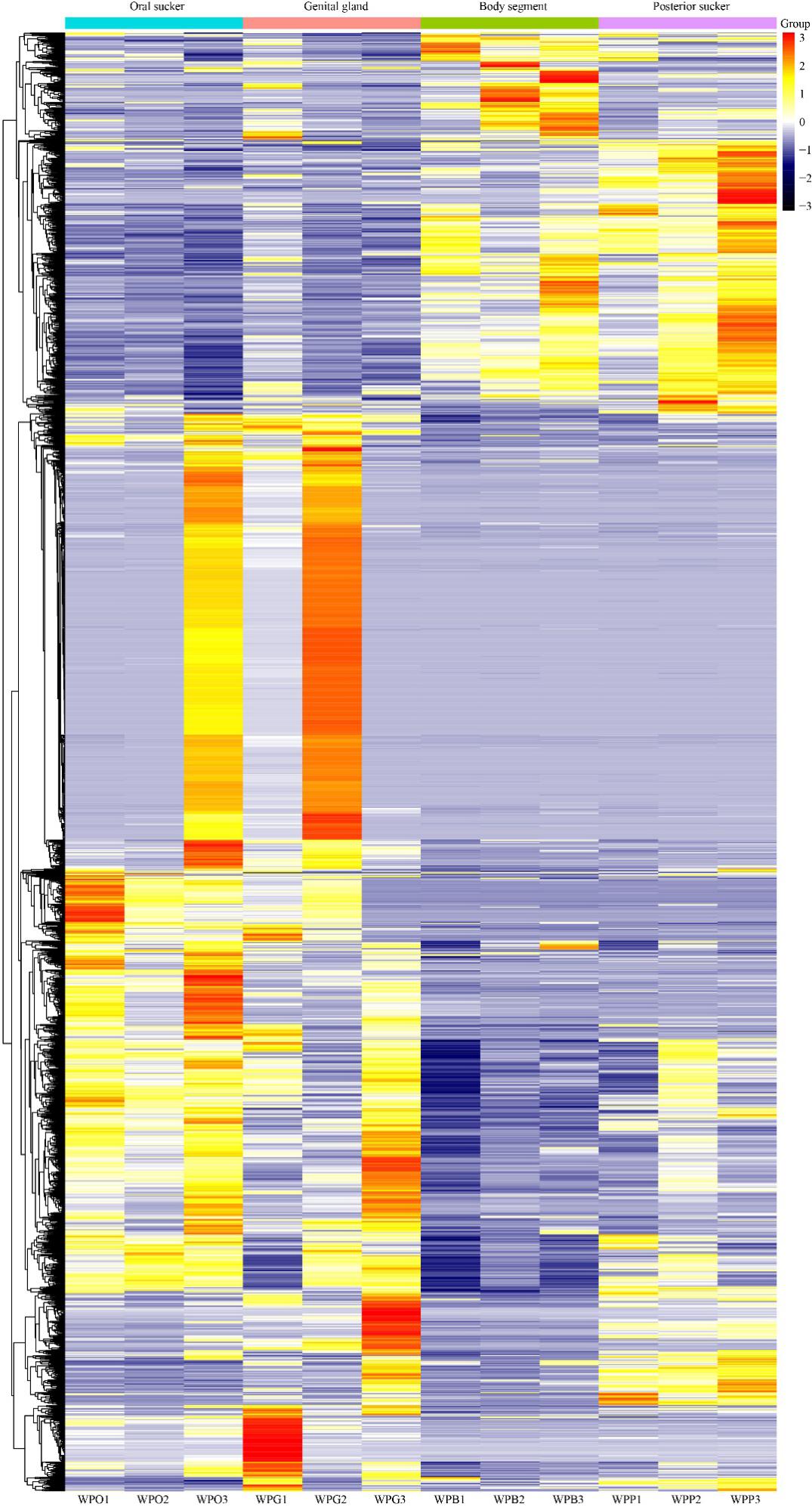


**Fig. S7. Gene expression in different body parts of *W. pigra*.** Each tissue had three replicates.


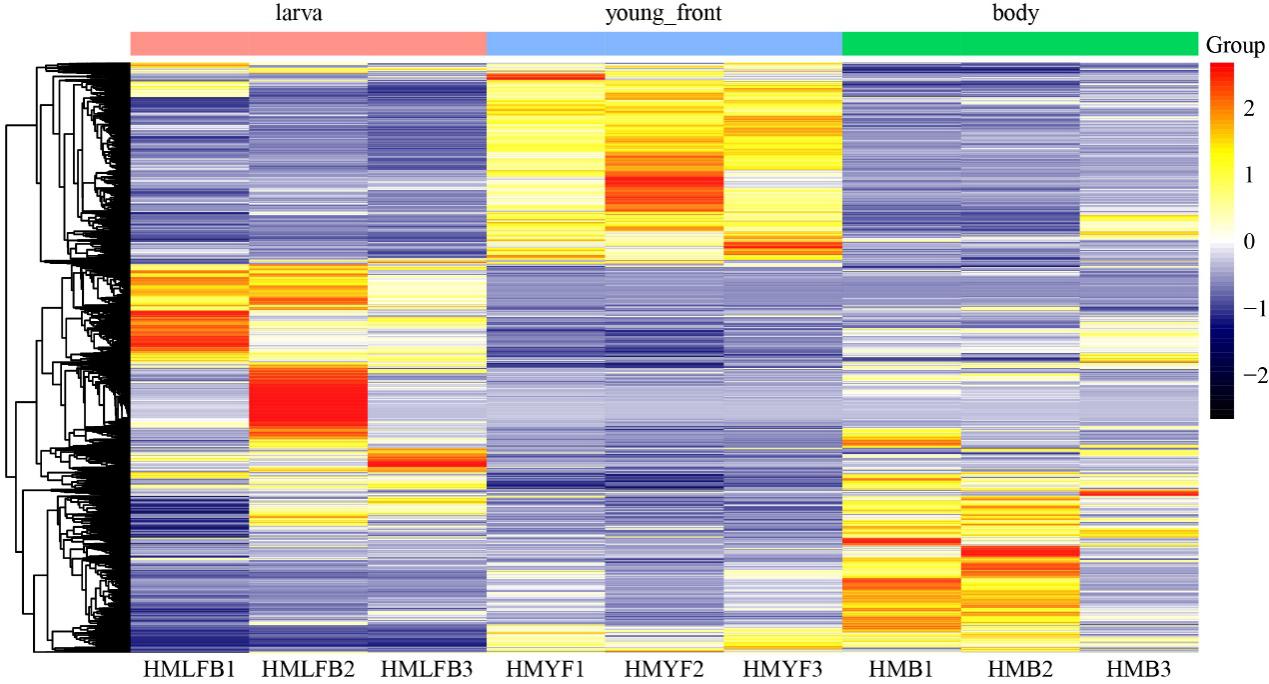


**Fig. S8.** **Gene expression in different developmental stages of *H. manillensis*.** Each tissue had three replicates.


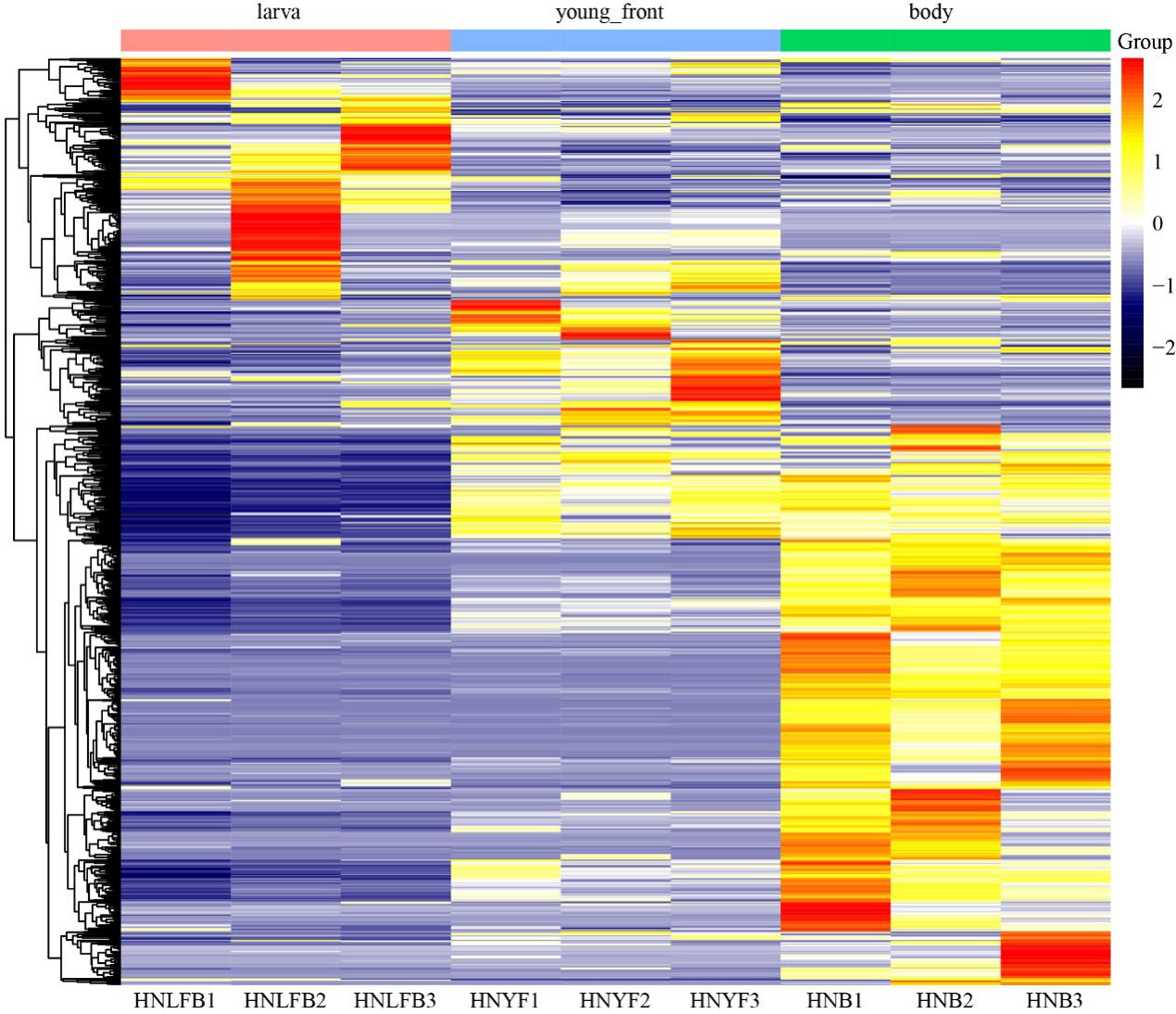


**Fig. S9.** **Gene expression in different developmental stages of *H. nipponia*.** Each tissue had three replicates.


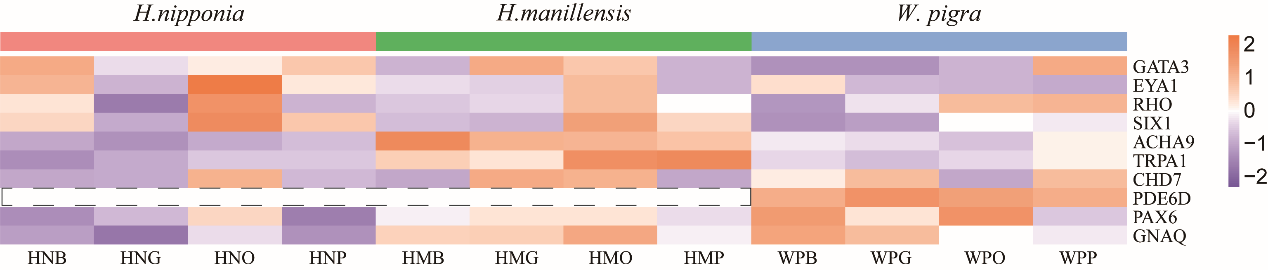


**Fig. S10.** **Expression of genes related to prey location in three leech species.** We merged the gene expressions of all copies for comparison of different leech species. The dotted line represents *PDE6D* absent in two bloodsucking leech species.


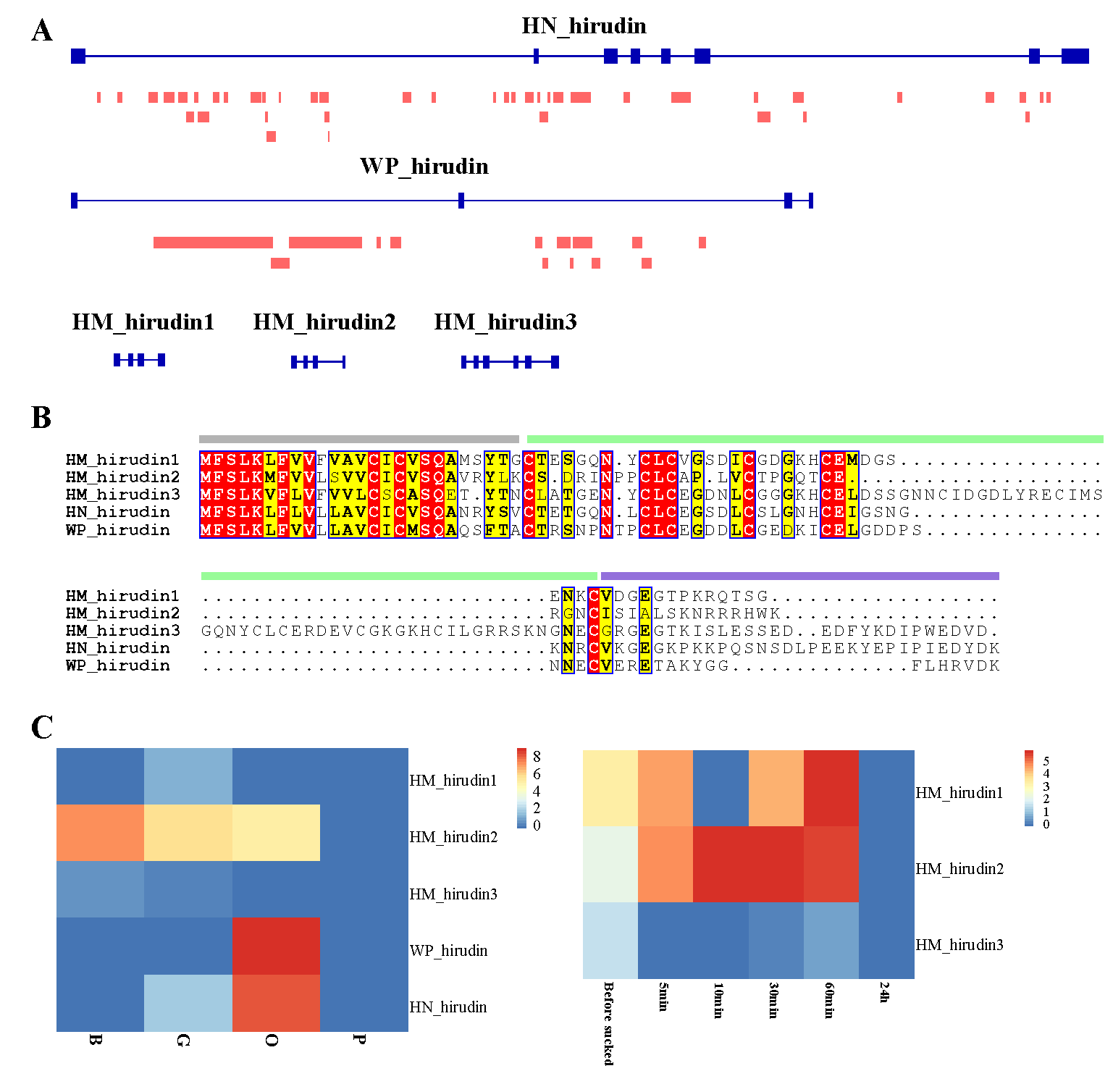


**Fig. S11.** **Analysis of hirudin genes in three leech species.** (A) Gene structure of hirudin. HN represents *H. nipponia*, WP represents *W. pigra*, and HM represents *H. manillensis.* (B) Multiple sequence alignments of the hirudin genes. (C) Expressions of hirudin genes in the three leech species. “B,” “G,” “O,” and “P” indicate “body,” “genital gland,” “oral sucker,” and “posterior sucker,” respectively.


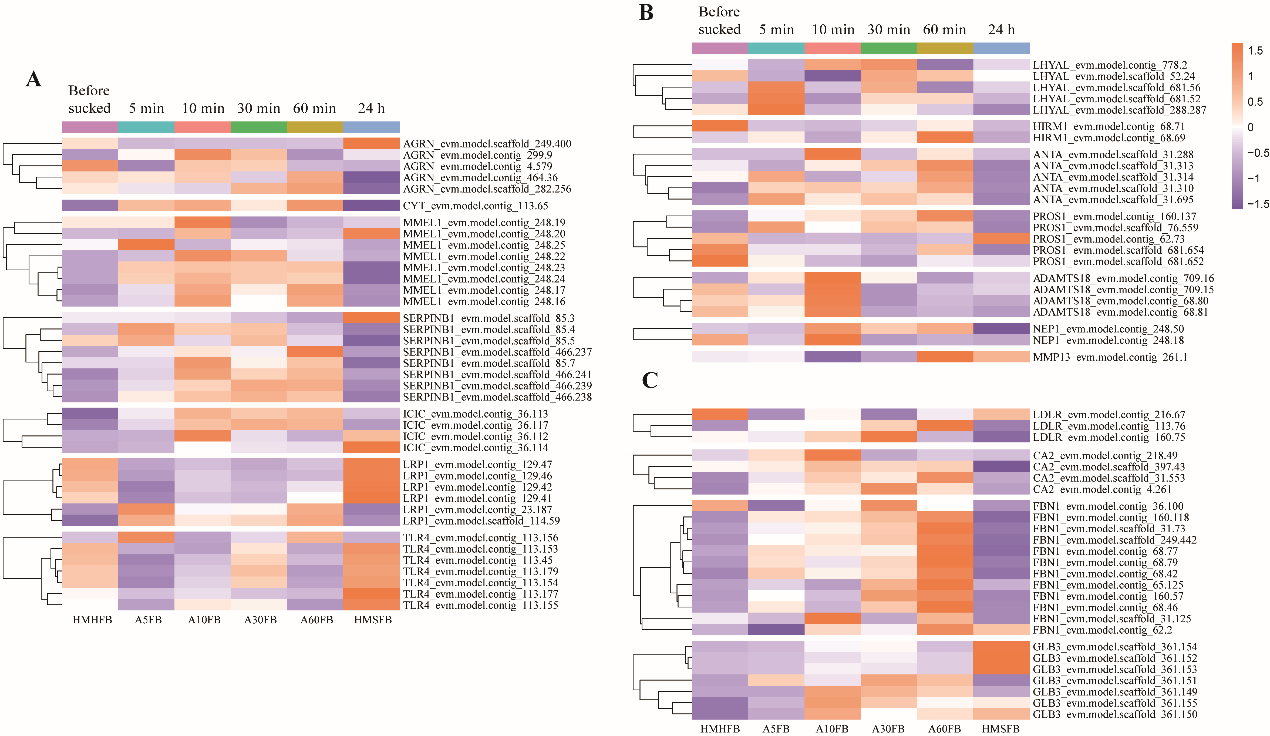


**Fig. S12.** **Expression of bloodsucking-related genes in *H. manillensis*.** (**A**) Gene expressions in analgesic and anti-inflammatory processes. (**B**) Gene expressions in anticoagulation process. C: Other bloodsucking-related gene expressions.


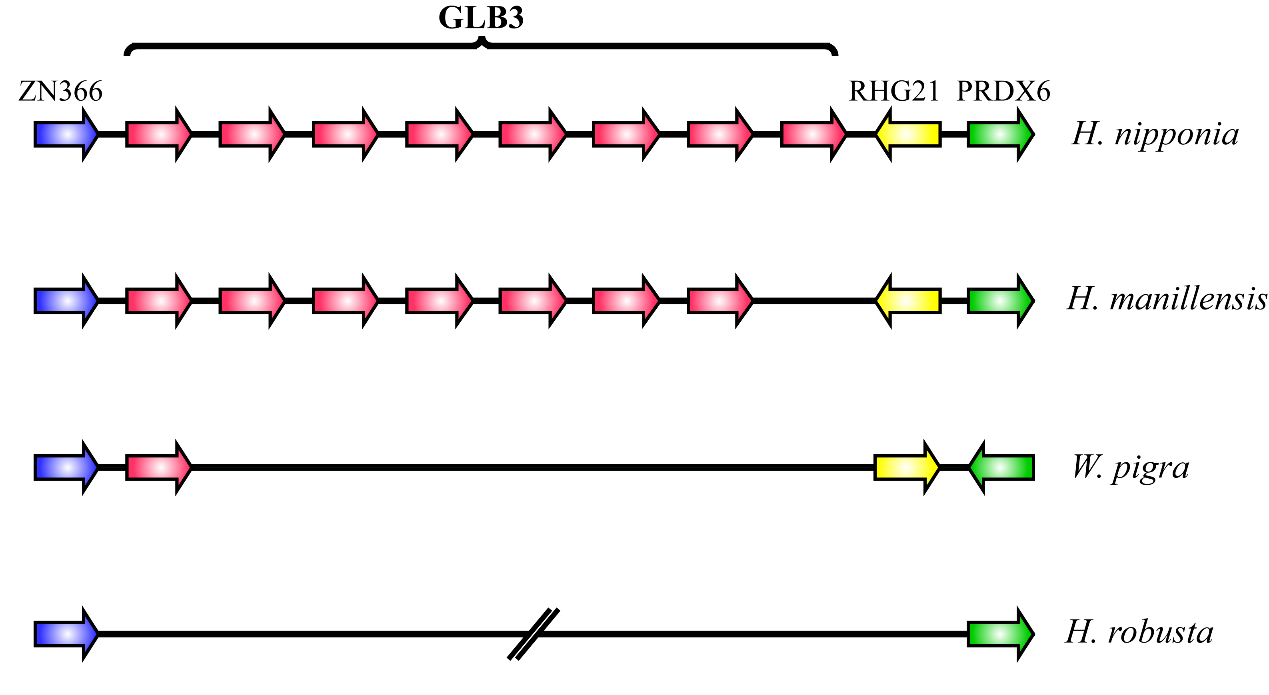
**Fig. S13.** ***GLB3* copies in four leech genomes.** Seven or eight *GLB3* copies were detected in bloodsucking leeches (top two species), and one or zero copy was found in non-bloodsucking leeches.

**Table S1. Comparison of the assembled genomes with other published leech genomes.**

| **Species** | **Total genome size (Mb)** | **Scaffold Number** | **Scaffold N50** | **Number of genes** |
| --- | --- | --- | --- | --- |
| *H. nipponia* | 203.7 | 11+253 | 18.5 MB | 20,430 |
| *H. manillensis* | 157.5 | 13+243 | 11.9 MB | 18,106 |
| *W. Pigra* | 181.4 | 11+183 | 16.2 MB | 18,540 |
| *H. medicinalis* (GenBank accession number: GCA_903470615.1) | 177.0 | 19,929 | 50.4 KB | 35,166 |
| *H. medicinalis* (GCA_011800805.1) | 187.6 | 14,042 | 97.8 KB | 14,596 |
| *H. manillensis* (ASM1534595v1) | 151.8 | 467 | 2.3 MB | 17,865 |
| *H. verbana* (GCA_020137395.1) | 235.0 | 59,817 | 8.4 KB | - |
| *W. pigra* (GCA_021613335.1) | 178.8 | 483 | 2.0 MB | - |
| *W. pigra* (GCA_021650995.1) | 177 | 10,050 | 728.0 KB | 26,743 |
| *H. robusta* (GCA_000326865.1) | 228 | 1,991 | 3.6MB | 23,400 |

**Table S2. Assessment of genome completeness and base accuracy on the basis of Illumina reads.**

|  | ***H. nipponia*** | ***H. manillensis*** | ***W. pigra*** |
| --- | --- | --- | --- |
| **Number of reads** | 133,418,728 | 106,586,450 | 129,361,718 |
| **Mapped reads** | 131,243,192 | 104,394,994 | 127,605,357 |
| **Mapping rate (%)** | 98.4 | 98 | 98.6 |
| **Covered site** | 199,000,381 | 153,548,517 | 176,639,994 |
| **Coverage rate (%)** | 97.7 | 97.7 | 97.5 |
| **Percentage of homozygous SNPs** | 4.60E-05 | 5.80E-05 | 3.50E-05 |

**Table S3. Mapping ratio of two random transcriptome assemblies for each of the three leech species.**

| **Species** | **Sample** | **Number of mapped assemblies** | **Number of assemblies** | **Ratio** |
| --- | --- | --- | --- | --- |
| *H. nipponia* | HNYB | 133,183 | 135,280 | 98.4% |
|  | HNYF | 119,821 | 122,180 | 98.0% |
| *H. manillensis* | HMSB | 118,624 | 119,857 | 99.0% |
|  | HMSF | 109,130 | 110,490 | 98.8% |
| 1. *pigra* | WPB | 104,410 | 106,131 | 98.3% |
|  | WPO | 96,711 | 101,454 | 95.3% |

**Table S4. BUSCO evaluation of the draft assemblies by using the metazoa_odb9 (2016-02-13) database.**

|  | ***H. nipponia*** | | ***H. manillensis*** | | ***W. pigra*** | |
| --- | --- | --- | --- | --- | --- | --- |
|  | Number | Ratio | Number | Ratio | Number | Ratio |
| **Complete** | 895 | 91.5% | 888 | 90.8% | 897 | 91.7% |
| **Fragmented** | 20 | 2.0% | 22 | 2.2% | 24 | 2.5% |
| **Missing** | 63 | 6.5% | 68 | 7.0% | 57 | 5.8% |
| **Total** | 978 | 100.0% | 978 | 100% | 978 | 100.0% |

**Table S5. Summary of the repeat contents in three leech genomes.**

|  | ***H. nipponia*** | | ***H. manillensis*** | | ***W. pigra*** | |
| --- | --- | --- | --- | --- | --- | --- |
|  | **Length** | **Ratio** | **Length** | **Ratio** | **Length** | **Ratio** |
| **DNA** | 14,620,931 | 7.2% | 3,934,101 | 2.5% | 10,484,088 | 5.8% |
| **LINE** | 14,291,581 | 7.0% | 6,495,956 | 4.1% | 8,937,369 | 4.9% |
| **LTR** | 5,227,039 | 2.6% | 6,398,533 | 4.1% | 6,678,890 | 3.7% |
| **SINE** | 551,769 | 0.3% | 203,223 | 0.1% | 344,292 | 0.2% |
| **Unknown** | 17,320,240 | 8.5% | 6,013,357 | 3.8% | 12,017,072 | 6.6% |
| **Other** | 16,621,036 | 8.2% | 16,757,206 | 10.7% | 16,677,692 | 9.2% |
| **Total** | 68,632,596 | 33.7% | 39,802,376 | 25.3% | 55,139,403 | 30.5% |

**Table S6. Statistics of predicted protein-coding genes in *H. nipponia* genome.**

| **Approach** | | **Gene number** | **Total CDS length (Mb)** | **Average CDS length (bp)** | **Average exons per gene** | **Average exon length (bp)** | **Average intron length (bp)** |
| --- | --- | --- | --- | --- | --- | --- | --- |
| ***De novo*** | Augustus | 18,595 | 32.3 | 1739 | 8.4 | 260 | 529 |
|  | SNAP | 40,996 | 35.1 | 856 | 4.8 | 180 | 306 |
| **Homology** | *C. teleta* | 18,177 | 11.6 | 638 | 3.0 | 211 | 453 |
|  | *E. andrei* | 17,628 | 15.9 | 902 | 4.2 | 214 | 540 |
|  | *H. robusta* | 16,786 | 12.1 | 723 | 4.1 | 178 | 446 |
| **Transcriptome** | StringTie | 22,583 | 48.8 | 2159 | 7.8 | 278 | 529 |
| **EVM** |  | 20,430 | 33.9 | 1660 | 8.4 | 197 | 511 |

**Table S7. Statistics of predicted protein-coding genes in *H. manillensis* genome.**

| **Approach** | | **Gene number** | **Total CDS length (Mb)** | **Average CDS length (bp)** | **Average exons per gene** | **Average exon length (bp)** | **Average intron length (bp)** |
| --- | --- | --- | --- | --- | --- | --- | --- |
| ***De novo*** | Augustus | 16634 | 27.4 | 1647 | 8.8 | 187 | 459 |
|  | SNAP | 33157 | 27.5 | 828 | 5.0 | 165 | 285 |
| **Homology** | *C. teleta* | 17693 | 11.0 | 623 | 3.1 | 202 | 412 |
|  | *E. andrei* | 17216 | 15.3 | 886 | 4.3 | 207 | 507 |
|  | *H. robusta* | 16837 | 11.8 | 702 | 4.1 | 174 | 405 |
| **Transcriptome** | StringTie | 19691 | 49.2 | 2500 | 8.6 | 290 | 476 |
| **EVM** |  | 18106 | 28.9 | 1596 | 8.9 | 180 | 442 |

**Table S8. Statistics of predicted protein-coding genes in *W. pigra* genome.**

| **Approach** | | **Gene number** | **Total CDS length (Mb)** | **Average CDS length (bp)** | **Average exons per gene** | **Average exon length (bp)** | **Average intron length (bp)** |
| --- | --- | --- | --- | --- | --- | --- | --- |
| ***De novo*** | Augustus | 17924 | 32.3 | 1801 | 9.0 | 201 | 509 |
|  | SNAP | 35942 | 29.5 | 820 | 4.6 | 178 | 259 |
| **Homology** | *C. teleta* | 17852 | 11.8 | 660 | 3.1 | 214 | 448 |
|  | *E. andrei* | 16072 | 16.2 | 1006 | 4.6 | 218 | 531 |
|  | *H. robusta* | 15614 | 12.0 | 766 | 4.3 | 179 | 423 |
| **Transcriptome** | StringTie | 17949 | 42.5 | 2369 | 9.0 | 264 | 476 |
| **EVM** |  | 18540 | 32.7 | 1764 | 9.0 | 197 | 494 |

**Table S9. Statistics of gene functional annotation of leech genomes.**

| **Category** | ***H. nipponia*** | | ***H. manillensis*** | | ***W. pigra*** | |
| --- | --- | --- | --- | --- | --- | --- |
|  | Number | Ratio | Number | Ratio | Number | Ratio |
| **GO** | 11970 | 58.6% | 10191 | 56.3% | 10967 | 59.2% |
| **InterPro** | 16071 | 78.7% | 13988 | 77.3% | 14714 | 79.4% |
| **KEGG** | 17371 | 85.0% | 15202 | 84.0% | 15909 | 85.8% |
| **SwissProt** | 14070 | 68.9% | 12734 | 70.3% | 13009 | 70.2% |
| **Trembl** | 17075 | 83.6% | 15167 | 83.8% | 15621 | 84.3% |
| **All** | 18038 | 88.3% | 15760 | 87.0% | 16424 | 88.6% |

**Table S10. Statistics of ncRNA annotation of *H. nipponia* genome.**

| **Category** | **Number** | **Average length (bp)** | **Total length (bp)** | **Percentage of genome (%)** |
| --- | --- | --- | --- | --- |
| miRNA | 330 | 156 | 51,598 | 0.0253 |
| snRNA | 784 | 144 | 113,589 | 0.0558 |
| rRNA | 260 | 204 | 53,258 | 0.0261 |
| tRNA | 4,821 | 77 | 375,673 | 0.1844 |

**Table S11. Statistics of ncRNA annotation of *H. manillensis* genome.**

| **Category** | **Number** | **Average length (bp)** | **Total length (bp)** | **Percentage of genome (%)** |
| --- | --- | --- | --- | --- |
| miRNA | 288 | 154 | 44,445 | 0.0283 |
| snRNA | 703 | 152 | 107,503 | 0.0684 |
| rRNA | 201 | 113 | 22,907 | 0.0146 |
| tRNA | 3,843 | 77 | 298,934 | 0.1903 |

**Table S12. Statistics of ncRNA annotation of *W. pigra* genome.**

| **Category** | **Number** | **Average length (bp)** | **Total length (bp)** | **Percentage of genome (%)** |
| --- | --- | --- | --- | --- |
| miRNA | 271 | 159 | 43,126 | 0.0238 |
| snRNA | 690 | 151 | 104,456 | 0.0577 |
| rRNA | 178 | 377 | 67,269 | 0.0371 |
| tRNA | 3,848 | 78 | 302,368 | 0.167 |

**Table S13. Statistics of secreted protein in leech genomes.**

| **Species** | **Number** | **Ratio** |
| --- | --- | --- |
| *H. nipponia* | 1785 | 8.70% |
| *H. manillensis* | 1608 | 8.90% |
| *W. pigra* | 1629 | 8.80% |
| *H. robusta* | 1691 | 7.20% |

**Table S14. Copy number of genes related to prey tracking and location.**

|  | ***H. manillensis*** | ***H. nipponia*** | ***W. pigra*** | ***H. robusta*** |
| --- | --- | --- | --- | --- |
| *RHO* | 1 | 1 | 1 | 1 |
| *GNAQ* | 2 | 1 | 1 | 2 |
| *PDE6D* | 0 | 0 | 1 | 1 |
| *PAX6* | 2 | 2 | 2 | 2 |
| *TRPA1* | 2 | 2 | 2 | 2 |
| *SIX1* | 2 | 4 | 1 | 1 |
| *EYA1* | 1 | 1 | 1 | 1 |
| *ACHA9* | 2 | 3 | 3 | 3 |
| *GATA3* | 4 | 4 | 3 | 6 |
| *CHD7* | 2 | 3 | 1 | 3 |
| **Total** | 18 | 21 | 16 | 22 |

**Table S15. Copy number of genes related to bloodsucking characteristics.**

|  | ***H. manillensis*** | ***H. nipponia*** | ***W. pigra*** | ***H. robusta*** |
| --- | --- | --- | --- | --- |
| *AGRN* | 5 | 7 | 6 | 10 |
| *ANTA* | 5 | 8 | 5 | 5 |
| *APY* | 0 | 2 | 0 | 1 |
| *ADAMTS18* | 4 | 2 | 1 | 0 |
| *CA2* | 4 | 5 | 3 | 6 |
| *CYT* | 1 | 2 | 0 | 3 |
| *DECO* | 0 | 1 | 0 | 0 |
| *FBN1* | 12 | 12 | 4 | 0 |
| *GLB3* | 7 | 8 | 1 | 0 |
| *HIRM1* | 2 | 1 | 1 | 0 |
| *ICIC* | 4 | 6 | 3 | 1 |
| *SERPINB1* | 8 | 3 | 7 | 6 |
| *LDLR* | 3 | 2 | 3 | 6 |
| *LHYAL* | 5 | 7 | 4 | 1 |
| *LRP1* | 6 | 6 | 2 | 5 |
| *MMEL1* | 8 | 15 | 6 | 1 |
| *MMP13* | 1 | 2 | 2 | 2 |
| *NEP1* | 2 | 8 | 4 | 5 |
| *PROS1* | 5 | 5 | 6 | 3 |
| *TLR4* | 7 | 2 | 6 | 3 |
| **Total** | 89 | 104 | 64 | 58 |
