# Supplementary material for: Molecular mechanisms underlying hematophagia revealed by comparative analyses of leech genomes
Source: Gigascience. 2023 Apr 11;12:giad023. doi: 10.1093/gigascience/giad023 (PMC10087013; doi:10.1093/gigascience/giad023)
Supplement: giad023_Response_to_Reviewer_Comments_Original_Submission [file giad023_response_to_reviewer_comments_original_submission.pdf]

Author's Response To Reviewer Comments

Close

Dec. 14, 2022

To
The Editorial Office,
GigaScience

Dear editors and reviewers:

Thank you for your kind reply. We are very grateful for the reviewer's comments on our manuscript entitled "Molecular mechanisms underlying hematophagia revealed by comparative analyses of leech genomes" (Manuscript id: GIGA-D-22-00200). Indeed, your comments are very helpful in improving our manuscript. We have carefully studied your comments and tried our best to revise the manuscript to meet your approval. We think we have finished all concerns from reviewers. Here, the revisions are documented in the revised manuscript, response letter, and Supplementary Figs and Tables. The responses to the reviewers' comments are as follows.

Kind regards,

Qingyou Liu

E-mail address: qyliu-gene@gxu.edu.cn

Response to reviewers

Reviewer #1:
This study entitled "Molecular mechanisms underlying hematophagia revealed by comparative analyses of leech genomes" has reported three chromosome-level genomes of leeches. This study has shown population changes of three leeches, and comparative analysis result of copy number of some special gene families has found some clues for divergence between bloodsucking and nonbloodsuking behaviours. The genome materials and findings of this study are indeed significant.
The quality of these three genomes are pretty well. The analysed approaches used in this paper are also well performed.

Response: Thank you very much for your supportive comments. We sincerely appreciate for the valuable comments from you.

Nevertheless, the English language of this paper indeed need to be largely revised by a native speaker. Too much errors were appeared in this paper.

Response: Thank you for your suggestion. The manuscript has been revised by professional editing company ELIXIGEN to improve the writing expression. They are highlighted with red colors and underlines. The editorial certification is listed below.

The detailed version and used library of BUSCO should be described, and the annotation BUSCO number also should be reported.

Response: The BUSCO library we used is metazoa_odb9 (2016-02-13), and the detailed BUSCO number and total BUSCO number are listed in Table S4. Please see below.

Table S4. BUSCO evaluation of the draft assemblies using metazoa_odb9 (2016-02-13) database.
H. nipponia H. manillensis W. pigra
Number Ratio Number Ratio Number Ratio
Complete 895 91.5% 888 90.8% 897 91.7%
Fragmented 20 2.0% 22 2.2% 24 2.5%
Missing 63 6.5% 68 7.0% 57 5.8%
Total 978 100.0% 978 100% 978 100.0%

The format of this study should be largely revised, and RRIDs of used software should be added in this manuscript.

Response: We follows the “Data Note” format in gigascience journal to revise our manuscript as editor suggested, and all the available software RRIDs are added.

In figure 1B, the inner aligned lines only contain the alignments between H. nipponia and H. manillensis, and H. nipponia and W. pigra. Why the alignments between H. man and W.pigra are not shown in this figure?

Response: Sorry for lack of the alignments between H. manillensis and W.pigra. Now we added it. Please check it below in figure1B.

In figure 1C, I suggest authors to add the sea-level and air temperature curves in this figure.
The detailed curves you can reference this paper, Whole Genome Sequencing of Chinese White Dolphin (Sousa chinensis) for High-Throughput Screening of Antihypertensive Peptides

Response: That’s a good point. We have added the curves in figure1C. Please check it above.

Some minor suggestions:
"Here we provided three high-quality leech genomes and abundant transcriptomes which illustrated the gene expression dynamics of bloodsucking leeches including anticoagulation, analgesic, and anti-inflammation that would facilitate the understanding at the genetic level and could be crucial for drug candidate prospecting" revised to 'Here we provided three high-quality leech genomes and abundant transcriptomes that illustrated the gene expression dynamics of bloodsucking leeches including anticoagulation, analgesic, and anti-inflammation, which would facilitate the understanding at the genetic level and could be crucial for drug candidate prospecting'.

Response: Thank you for your suggestion. It has been corrected and highlighted with yellow in the manuscript.

For "contained 985, 622, and 437 Mb contigs with N50 contig lengths of 1.1, 2.5, and 4.1 Mb", the decimal places through whole paper should be uniformed. Moreover, this could be an error description for '985, 622 and 437 Mb contigs'. In my opinion, 'Mb' should be removed, there is the number of contigs.

Response: Thanks for pointing this out. The decimal places are now kept to one through the whole paper. “985, 622 and 437” indeed represent the number of contigs, and ‘Mb’ has been removed.

'and we identified' changed to 'to identify'.

Response: Change made.

"we constructed the gene family" changed to "we constructed gene families".

Response: Change made.

'20430, 18106, and 18540 protein-coding genes' should be '20,430, 18,106, and 18,540 protein-coding genes'

Response: Corrected.

'restored to that' changed to 'restored to those'.

Response: Change made.

'is fundamental to understanding' revised to 'is fundamental to understand'.

Response: Corrected.

Reviewer #2: This manuscript provides some useful genomic and transcriptomic data of three leech species. And the bloodsucking characteristics of leeches are explained to some extent. However, the present version does not meet the publish criteria of GigaScience. Here are my detailed comments which may help to improve the manuscripts before it can be accepted for publication.

Response: Many thanks for your professional comments. We appreciate your time and constructive suggestions, which is very helpful for improving our manuscript. We hope our revisions in this version could meet your criteria and further receive your replies. Please check our improvements as follows.

1.Correct species classification is crucial for scientific papers. I see the author states that leeches are "arthropods" in the first sentence. It is a trivial but serious mistake, because it has long been accepted by researchers that leeched belong to Annelida. Hence it is reasonable for readers to suspect the validity of taxonomic status of all specimen involved in this manuscript. I strongly suggest that the authors firstly taking both a morphological and a molecular identification of all their leech specimens.

Response: We feel very sorry for that mistake and definitely agree with your opinion. Now we have corrected and changed “arthropods” to “annelids”.

2.As mentioned in the title as well as the whole text, the main interest of this manuscript focus on the molecular mechanisms of hematophagous behavior of leech. However, the most characteristic anticoagulants of leech such as hirudin is poorly discussed, e.g. gene structure, sequence specificity. On the contrary, FBN1 and GLB3, which is mainly discussed in the manuscript, is farfetched to explain the blood sucking habit.

Response: Thank you for pointing this out. Following your suggestions, we performed analysis on hirudin genes about gene structure, sequence specificity and gene expression pattern. Three hirudin genes were identified in H. manillensis, and one is identified in both H. nipponia and W. Pigra. The intron regions of HN_hirudin and WP_hirudin are inserted with transposable elements (TEs), leading to longer length than the three HM_hirudin genes without TE insertions (Fig.S11A below). Multiple sequence alignments show that the cysteine pattern of the hirudin is more conserved than the tail but less conserved than the beginning of the alignment (Fig.S11B below). Finally, we find that hirudin genes mainly express in the oral suckers of the three leeches, and the expression of HM_hiridin1 is comparatively increased at the time point of 30 minutes (Fig.S11C), which is consistent with the time of physiological coagulation.

Fig. S11. Analysis of hirudin genes in the three leeches.

We noticed that HM_hirudin3 shows “redundant” sequence in the region of the cysteine pattern. We checked the alignment of the transcriptome data and confirmed the authenticity of the “redundant” sequence (figure below). It suggests that the cysteine pattern is presumably highly plastic.

We thus described our revisions in this main text as follows:
“The gene structure and copy number of the well-known anticoagulant HIRM1 (hirudin) were different in the three leeches (Supplementary Fig. 11). The gene lengths of HN_hirudin and WP_hirudin were apparently longer than the three HM_hirudin copies, and this was mainly attributable to TE insertion into intron regions (Supplementary Fig. 11A). We performed multiple sequence alignment for five HIRM1 genes and found that the cysteine pattern of hirudin [7] is more conserved than the tail but less conserved than the beginning of the alignment (Supplementary Fig. 11B). This indicates that the cysteine pattern may have the potential to be plastic. HIRM1 was mainly expressed in the oral suckers of the three leech species, and the expression of HM_hiridin1 comparatively increased at 30 min (Supplementary Fig. 11C), which is consistent with the time of physiological coagulation.”

3. The authors mentioned in the Introduction "Currently, a few leech species genomic data including one non-blood sucking leech (Helobdella robusta) and low coverage genome sequence data of two lineages Amynthas cortices have been published [3-6],…". Actually, besides Helobdella robusta there have been four leech species deposited in the GenBank: Hirudo medicinalis (accession numbers: GCA_011800805.1, GCA_903470615.1); Hirudo verbana (GCA_020137395.1); Hirudinaria manillensis (ASM1534595v1); Whitmania pigra (GCA_021613335.1, GCA_021650995.1). The latter two overlapped with the species used in this manuscript. It is clear that the authors noticed the GenBank data since H. medicinalis and H. manillensis are listed in the Table S1. It is not clear, however, that why they drop two more famous species H. verbana and W. pigra, but instead using a distantly related species Helobdella robusta and even earthworms?

Response: We apologize for the incomplete collection of genomic information. We change the sentence to “the genomic data of several leech species have been published” and add all the genomic information of the mentioned species into Table S1 as well as the available references into the main content.

Table S1. Comparison of genome assembly to other publicized leech genomes.
Species Total genome size (Mb) Scaffolds Number Scaffold N50 Number of genes
H. nipponia 203.7 11+253 18.5 MB 20,430
H. manillensis 157.5 13+243 11.9 MB 18,106
W. Pigra 181.4 11+183 16.2 MB 18,540
H. medicinalis (Genbank accession numbers:GCA_903470615.1) 177.0 19,929 50.4 KB 35,166
H. medicinalis (GCA_011800805.1) 187.6 14,042 97.8 KB 14,596
H. manillensis (ASM1534595v1) 151.8 467 2.3 MB 17,865
H. verbana (GCA_020137395.1) 235.0 59,817 8.4 KB -
W. pigra (GCA_021613335.1) 178.8 483 2.0 MB -
W. pigra (GCA_021650995.1) 177 10,050 728.0 KB 26,743
H. robusta (GCA_000326865.1) 228 1,991 3.6MB 23,400

Additional reference:
Tong, Lei, et al. "The genome of medicinal leech (Whitmania pigra) and comparative genomic study for exploration of bioactive ingredients." BMC Genomics 23.1 (2022): 1-13.

4.There are obvious differences in genome size between different species and different papers of the same species. It is suggested that the authors explaining the reason or examine whether there is DNA contamination from microorganisms or other species. For example, the H. nipponia has a much larger genome than the other species in Hirudinidae. Is there potential contamination by bacteria genome?

Response: In our analysis, we removed these contigs that covered more than 50% of the bacterial genome sequences deposited in NCBI (see the method “Genome assembly and assessment”). Thus, bacterial contamination likely has little effect on genome size in this study. In recent studies on large genomes, such as african lungfish[1] (~40Gb) and Mexican axolotl[2] (~32Gb), TEs mainly contribute to their huge genomes. In our leech genomes, the maximum difference in the lengths of the TE sequences is 28.8Mb (accounts for 62.3% of the difference of genome sizes) between H. nipponia, and H. manillensis, suggesting the TE contents maybe a major driver to the larger leech genome. Consistent with the speculation, TEs are responsible for the intron expansion in hirudin genes of H. nipponia and W. pigra compared to them of H. manillensis (Question 3).

For the same species, it is known that the genome size and contig N50 could be improved by using more sequencing reads. For example, we provided ~48 Gb compared with ~12Gb in Guan et al.[3] for H. manillensis, and we obtained more ~5.7Mb genome sequences as well as longer contig N50 (2.5Mb vs 2.3Mb). Besides, long-read sequencing technology are usually used to resolve many repeats longer than the second-sequencing reads, leading to more complete assembly results. For example, we assembled larger genome (181.4Mb vs 177Mb) for W. pigra and more repetitive contents (30.5% vs 23%) than Tong et al[4]. In addition, the different assembling strategies applied in different papers presumably partly contribute to the difference of genome sizes for the same species.

[1]Wang, Kun, et al. "African lungfish genome sheds light on the vertebrate water-to-land transition." Cell 184.5 (2021): 1362-1376.
[2]Nowoshilow, Sergej, et al. "The axolotl genome and the evolution of key tissue formation regulators." Nature 554.7690 (2018): 50-55.
[3]Guan, De-Long, et al. "Draft genome of the Asian buffalo leech Hirudinaria manillensis." Front. Genet. 10 (2020): 1321.
[4]Tong, Lei, et al. "The genome of medicinal leech (Whitmania pigra) and comparative genomic study for exploration of bioactive ingredients." BMC Genomics 23.1 (2022): 1-13.

5.The number of chromosome of leech varies between species. The HI-C analysis in this work was based on an assumed number of these three species, which could lead to a specious result. It is recommended that the author provide verification on the chromosome number of those new reported genomes before they draw conclusions from the HI-C analysis.

Response: We apologize that the software used for contig clustering was incorrectly written as Lachesis (the corresponding reference is about 3D-DNA software), which is in fact 3D-DNA. Just as you said, Lachesis indeed was based on assumed chromosome number. 3D-DNA automatically cluster the contigs without setting chromosome numbers. We tried to identify their karyotypes using experimental method but failed previously, so we describe the the HiC clustering results as “pseudo-chromosomes”.

Close
