# Supplementary material for: Molecular mechanisms underlying hematophagia revealed by comparative analyses of leech genomes
Source: Gigascience. 2023 Apr 11;12:giad023. doi: 10.1093/gigascience/giad023 (PMC10087013; doi:10.1093/gigascience/giad023)
Supplement: giad023_Reviewer_1_Report_Original_Submission — Chao Bian -- 8/25/2022 Reviewed [file giad023_reviewer_1_report_original_submission.pdf]

Reviewer Report

Title: Molecular mechanisms underlying hematophagia revealed by comparative analyses of leech genomes

Version: Original Submission **Date:** 8/25/2022

Reviewer name: Chao Bian

Reviewer Comments to Author:

This study entitled "Molecular mechanisms underlying hematophagia revealed by comparative analyses of leech genomes" has reported three chromosome-level genomes of leeches. This study has shown population changes of three leeches, and comparative analysis result of copy number of some special gene families has found some clues for divergence between bloodsucking and nonbloodsuking behaviours. The genome materials and findings of this study are indeed significant.The quality of these three genomes are pretty well. The analysed approaches used in this paper are also well performed.Nevertheless, the English language of this paper indeed need to be largely revised by a native speaker. Too much errors were appeared in this paper.The detailed version and used library of BUSCO should be described, and the annotation BUSCO number also should be reported.The format of this study should be largely revised, and RRIDs of used software should be added in this manuscript.In figure 1B, the inner aligned lines only contain the alignments between H. nipponia and H. manillensis, and H. nipponia and W. pigra. Why the alignments between H. man and W.pigra are not shown in this figure?In figure 1C, I suggest authors to add the sea-level and air temperature curves in this figure.The detailed curves you can reference this paper, Whole Genome Sequencing of Chinese White Dolphin (Sousa chinensis) for High-Throughput Screening of Antihypertensive PeptidesSome minor suggestions:"Here we provided three high-quality leech genomes and abundant transcriptomes which illustrated the gene expression dynamics of bloodsucking leeches including anticoagulation, analgesic, and anti-inflammation that would facilitate the understanding at the genetic level and could be crucial for drug candidate prospecting" revised to 'Here we provided three high-quality leech genomes and abundant transcriptomes that illustrated the gene expression dynamics of bloodsucking leeches including anticoagulation, analgesic, and anti-inflammation, which would facilitate the understanding at the genetic level and could be crucial for drug candidate prospecting'.For "contained 985, 622, and 437 Mb contigs with N50 contig lengths of 1.1, 2.5, and 4.1 Mb", the decimal places through whole paper should be uniformed. Moreover, this could be an error description for '985, 622 and 437 Mb contigs'. In my opinion, 'Mb' should be removed, there is the number of contigs.'and we identified' changed to 'to identify'."we constructed the gene family" changed to "we constructed gene families".'20430, 18106, and 18540 protein-coding genes' should be '20,430, 18,106, and 18,540 protein-coding genes''restored to that' changed to 'restored to those'.'is fundamental to understanding' revised to 'is fundamental to understand'.

Level of Interest

Please indicate how interesting you found the manuscript: Choose an item.

Quality of Written English

Please indicate the quality of language in the manuscript: Choose an item.

Declaration of Competing Interests

Please complete a declaration of competing interests, considering the following questions:

- Have you in the past five years received reimbursements, fees, funding, or salary from an organisation that may in any way gain or lose financially from the publication of this manuscript, either now or in the future?
- Do you hold any stocks or shares in an organisation that may in any way gain or lose financially from the publication of this manuscript, either now or in the future?
- Do you hold or are you currently applying for any patents relating to the content of the manuscript?
- Have you received reimbursements, fees, funding, or salary from an organization that holds or has applied for patents relating to the content of the manuscript?
- Do you have any other financial competing interests?
- Do you have any non-financial competing interests in relation to this paper?

If you can answer no to all of the above, write 'I declare that I have no competing interests' below. If your reply is yes to any, please give details below.

I declare that I have no competing interests

I agree to the open peer review policy of the journal. I understand that my name will be included on my report to the authors and, if the manuscript is accepted for publication, my named report including any attachments I upload will be posted on the website along with the authors' responses. I agree for my report to be made available under an Open Access Creative Commons CC-BY license (http://creativecommons.org/licenses/by/4.0/). I understand that any comments which I do not wish to be included in my named report can be included as confidential comments to the editors, which will not be published.

Choose an item.

To further support our reviewers, we have joined with Publons, where you can gain additional credit to further highlight your hard work (see: https://publons.com/journal/530/gigascience). On publication of this paper, your review will be automatically added to Publons, you can then choose whether or not to claim your Publons credit. I understand this statement.

Yes Choose an item.
