# Supplementary material for: Molecular mechanisms underlying hematophagia revealed by comparative analyses of leech genomes
Source: Gigascience. 2023 Apr 11;12:giad023. doi: 10.1093/gigascience/giad023 (PMC10087013; doi:10.1093/gigascience/giad023)
Supplement: giad023_Reviewer_2_Report_Original_Submission — zichao liu -- 9/19/2022 Reviewed [file giad023_reviewer_2_report_original_submission.pdf]

Reviewer Report

Title: Molecular mechanisms underlying hematophagia revealed by comparative analyses of leech genomes

Version: Original Submission **Date:** 9/19/2022

Reviewer name: zichao liu

Reviewer Comments to Author:

This manuscript provides some useful genomic and transcriptomic data of three leech species. And the bloodsucking characteristics of leeches are explained to some extent. However, the present version does not meet the publish criteria of GigaScience. Here are my detailed comments which may help to improve the manuscripts before it can be accepted for publication.1. Correct species classification is crucial for scientific papers. I see the author states that leeches are "arthropods" in the first sentence. It is a trivial but serious mistake, because it has long been accepted by researchers that leeched belong to Annelida. Hence it is reasonable for readers to suspect the validity of taxonomic status of all specimen involved in this manuscript. I strongly suggest that the authors firstly taking both a morphological and a molecular identification of all their leech specimens.2. As mentioned in the title as well as the whole text, the main interest of this manuscript focus on the molecular mechanisms of hematophagous behavior of leech. However, the most characteristic anticoagulants of leech such as hirudin is poorly discussed, e.g. gene structure, sequence specificity. On the contrary, FBN1 and GLB3, which is mainly discussed in the manuscript, is farfetched to explain the blood sucking habit.3. The authors mentioned in the Introduction "Currently, a few leech species genomic data including one non-blood sucking leech (Helobdella robusta) and low coverage genome sequence data of two lineages Amynthas cortices have been published [3-6],…". Actually, besides Helobdella robusta there have been four leech species deposited in the GenBank: Hirudo medicinalis (accession numbers: GCA_011800805.1, GCA_903470615.1); Hirudo verbana (GCA_020137395.1); Hirudinaria manillensis (ASM1534595v1); Whitmania pigra (GCA_021613335.1, GCA_021650995.1). The latter two overlapped with the species used in this manuscript. It is clear that the authors noticed the GenBank data since H. medicinalis and H. manillensis are listed in the Table S1. It is not clear, however, that why they drop two more famous species H. verbana and W. pigra, but instead using a distantly related species Helobdella robusta and even earthworms?4. There are obvious differences in genome size between different species and different papers of the same species. It is suggested that the authors explaining the reason or examine whether there is DNA contamination from microorganisms or other species. For example, the H. nipponia has a much larger genome than the other species in Hirudinidae. Is there potential contamination by bacteria genome?5.The number of chromosome of leech are various between species. The HIC analysis in this work was based on an assumed number of these three species, which could lead to a specious result. It is recommended that the author provide verification on the chromosome number of those new reported genomes before they draw conclusions from the HIC analysis.

Level of Interest

Please indicate how interesting you found the manuscript: Choose an item.

Quality of Written English

Please indicate the quality of language in the manuscript: Choose an item.

Declaration of Competing Interests

Please complete a declaration of competing interests, considering the following questions:

- Have you in the past five years received reimbursements, fees, funding, or salary from an organisation that may in any way gain or lose financially from the publication of this manuscript, either now or in the future?
- Do you hold any stocks or shares in an organisation that may in any way gain or lose financially from the publication of this manuscript, either now or in the future?
- Do you hold or are you currently applying for any patents relating to the content of the manuscript?
- Have you received reimbursements, fees, funding, or salary from an organization that holds or has applied for patents relating to the content of the manuscript?
- Do you have any other financial competing interests?
- Do you have any non-financial competing interests in relation to this paper?

If you can answer no to all of the above, write 'I declare that I have no competing interests' below. If your reply is yes to any, please give details below.

I declare that I have no competing interests.

I agree to the open peer review policy of the journal. I understand that my name will be included on my report to the authors and, if the manuscript is accepted for publication, my named report including any attachments I upload will be posted on the website along with the authors' responses. I agree for my report to be made available under an Open Access Creative Commons CC-BY license (http://creativecommons.org/licenses/by/4.0/). I understand that any comments which I do not wish to be included in my named report can be included as confidential comments to the editors, which will not be published.

Choose an item.

To further support our reviewers, we have joined with Publons, where you can gain additional credit to further highlight your hard work (see: https://publons.com/journal/530/gigascience). On publication of this paper, your review will be automatically added to Publons, you can then choose whether or not to claim your Publons credit. I understand this statement.

Yes Choose an item.
